# Effect of Herbal Extracts on Antituberculosis Drug‐Induced Hepatotoxicity: A Systematic Review

**DOI:** 10.1155/jt/6698835

**Published:** 2026-09-29

**Authors:** Surthi M. Ravedar, Kranthi Kiran Akula, Kalesh M. Karun, Flemin Felix, Kamran Zaman, Jyothi Bhat, Subarna Roy

**Affiliations:** ^1^ ICMR National Institute of Traditional Medicine, Belagavi 590010, Karnataka, India; ^2^ KLE Academy of Higher Education and Research, Belagavi 590010, Karnataka, India

**Keywords:** anti-TB drugs, drug-induced liver injury, drug toxicity, hepatoprotective, hepatotoxicity, herbal extract, plant extract, tuberculosis

## Abstract

**Background:**

Tuberculosis (TB) remains a major global health challenge. While effective, first‐line antitubercular drugs such as isoniazid and rifampicin often cause hepatotoxicity, limiting treatment safety. Herbal extracts with antioxidant and hepatoprotective properties may help mitigate this liver damage. This systematic review evaluates the protective effects of plant extracts against antitubercular drug‐induced hepatotoxicity, aiming to inform potential adjuvant strategies.

**Methods:**

Following PRISMA‐P guidelines, PubMed, Scopus, and Google Scholar were searched for “in vivo studies assessing plant extracts against hepatotoxicity caused by anti‐TB drugs.” Two independent reviewers screened and extracted data. Study quality was evaluated using SYRCLE’s risk of bias tool.

**Results:**

Sixty‐eight in vivo studies were included. Overall, plant extracts demonstrated variable hepatoprotective efficacy, with some showing significant improvement in certain species—*Luffa acutangula* L.*, Lawsonia inermis* L., and *Cucumis trigonus* Roxb. showed the highest reduction in serum liver enzymes: aspartate aminotransferase (AST), alanine transaminase (ALT), alkaline phosphatase (ALP) in U/L, and total bilirubin (TBil) and antioxidant parameters glutathione (GSH), catalase (CAT), and superoxide dismutase (SOD), suggesting hepatoprotective potential.

**Conclusion:**

Medicinal plants demonstrate variable hepatoprotective effects in animal models of anti‐TB drug‐induced liver injury, though their mechanisms of oxidative stress remain unclear. Among them, the extracts from *Luffa acutangula* L., *Lawsonia inermis* L., and *Cucumis trigonus* Roxb. showed promising hepatoprotective effects. These effects are likely associated with antioxidant and cytoprotective mechanisms. However, further well‐designed preclinical and clinical research are required to establish their dosage, safety, and efficacy before integration into TB therapy.

**Trial Registration:** PROSPERO: CRD42024563060.

## 1. Introduction

Tuberculosis (TB), caused by *Mycobacterium tuberculosis* (MTB), remains a major public health concern and one of the leading causes of infectious disease‐related mortality worldwide. Despite the availability of vaccines and effective antitubercular drugs and treatment, 10.6 million new TB cases and 1.5 million deaths are reported annually [1, 2]. The standard directly observed treatment short‐course (DOTS) protocol—comprising isoniazid (INH), rifampicin (RIF), pyrazinamide (PZA), and ethambutol (EMB)—is highly effective in controlling TB; however, its clinical utility is frequently limited by adverse reactions, particularly antitubercular drug–induced hepatotoxicity [3].

The liver is a multifunctional organ controlling vital processes involving metabolism, detoxification, and nutrient regulation [4]. As the primary organ of drug metabolism, the liver is highly susceptible to damage from pharmaceutical compounds due to the nonspecific activity of its versatile enzymes, often leading to drug‐induced hepatotoxicity [5]. Drug‐induced hepatotoxicity is a key problem that limits therapy and medication usage.

More than 900 medications, such as acetaminophen, chloroquine, and INH, can cause hepatotoxicity. First‐line antitubercular medicines cause hepatotoxicity predominantly through the formation of reactive metabolites via oxidative stress, mitochondrial dysfunction, and immune‐mediated pathways. N‐acetyltransferase 2 (NAT2) metabolizes INH in the liver to acetyl hydrazine and hydrazine, which undergo oxidation by cytochrome P450 2E1 (CYP2E1) into hepatotoxic intermediates that generate reactive oxygen species (ROS), resulting in lipid peroxidation, mitochondrial damage, and hepatocyte apoptosis or necrosis. RIF exacerbates INH‐induced hepatotoxicity by inducing hepatic CYP enzymes (particularly CYP3A4 and CYP2E1), thereby increasing the toxic metabolites. RIF also disrupts bile acid homeostasis via pregnane X receptor (PXR)‐mediated regulation, contributing to cholestatic liver injury. PZA gets converted to pyrazinoic acid and 5‐hydroxy‐pyrazinoic acid, which cause direct dose‐dependent hepatocellular damage, including hepatic inflammation, mitochondrial dysfunction, and necrosis. EMB has a low intrinsic hepatotoxic potential. It is infrequently associated with liver injury, with most clinical evidence indicating a minor role in hepatotoxicity when compared to INH, RIF, and PZA. These drugs cause liver injury through a combination of mechanisms, including oxidative stress, activation of metabolic enzymes, inflammatory signaling, and altered hepatic detoxification pathways. Recent investigations have shown a strong link between hepatic damage and oxidative stress in experimental animals treated with antituberculosis drugs [4, 5].

*Conventional management of antituberculosis drug-induced hepatotoxicity (ATDH)* includes temporary withdrawal and/or substitution of offending drugs, along with the use of hepatoprotective agents to mitigate liver injury. Among these, *silymarin*, a flavonolignan complex extracted from *Silybum marianum* (milk thistle), has been extensively studied for its hepatoprotective, antioxidant, and membrane‐stabilizing properties. It scavenges free radicals and enhances hepatic glutathione (GSH) levels, thereby protecting hepatocytes from damage caused by drugs such as INH and RIF. A randomized controlled trial demonstrated that *silymarin* significantly reduced the incidence of ATDH in patients undergoing anti‐TB therapy [6].

Another widely used formulation is “*Liv.52*,” a proprietary polyherbal product developed by the Himalaya Drug Company. It contains Mandur Bhasma and extracts of *Capparis spinosa*, *Cichorium intybus*, and *Solanum nigrum*, among others. *Liv.52* has been shown to normalize hepatic enzyme levels and reduce oxidative stress, thereby supporting liver function. A clinical study confirmed its hepatoprotective effects in TB patients, significantly reducing the levels of serum transaminase and improving liver function markers [7].

Plant‐derived formulations are increasingly used as supportive therapies alongside or following hepatotoxic anti‐TB drug regimens, especially in regions where herbal medicine is integrated into mainstream healthcare. Globally, herbal medications are gaining popularity due to their perceived safety, minimal side effects, and accessibility. In India, they are widely used in traditional medicine for managing liver complications. Numerous studies have investigated the potential of herbal or phytochemical compounds to counteract anti‐TB drug‐induced hepatotoxicity without interfering with the efficacy of drugs [8]. These formulations often contain bioactive phytochemicals that serve as hepatoprotective agents by inhibiting cytochrome P450 and microsomal drug‐metabolizing enzymes and also as antioxidants to reduce oxidative stress and tissue damage. References to such plant‐based hepatoprotective treatments are found in ancient indigenous medical systems and remain in practice among rural and tribal communities worldwide, including in India [9]. Given the well‐documented hepatotoxicity of first‐line antitubercular drugs and the promising evidence for the efficacy of certain plants, there is a critical need to explore plant‐based therapeutic alternatives.

Traditional medical systems offer a rich source of hepatoprotective plants with antioxidant and enzyme‐regulating properties. This systematic review aims to evaluate the efficacy of herbal extracts in preventing anti‐TB drug‐induced liver injury (DILI) and to provide evidence‐based insights into their potential role as adjunctive therapies in TB management.

## 2. Methodology

### 2.1. Protocol Registration

The review was developed according to the Preferred Reporting Items for Systematic Reviews and Meta‐analyses Protocol (PRISMA‐P) guidelines.

### 2.2. Exploration Approach of Primary Studies

The literature search was performed using electronic bibliographic databases Scopus, PubMed, and Google Scholar. Two reviewers independently reviewed the titles and abstracts for relevant articles, excluding irrelevant ones. Discrepancies were discussed or escalated to a neutral third party for a final determination. Later, the full‐text research articles satisfying the criteria were retrieved and separately examined by the two authors to determine the final set of studies for review. A thorough search was run using acceptable keywords to retrieve all relevant articles. For example, the search words used were “TB, White Plague, Koch’s Disease, Pulmonary tuberculosis, Lung TB,” and “Medicinal herbs, Traditional herbs, Ethnobotanical plants, Herbal medicines, Plant‐based therapies, Natural remedies, Traditional Medicinal Plants” and “Liver toxicity, Hepatic toxicity, Liver damage, Hepatocellular damage, Hepatic adverse effects, Drug‐induced hepatotoxicity, Hepatotoxicity” and “Tuberculosis medications, TB antibiotics, TB drugs, Anti‐tubercular drugs, Anti‐TB medications, Anti‐Mycobacterial drugs, Anti‐TB drugs” (Supporting table no. 1). All search results were imported into Rayyan QCRI software (Qatar Computing Research Institute, Hamad Bin Khalifa University, Doha, Qatar; Version 2.0) to ensure a thorough and systematic search and to document the selection process.

### 2.3. Inclusion and Exclusion Criteria

#### 2.3.1. Inclusion Criteria

• *Study design:* Comparative experimental in vivo studies conducted between 2002 and 2024
• *Intervention:* Plant extracts for hepatoprotective activity
• *Comparators:* (standard group—silymarin, toxic group—antitubercular medicines, and treatment group—plant extracts)
• *Animal models*: Studies on Wistar male or female rats and rodent models of antitubercular drug‐induced hepatotoxicity were considered.
• *Dosage range*: The dose range for antitubercular medicines was considered between 25 and 200 mg/kg, followed by the range for medicinal plants between 50 and 1000 mg/kg.
• *Outcome measures:* The studies that analyzed liver function, including aspartate aminotransferase (AST) in U/L, alanine transaminase (ALT) in U/L, alkaline phosphatase (ALP) in U/L, total bilirubin (TBil) in mg/dL, and antioxidant enzyme parameters, including GSH in mM/mg, catalase (CAT) in U/mg, and superoxide dismutase (SOD) in U/mg, were included (Table 1).

**TABLE 1:** Outcome parameters: the normal values and values indicating hepatotoxicity [10–17].

Outcome parameters	Normal range (human)	Toxicity range (human)	Normal range (rats)	Toxicity range (rats)	Reason
AST	5–30 U/L	Elevated (> 2–3*x* normal)	82.5–230.75 U/L	Elevated (> 2–3*x* fold)	AST is an enzyme found in the muscles and the liver. The anti‐TB drugs, such as isoniazid and rifampicin, cause hepatotoxicity, which causes injury to the liver cells, or hepatocytes, allowing the enzymes to penetrate the bloodstream. A raised AST level indicates damage to the liver cells, particularly damage to the mitochondria, since AST is also found in the mitochondria.
ALT	4–36 U/L	Elevated (> 2–3*x* normal)	17.79–50.55 U/L	Elevated (> 2–3*x* fold)	ALT is more liver‐specific than AST. It is released into the bloodstream as a result of hepatocyte damage caused by toxic compounds formed during the metabolism of anti‐TB medications, particularly isoniazid. Elevated ALT levels can directly detect hepatocellular damage.
ALP	30–120 U/L	Mildly elevated	70.89–437.41 U/L	Mildly elevated	Bilateral ducts are connected to ALP. ALP may slightly increase due to cholestasis, a condition caused by anti‐TB medications. Rather than indicating hepatocyte injury, the rise in ALP prompts either damage/blockage in the bile duct.
TBil	0.1–1.2 mg/dL	Elevated (> 2 mg/dL)	0.02–0.31	Elevated (> 2 mg/dL)	One waste product of hemoglobin breakdown that is often handled by the liver is bilirubin. Hyperbilirubinemia results from a decrease in the liver’s ability to eliminate bilirubin in drug‐induced liver injury (DILI). A hepatocellular injury or cholestasis may be the cause of the liver’s impaired excretory function, which is indicated by elevated total bilirubin levels.
GSH	1–6 μM (blood plasma)	Decrease	5 8 μmol/g tissue	Decrease	Glutathione is an essential antioxidant that counteracts free radicals to shield liver cells from oxidative damage. Anti‐TB medications produce more reactive oxygen species (ROS), which overpowers the liver’s antioxidant capacity and depletes glutathione. This increases the liver’s vulnerability to oxidative stress and damage.
CAT	50–150 U/mg	Decrease	40–70 U/mg	Decrease	Hydrogen peroxide is a reactive oxygen species that can be neutralized by the enzyme catalase. Overproduction of ROS from the metabolism of medications such as isoniazid causes catalase activity to be depleted during anti‐TB drug‐induced hepatotoxicity. The liver’s capacity to detoxify ROS is compromised by decreased catalase, which exacerbates oxidative damage.
SOD	110–200 U/mg or U/mL	Decrease	8–15 U/mg	Decrease	Another antioxidant enzyme that changes superoxide radicals into less harmful compounds like hydrogen peroxide is called superoxide dismutase (SOD). When anti‐TB drugs cause hepatotoxicity, the SOD system is less active because of the excessive amount of ROS produced. Reduced SOD activity is indicative of an imbalance between antioxidant defense and ROS generation, which can cause oxidative stress and damage to the liver.

#### 2.3.2. Exclusion Criteria

The criteria used to exclude studies are as follows:

• In vitro studies without in vivo validation
• Studies lacking appropriate controls
• Case reports, reviews, conference abstracts, and editorials
• Studies not reporting quantitative outcome data suitable for meta‐analysis
• Studies investigating hepatoprotection against toxins other than anti‐TB medications
• Duplicate publications or overlapping datasets

### 2.4. Data Extraction

A data extraction form was used to extract information systematically from research publications. Full‐text articles from the databases listed above were downloaded. The retrieved data were loaded with the following titles into the Microsoft Excel Software: study ID, plant part, state/country, anti‐TB medicines, standard group, toxic group, toxic dose concentrations, number of days, and different parameters such as AST, ALT, ALP, TB, GSH, CAT, and SOD (Supporting table no. 1).

## 3. Results

### 3.1. Selection of Included Studies

Out of the 565 articles identified through the mentioned search databases, 327 were removed owing to duplication. After examining the titles and abstracts of 238 papers, 156 were excluded for lack of relevance. After reviewing 82 full‐text papers, 68 of them were chosen based on pre‐established inclusion and exclusion criteria [5, 9, 10, 18–80]. The screened research articles provided sufficient evidence of the effect of herbal extracts on antitubercular drug‐induced hepatotoxicity across the chosen parameters. Several studies included data on various animal models, enzyme parameters, anti‐TB medications, and doses. The entire screening process is represented using a PRISMA flowchart (Figure 1)*.*

**FIGURE 1:**
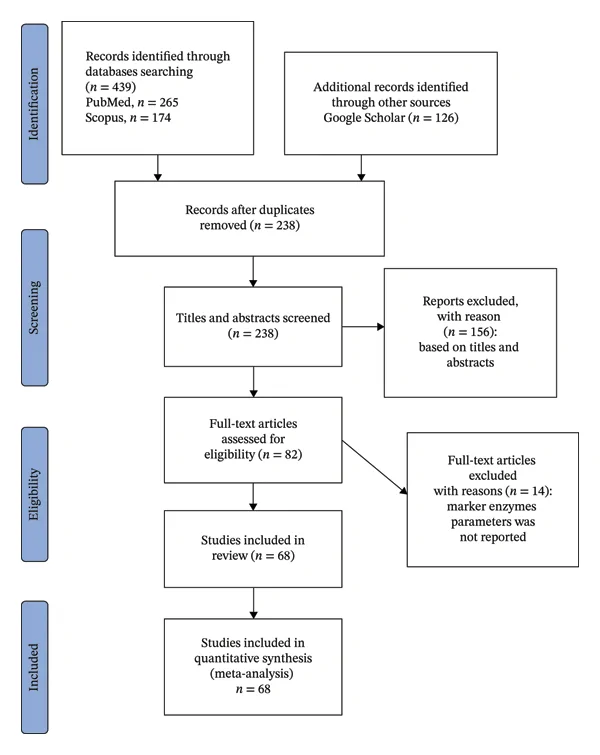
PRISMA flowchart showing the selection process for relevant research articles.

The included studies were analyzed for the effect of the plant extracts on the liver enzymes and their antioxidant properties, details of which are narrated below:

*AST:* It is observed from the analysis of the AST levels from all selected studies that there is significant variation in hepatoprotective activities among plant extracts (Table 2). Among all the plant extracts included in the studies, it is observed that the extracts from *Luffa acutangula* L.*, Cucumis trigonus* Rox., and *Ocimum canum* Sims. showed the highest hepatoprotective activity, with > 75% reduction in AST levels compared to the toxic control group (Table 3). *Lawsonia inermis* L., *Asparagus racemosus* W., *Centella asiatica* L., *Allium sativum* L., *Persea americana* Mill., and *Telfairia occidentalis* Hook*.*, and *Saccharum officinarum* L. extracts show significant hepatoprotection, as indicated by a 60%–75% reduction in AST level (Table 3). Moderate hepatoprotection, indicated by a 40%–60% reduction in AST level, is shown by various plants such as *Annona squamosa* L., *Azadirachta indica* L., *Crocus sativus* L., *Leucas cephalotes* R., *Polygonatum cirrhifolium* W., *Mirabilis jalapa* L., *Glinus lotoides* L., *Ficus religiosa* L., *Bacopa monnieri* L., *Pongamia pinnata* L., *Telfairia occidentalis* Hook*., Euphorbia fusiformis* B., *Carica papaya* L., *Ensete ventricosum* Welw.*, Sesbania grandiflora* L., *Amaranthus graecizans* L., *Citrus sinensis* L., and *Calotropis procera* Aiton (Table 3). However, it is also observed that a few of these studies showed minimal or inadequate hepatoprotection, as indicated by a lack of significant reduction in AST levels or even showed higher AST levels in comparison to those of the toxic group (Table 3).

**TABLE 2:** Effect of plant extracts on the AST levels compared with toxic group (anti‐TB drugs) and silymarin (standard).

Plant scientific name	Authors	Anti‐TB drugs	Toxic group (anti‐TB drugs) mg/kg		Standard group (silymarin) mg/kg		Treatment group (PE) mg/kg		% protection by standard (silymarin) compared to the toxic group	% protection by treatment group (PE) compared to the toxic group (^∗^mean values are for the highest conc. of PE)	% protection by treatment group (PE) compared to the standard (silymarin)
			Conc. of drugs mg/kg	Mean mg/kg bw	Conc. of standard (silymarin) mg/kg	Mean mg/kg bw	Conc. of PE mg/kg	Mean mg/kg bw			
*Abelmoschus ficulneus* L.	Urugonda and Vanapatla [9]	INH and RIF	50	85.64	100	54.13	100,200, 400	73.51	36.79	14.16^∗^	−35.80
*Adenanthera pavonina* L.	Mujahid et al. [19]	INH and RIF	50	146.41	100	81.17	100 and 200	90.13	44.56	38.44	−11.04
*Allium cepa* L.	Sagar et al. [20]	INH and RIF	50	396.71	100	428.2	100	495.01	−7.94	−24.78	−15.60
*Allium sativum* L. and milk thistle	Ilyas et al. [21]	INH	50	272.7	200	127.4	250	98.5	53.28	63.88	22.68
*Annona squamosa* L.	Uduman et al. [8]	INH and RIF	100	368.72	2.5	147.8	250 and 500	164.84	59.92	55.29	−11.53
*Persea americana* Mill. and *Telfairia occidentalis* Hook*. f.*	Jibril et al. [22]	INH, RIF, PZA, and EMB	51.4	47	NA	NA	125 and 200	17	0.00	63.83	NA
*Bacopa monnieri* L.	Evan et al. [24]	INH and RIF	50	185	25	96	500	98	100.00	52.17	NA
*Carica papaya* L.	Pandit et al. [27]	INH and RIF	125	151.6	NA	138.9	100 and 200	175.3	48.11	47.03	−2.08
*Cassia auriculata* L.	Jaydeokar et al. [28]	INH, RIF, and PZA	54	308.4	25	168.33	400	208.08	100.00	40.24	NA
*Centella asiatica* L.	Ghosh et al. [29]	INH	50	262.53	100	18.32	300 and 600	16.88	100.00	33.77	NA
*Cissampelos pareira* L.	Sangameswaran et al. [30]	INH and RIF	50	50.62	50	168.36	20, 40, 60, and 100	254.32	54.96	43.16	−26.21
*Cissus quadrangularis* L.	Swamy et al. [31]	RIF	54	376.4	100	69.98	500	75.68	35.88	20.74	−23.61
*Cnidoscolus chayamansa* McVaugh.	Pérez‐González et al. [32]	INH, RIF, and PZA	50 and 100	90.84	50	308.5	50, 50, and 100	302.7	63.81	66.65	7.86
*Alpinia officinarum* H.	Eram et al. [36]	INH and RIF	50	92.88	100	50.18	200 and 400	74.18	55.27	32.43	−51.06
*Hedyotis corymbosa* L.	Lina et al. [38]	INH and RIF	50	78	50	31.15	500	52.33	22.96	16.69	−8.15
*Hibiscus vitifolius* L.	Samuel et al. [10]	INH, RIF, and PZA	7.5, 10, and 35	462.78	100	73.31	400	371.42	6.43	8.19	1.88
*Ipomoea carnea* J.	Suresh et al. [39]	INH, RIF, and PZA	7.5, 10, and 35	172.28	100	89.21	125,250, and 500	141.2	70.65	11.86	−200.32
*Ixora pavetta* A.	Reddy et al. [40]	INH and RIF	100	324	2.5	196	100 and 400	292	59.32	42.75	−40.74
*Jasminum grandiflorum* L.	Dhamal et al. [41]	INH	54	323.5	50	244.2	200	271.2	100.00	30.80	NA
*Lawsonia inermis* L.	Darvin et al. [42]	INH and RIF	50 and 100	492.4	100	112.8	50 and 100	138.9	45.97	20.13	−47.83
*Nyctanthes arbor-tristis* L.	Chaudhary et al. [45]	INH, RIF and PZA	7.5, 10 and 35	242.64	100	138.32	250 and 500	221.1	100.00	22.91	NA
*Ocimum canum* Sims.	Radhika et al. [46]	INH	54	201.7	50	32.25	200 and 300	43.9	100.00	60.00	NA
*Polygonatum cirrhifolium* W.	Ghaia et al. [48]	INH and RIF	50	80.27	50	24.21	200 and 400	41.39	60.06	32.91	−67.99
*Solanum nigrum* L.	Fyed et al. [49]	INH and RIF	50	179.27	50	162.78	250, 500, and 1000	159.8	84.16	19.74	−406.64
*Tamarindus indica* L.	Amir et al. [50]	INH and RIF	50 and 100	113.56	50	58.2	250 and 500	100.08	48.22	18.04	−58.28
*Telfairia occidentalis*	Nwidu and Obima [51]	INH and RIF	100	124	50	75	125, 250, and 500	76.3	39.51	9.88	−48.98
*Vernonia* *amygdalina* Del.	Tilaye et al. [53]	INH and RIF	75	125.3	100	114.4	200 and 400	115	24.51	16.17	−11.06
*Ziziphus* *oenoplia* L.	Rao et al. [55]	INH and RIF	50	131.34	100	82.42	150 and 300	104.12	77.09	71.79	−23.14
*Acanthospermum hispidum* DC.	Himaja [56]	INH, RIF, PZA and EMB	27,40,66 and53	171.8	100	83.92	400	112.6	100.00	16.89	NA
*Amaranthus graecizans* L.	Ishtiaq et al. [57]	INH and RIF	50 and 100	45.2	100	40.6	400	26.4	100.00	34.56	NA
*Crocus sativus* L.	Wali et al. [61]	INH and RIF	50	131.34	2.5	95.01	100, 250, and 500	145.83	42.99	8.88	−59.85
*Cucumis trigonus* Roxb.	Gopalakrishnan et al. [62]	INH and RIF	50	144.51	5 mL	32.63	200	29.11	84.01	78.24	−36.12
*Embelia tsjeriam-cottam* A.DC.	Sambrekar et al. [63]	INH	50	83.33	100	56.5	125, 250, and 500	73.5	NA	25.26	NA
Euphorbia fusiformis B.	Anusuya et al. [64]	RIF	1000	247.5	10	139.17	100, 200, and 300	140.67	69.84	48.44	−70.96
*Ficus benghalensis* L.	Parameswari et al. [65]	INH and RIF	100	239	10	170	100, 200, and 300	158	9.20	10.86	1.83
*Ficus religiosa* L.	Parameswari et al. [66]	INH and RIF	100	584	100	351	200 and 400	309	48.75	11.87	−71.96
*Glinus lotoides* L.	Rameshwari and Arockia Selvi et al. [67]	RIF	100	103	25	45	100,300 and 700	54	39.52	38.47	−1.73
*Curcuma longa* L. (Haridra Ghrit)	Vyavahare et al. [68]	INH, RIF, and PZA	100,300, and 700	282.07	200	153.83	200 and 400	163.34	8.70	8.22	−0.52
*Leucas cephalotes* R.	Bais and Saiju [69]	INH and RIF	100	136.44	200	61.35	100, 200, and 400	69.32	100.00	−8.27	NA
*Luffa acutangula* L.	Jadhav et al. [70]	RIF	100	114	100	36.4	250 and 500	21	37.25	20.72^∗^	−26.33
*Mirabilis* jalapa L.	Jyothi et al. [71]	INH, RIF, PZA, and EMB	27, 40, 66, and 53	386.5	100	166.67	150 and 300	201.67	51.15	34.46	−34.18
*Monotheca buxifolia* F.	Ullah et al. [72]	INH and RIF	50	131	200	85.5	150, 200, and 250	91.3	10.18	41.59	34.98
*Moringa oleifera* L.	Pari and Kumar [73]	INH, RIF, and PZA	7.5, 10, and 35	105.22	2.5	87.11	100,200 and 400	78.37	100.00	71.05	NA
*Mucuna pruriens* L.	Obogwu et al. [5]	INH and RIF	100	109	50	66.1	100, 250, and 500	68.82	100.00	23.04	NA
*Phyllanthus debilis* K.	Koppal et al. [75]	INH, RIF, and PZA	50, 100, and 350	347.08	50	145.32	200 and 400	291.51	100.00	41.38	NA
*Pongamia pinnata* L.	Derangula et al. [76]	INH and RIF	27 and 40	390.5	100	168.78	200 and 400	211.67	68.58	51.78	−53.49
*Sesbania grandiflora* L.	Kuttiappan et al. [79]	INH, RIF, and PZA	0.5	124.5	100	51.5	250 and 500	72.5	77.42	79.86	10.79

**TABLE 3:** Degree of hepatoprotective efficacy of medicinal plants based on restoration of liver function and antioxidant parameters (^∗^Table 2 and supporting table nos. 2–7).

Study parameters	Plants showing hepatoprotective effect		
	> 75%	60%–75%	40%–60%
AST	*Luffa acutangula* L., *Cucumis trigonus* L., and *Ocimum canum* Sims	*Lawsonia inermis* L., *Asparagus racemosus* W., *Centella asiatica* L., *Allium sativum* L., *Persea americana* Mill*., Telfairia occidentalis* Hook., and *Saccharum officinarum* L.	Of the 65 included studies, 21 plants showed modest hepatoprotective efficacy against AST elevation^∗^.
ALT	*Cucumis trigonus* L., *Luffa acutangula* L., *Asparagus racemosus* W., *Ocimum sanctum* L., *Allium sativum* L., *Persea americana* Mill*.,* and *Telfairia occidentalis* Hook., and *Glinus lotoides L.*	*Lawsonia inermis* L., *Centella asiatica* L., *Citrus sinensis* L., *Mirabilis jalapa* L., *Pongamia pinnata* L., *Saccharum officinarum* L., *Calotropis procera* Aiton., and *Acanthospermum hispidum* DC*.*	Of the 65 studies included, 14 plants indicated modest hepatoprotective efficacy against ALT elevation^∗^.
ALP	*Luffa acutangula* L.	*Ocimum sanctum* L., *Allium sativum* L., *Asparagus racemosus* W., *Lawsonia inermis* L., *Persea americana* Mill*.*, and *Telfairia occidentalis* Hook., *Mucuna pruriens* L., and *Amaranthus graecizans* L.	Of the 56 studies included, 17 plants indicated modest hepatoprotective efficacy against ALP elevation^∗^.
TBil	*Euphorbia fusiformis* B., *Cucumis trigonus* L., *Lawsonia inermis* L., and *Mirabilis jalapa* L.	*Embelia tsjeriam-cottam* A.DC.*, Pongamia pinnata* L., *Garcinia kola* H., *Acanthospermum hispidum* DC*., Sesbania grandiflora* L., *Curcuma longa in ghee* L. (*Haridra Ghrit*), and *Bacopa monnieri* L.	Of the 47 included studies, 10 plants showed modest hepatoprotective efficacy against TBil elevation^∗^.
GSH	*Garcinia kola* H., *Leucas cephalotes* R., *Trapa natans* L., *Ficus benghalensis* L., *Abelmoschus ficulneus* L., *Luffa acutangula* L., *Cissus quadrangularis* L.	*Mucuna pruriens* L. and *Embelia tsjeriam-cottam* A.DC.	*Pongamia pinnata* L., *Centella asiatica* L., *Mirabilis jalapa* L., *Nyctanthes arbor-tristis* L., *Cucumis trigonus* L., and *Alpinia officinarum* L.
CAT	*Maytenus royleanus* W., *Annona squamosa* L., *Embelia tsjeriam-cottam* A.DC.*, Luffa acutangula* L., *Abelmoschus ficulneus* L., *Trapa natans* L.	*Alpinia officinarum* L., *Garcinia kola* H., and *Cucumis trigonus* L.	*Mirabilis jalapa* L. and *Cnidoscolus chayamansa* L.
SOD	*Alpinia officinarum* H., *Cassia auriculata* L., *Azadirachta indica* L., *Centella asiatica* L., *Garcinia kola* H., *Punica granatum* L., *Mucuna pruriens* L., *Embelia tsjeriam-cottam* A.DC., and *Cucumis trigonus* L.	*Cissus quadrangularis* L. and *Leucas cephalotes* L.	*Adenanthera pavonina* L., *Ocimum canum* Sims*, Sesbania grandiflora* L., *Nyctanthes arbor-tristis* L., *Trapa natans* L., and *Abelmoschus ficulneus* L.

Some of the plant extracts, such as *Luffa acutangula* L. (400 mg), *Amaranthus graecizans* L. (400 mg), and *Allium sativum* L. (300 mg), have significantly outperformed silymarin (100–200 mg) in lowering the AST levels. However, it should be noted that these results are mostly derived from standalone research studies, and the concentrations of the plant extracts used are higher than those of the standard (silymarin). Though silymarin used in these studies is a pure compound, the plant extracts are concoctions of many phytochemicals. Therefore, we predict that the aforementioned plant extracts could be a potential source of hepatoprotective bioactive molecules.

*ALT:* It is observed from the analysis of the ALT levels from all selected studies that there is significant variation in hepatoprotective activities among plant extracts. The highest reductions in ALT levels (> 75%) were reported for extracts of *Cucumis trigonus* Roxb.*, Luffa acutangula* L., *Asparagus racemosus* W., *Ocimum sanctum* L., *Allium sativum* L., *Persea americana* Mill., and *Telfairia occidentalis* Hook., and *Glinus lotoides* L., indicating strong hepatoprotective potential compared to the toxic (Table 3). A significant hepatoprotective effect (60%–75% reduction) was observed with extracts from *Centella asiatica* L., *Citrus sinensis* L., *Mirabilis jalapa* L., *Pongamia pinnata* L., *Saccharum officinarum* L., *Calotropis procera* A., and *Acanthospermum hispidum* DC (Table 3). Extracts from several other plants, including *Bacopa monnieri* L., *Ficus benghalensis* L., *Monotheca buxifolia* F., *Garcinia kola* H., *Embelia tsjeriam-cottam* A.DC., *Allium sativum* L., *Ficus religiosa* L., *Telfairia occidentalis* Hook*., Amaranthus graecizans* L., *Cassia auriculata* L., *Annona squamosa* L., *Telfairia occidentalis* Hook*., Adenanthera pavonine* L., *Azadirachta indica* L., and *Hedyotis corymbosa* L., demonstrated moderate hepatoprotective activity (40%–60% reduction in ALT levels) (Table 3). However, it is also observed that a few of these studies showed minimal or inadequate hepatoprotection, as indicated by a lack of significant reduction in ALT levels, or even showed higher ALT levels in comparison to those of the toxic group (Supporting table no. 2).

*ALP:* It is observed from the analysis of the ALP levels from all selected studies that there is significant variation in hepatoprotective activities among plant extracts. The highest reduction (> 75%) in ALP levels was reported for the extract from *Luffa acutangula* L., demonstrating pronounced hepatoprotective potential. A significant protective effect (60%–75% reduction) was observed for extracts from *Ocimum sanctum* L., *Allium sativum* L., *Asparagus racemosus* W., *Lawsonia inermis* L., *Persea americana* Mill., and *Telfairia occidentalis* Hook.*, Mucuna pruriens* L., and *Amaranthus graecizans* L. (Table 3). Several other extracts from other plant species, *Allium sativum* L., *Curcuma longa* L. (Haridra Ghrit), *Glinus lotoides* L., *Garcinia kola* H., *Pongamia pinnata* L., *Mirabilis jalapa* L., *Crocus sativus* L., *Bacopa monnieri* L., *Moringa oleifera* L., *Calotropis procera* A., *Punica granatum* L., *Vitex negundo* L., *Azadirachta indica* L., *Cucumis trigonus* Roxb*., Centella asiatica* L., *Carica papaya* L., and *Leucas cephalotes* R., demonstrated moderate hepatoprotective activity (40%–60% reduction in ALP levels), including Table 3. In contrast, some studies reported ALP reductions below 40%, indicating limited hepatoprotective efficacy. Overall, most plant extracts demonstrated moderate protective effects comparable to the reference hepatoprotective agent silymarin (Supporting table no. 3)*.*

In addition to the liver enzymes, the study also examined antioxidant markers like TBil, GSH, CAT, and SOD.

*TBil:* It is a measure of liver dysfunction that was lowered considerably upon treatment with most plant extracts. TBil showed substantial improvement following treatment with several plant extracts across the included studies. Extracts from *Euphorbia fusiformis* B., *Cucumis trigonus* Roxb*., Lawsonia inermis* L., and *Mirabilis jalapa* L. showed the greatest significant reductions (> 75%) in TBil levels compared to the toxic control group (Table 3). Extracts from *Embelia tsjeriam-cottam* A.DC., *Pongamia pinnata* L., *Garcinia kola* H., *Acanthospermum hispidum* DC., *Sesbania grandiflora* L., *Curcuma longa* L. (*Haridra Ghrit*), and *Bacopa monnieri* L. showed a significant decrease in bilirubin levels (60%–75%), indicating hepatoprotective potential (Table 3). Additionally, moderate reductions (40%–60%) were observed for extracts from *Abelmoschus ficulneus* L., *Amaranthus graecizans* L., *Hedyotis corymbosa* L., *Leucas cephalotes* R., *Telfairia occidentalis* Hook*., Carica papaya* L., *Moringa oleifera* L., *Allium sativum* L., *Ficus benghalensis* L., and *Monotheca buxifolia* F. when compared to the toxic group (Table 3). Collectively, these results show that, while certain plant extracts significantly reduced TBil levels, the majority showed moderate hepatoprotective effects compared to silymarin (Supporting table no. 4).

*GSH:* GSH is a major intracellular antioxidant and is crucial in protecting the liver from oxidative stress. GSH levels provide insight into the antioxidant restorative potential of the plant extracts tested against the toxic group. Several extracts showed a significant increase in intracellular GSH levels, indicating effective oxidative stress reduction. *Garcinia kola* H., *Leucas cephalotes* R., *Trapa natans* Roxb*., Ficus benghalensis* L., *Abelmoschus ficulneus* L., *Luffa acutangula* L., and *Cissus quadrangularis* L. extracts exhibited the highest rate of restoration (> 75%), indicating a considerable improvement of cellular antioxidant defense (Table 3). *Mucuna pruriens* and *Embelia tsjeriam-cottam* A.DC. showed an increase in GSH levels (60%–75%), indicating important antioxidative action. Additionally, *Pongamia pinnata* L., *Centella asiatica* L., *Mirabilis jalapa* L., *Nyctanthes arbor-tristis* L., *Cucumis trigonus* Roxb., and *Alpinia officinarum* H*.* all showed moderate GSH restoration (40%–60%) (Table 3). Overall, while certain plant extracts significantly increased GSH levels, some showed moderate recovery of antioxidant capacity, which was generally comparable to the effect of the reference hepatoprotective drug silymarin (Supporting table no. 5).

*CAT*: CAT activity demonstrated a clear enhancement in several plant‐treated groups under toxic conditions, reflecting improved antioxidant defense mechanisms. The most pronounced elevation in CAT activity (> 75%) was observed with *Maytenus royleanus* W., *Annona squamosa* L., *Embelia tsjeriam-cottam* A.DC.*, Luffa acutangula* L., *Abelmoschus ficulneus* L., and *Trapa natans* Roxb*.*, indicating strong restoration of endogenous antioxidant capacity (Table 3). A considerable increase (60%–75%) in CAT activity was reported *for Alpinia officinarum* H., *Garcinia kola* H., and *Cucumis trigonus* L., suggesting notable antioxidative effects (Table 3). In addition, moderate enhancement (40%–60%) was documented for *Mirabilis jalapa L*. and *Cnidoscolus chayamansa* McVaugh (Table 3). Overall, these outcomes suggest that most plant extracts increased the CAT levels significantly, while a few plant extracts provided mild antioxidant restoration that was broadly comparable to the reference hepatoprotective drug silymarin (Supporting table no. 6).

SOD: SOD activity recovered significantly in several plant‐treated groups across the included experiments, indicating an increase in enzymatic antioxidant defense. The most pronounced enhancement (> 75%) in SOD activity was observed in *Alpinia officinarum* H., *Cassia auriculata* L., *Azadirachta indica* L., *Centella asiatica* L., *Garcinia kola* H., *Punica granatum* L., *Mucuna pruriens* L., *Embelia tsjeriam-cottam* A.DC., and *Cucumis trigonus* Roxb*.*, indicating strong restoration of antioxidant enzyme activity (Table 3). A substantial increase (60%–70%) was reported for *Cissus quadrangularis* L. and *Leucas cephalotes* R., suggesting considerable antioxidative potential (Table 3). Additionally, moderate increases in SOD activity (40%–60%) were observed in *Maytenus royleanus* W., *Adenanthera pavonina* L., *Ocimum canum* Sims., *Sesbania grandiflora* L., *Nyctanthes arbor-tristis* L., *Trapa natans* H., and *Abelmoschus ficulneus* L., indicating partial antioxidant enzyme activity restoration under toxic conditions (Table 3). *Cnidoscolus chayamansa* McVaugh, on the other hand, had a rather minor impact on SOD modulation. Overall, the majority of the evaluated plant extracts showed improvements in SOD levels that were generally comparable to the antioxidant response observed with the standard hepatoprotective agent silymarin, supporting their potential role in mitigating oxidative stress‐associated hepatic injury (Supporting table no. 7).

### 3.2. Quality Assessment

Quality assessments were performed using SYRCLE’s scale, which ensures that the included animal studies are reliable, unbiased, and well‐designed (Supporting table no. 8). Most of the key methodological details, such as random sequence generation, allocation concealment, and blinding, were frequently unclear or not reported. This indicates a moderate risk of bias and highlights the need for improved transparency in study design. Overall, while the data are generally reliable, future studies should enhance methodological reporting to strengthen the validity of their findings.

## 4. Discussion

In this systematic review, preclinical data were employed to evaluate the hepatoprotective activity of medicinal plants against liver injury caused by antitubercular drugs, commonly known as DILI. Among the selected 68 studies on medicinal plants, some exhibited better hepatoprotective activity upon biochemical and antioxidant marker‐based assessments. Notably, *Luffa acutangula* L., *Lawsonia inermis* L., and *Cucumis trigonus* L. consistently ranked the highest in hepatoprotective activity by exhibiting reduction in the AST, ALT, ALP, and TBil profiles (Table 3). Extracts from plants such as *Garcinia kola* H., *Trapa natans* L., *Abelmoschus ficulneus* L., and *Embelia tsjeriam-cottam* A.DC. exhibited enhanced antioxidant activity by increasing the GSH, CAT, and SOD levels (Table 3). Interestingly, *Luffa acutangula* L. and *Cucumis trigonus* L. were helpful in the reduction of the AST, ALT, ALP, and TBil alongside their antioxidant activity.

A few plant extracts showed moderate hepatoprotective activity, which may vary due to various reasons such as differences in phytochemical content, extraction methods, experimental models, and dosages. The hepatoprotective activity of these promising plant species may be attributed to various phytochemicals such as flavonoids, phenolic derivatives, terpenoids, and sulfur‐containing compounds, which are known to affect oxidative stress, inflammatory pathways, and detoxification processes in the liver [81].

Oxidative stress is considered one of the major mechanisms underlying anti‐TB drug‐induced hepatotoxicity. First‐line anti‐TB drugs generate ROS and reactive metabolites that promote lipid peroxidation, mitochondrial dysfunction, depletion of endogenous antioxidant defenses, and hepatocellular injury. Many plants included in the present review demonstrated restoration of antioxidant enzymes such as SOD, CAT, and GSH, along with a reduction in lipid peroxidation markers, suggesting that modulation of oxidative stress pathways may be a key mechanism responsible for their hepatoprotective activity. In addition, phytochemicals such as flavonoids and phenolic compounds are known to exert anti‐inflammatory and free radical scavenging effects that may further contribute to hepatocellular protection [4, 5, 81–84].

Several plant extracts outperformed silymarin (standard) across key hepatotoxicity markers in preclinical studies, as shown in Table 4. However, these findings largely stem from single studies, with extract doses often exceeding those of silymarin, a pure compound, while the extracts represent complex mixtures of phytochemicals. Thus, the above‐mentioned extracts warrant further investigation as promising sources of hepatoprotective bioactive molecules.

**TABLE 4:** Extracts from medicinal plants exhibiting hepatoprotective activity superior to silymarin across biochemical and antioxidant parameters.

Study outcome	Conc. of silymarin used in the studies	Extracts from medicinal plants that outperformed silymarin (standard) in hepatoprotective activity (> 20% better activity than silymarin)	Reference table number
AST	100–200 mg	*Luffa acutangula* L. (400 mg), *Amaranthus graecizans* L. (400 mg), and *Allium sativum* L. (300 mg)	Table 3
ALT	2.5–200 mg	*Lawsonia inermis* L. (100 mg), *Ficus religiosa* L. (300 mg), *Amaranthus graecizans* L. (400 mg), *Cucumis trigonus* L. (500 mg), *Telfairia occidentalis* Hook. (500 mg), and *Allium sativum* L. (250 mg)	Supporting table no. 2
ALP	200 mg	*Moringa oleifera* L. (250 mg) and *Luffa acutangula* L. (400 mg)	Supporting table no. 3
TBil	2.5–200 mg	*Euphorbia fusiformis* B. (500 mg), *Mucuna pruriens* L. (400 mg), *Ficus benghalensis* L. (300 mg), *Cucumis trigonus* L. (500 mg), and *Moringa oleifera* L. (250 mg)	Supporting table no. 4
GSH	2.5–200 mg	*Pongamia pinnata* L. (400 mg), *Mirabilis jalapa* L. (500 mg), *Carica papaya* L. (400 mg), *Tamarindus indica* L. (500 mg), *Abelmoschus ficulneus* L. (400 mg), *Cucumis trigonus* L. (500 mg), *Nyctanthes arbor-tristis* L. (500 mg), *Alpinia officinarum* L. (400 mg), *Luffa acutangula L.* (400 mg), and *Trapa natans L*. (400 mg)	Supporting table no. 5
CAT	2.5–100 mg	*Tamarindus indica* L. (500 mg), *Abelmoschus ficulneus* L. (400 mg), *Hibiscus vitifolius* (400 mg), *Cucumis trigonus* L. (500 mg), *Nyctanthes arbor-tristis* L. (500 mg), *Mirabilis jalapa* L. (500 mg), *Trapa natans* L. (400 mg), *Alpinia officinarum* L. (400 mg), and *Ziziphus oenoplia* L. (300 mg)	Supporting table no. 6
SOD	2.5–200 mg	*Hibiscus vitifolius* L. (400 mg), *Tamarindus indica* L. (500 mg), *Adenanthera pavonina* L. (200 mg), *Mirabilis jalapa* L. (500 mg), *Nyctanthes arbor-tristis* L. (500 mg), *Cucumis trigonus* L. (500 mg), *Cnidoscolus chayamansa*McVaugh. (100 mg), *Abelmoschus ficulneus* L. (400 mg), *Sesbania grandiflora* L. (500 mg), *Trapa natans* L. (400 mg), and *Alpinia officinarum L.* (400 mg)	Supporting table no. 7

A recent systematic review and network meta‐analysis that included 14 randomized controlled trials involving 3423 human patients demonstrated that a combination of *Curcuma longa* (turmeric) and *Tinospora cordifolia*, as well as N‐acetylcysteine (NAC), significantly reduced the incidence of anti‐TB DILI compared to the placebo; Additionally, polyherbal formulations were found to significantly lower serum ALP and TBil levels [85]. Several in vivo studies have highlighted the hepatoprotective properties of individual plant species. For instance, *Silybum marianum* (milk thistle) has shown significant protective effects primarily through its active compound, silymarin, which stabilizes hepatic cell membranes, reduces lipid peroxidation, and promotes liver regeneration [86]. Polyherbal preparations such as Liv52 are also commonly used hepatoprotective drugs that exert their activity against toxin‐induced liver damage via antioxidant and membrane‐stabilizing actions [87, 88].

Despite the growing popularity of herbal hepatoprotective supplements, research articles that used several widely recognized medicinal plants, such as *Phyllanthus niruri* and *Andrographis paniculata,* were not included in the present analysis due to the predefined inclusion criteria and the selected publication period (2002–2024) [89]. However, both of these plants are well known for their hepatoprotective potential and have been explored for their ability to prevent antitubercular DILI [89]. The study showed the hepatoprotective potential of *Phyllanthus niruri* and *Andrographis paniculata* in INH‐ and RIF‐induced hepatotoxicity in rats and demonstrated that extracts from both plants significantly reduced elevated liver enzyme levels and increased antioxidant defense mechanisms, protecting the liver from damage caused by antitubercular drugs [89]. This potential of *Phyllanthus niruri* and *Andrographis paniculata* was largely attributed to their antioxidant and anti‐inflammatory properties, which are known to mitigate oxidative stress and liver injury caused by antitubercular drugs [89].

The current systematic review has several limitations that should be acknowledged. Although this review consolidates the available preclinical evidence on plant extracts for managing anti‐TB DILI, most results are supported by only a single experimental study, limiting the strength of the conclusions and highlighting the need for independent replication. Methodological inconsistencies further challenge the internal validity of the findings. One notable observation is the inconsistency in outcome parameters across the selected studies, which exhibit substantial variability. Each study employed different experimental conditions, including anti‐TB drug concentrations, durations of animal experimentation, assay kits, silymarin as the standard at different concentrations, extraction solvents for plant extracts, and the final concentrations of the plant extracts. Moreover, the absence of baseline values for control animals (untreated with anti‐TB drugs) further compromises the reliability of these findings. However, it should also be noted that the percentage restoration may overestimate hepatoprotective efficacy in studies with extremely elevated toxic controls. Moreover, the translational gap between preclinical models and clinical practice remains a major barrier, restricting the direct applicability of these findings to human health interventions. Another important consideration is the variability among the included experimental models. Differences in animal species and strains, anti‐TB drug regimens, treatment duration, extraction procedures, dosage protocols, and outcome assessment methods may contribute to heterogeneity across studies and affect comparability of findings. Furthermore, preclinical animal models provide important mechanistic insights. The translation of these findings into clinical application remains challenging due to limited clinical studies, a lack of standardized herbal formulations, and insufficient long‐term safety evaluations.

### 4.1. Clinical and Research Implications

This systematic review evaluates preclinical evidence on medicinal plants’ hepatoprotective effects against anti‐TB DILI, identifying standout performers like *Luffa acutangula* L., *Lawsonia inermis* L., and *Cucumis trigonus* L. for reducing AST, ALT, ALP, and TBil, alongside others (e.g., *Trapa natans* L., *Abelmoschus ficulneus* L.), boosting GSH, CAT, and SOD beyond silymarin. These benefits likely stem from phytochemicals such as flavonoids and terpenoids targeting oxidative stress, with clinical parallels in *Curcuma longa*/*Tinospora cordifolia* and NAC trials [85]. The systematic identification of effective plant‐based interventions provides a basis for prioritizing further investigations into their mechanisms of action and potential integration with conventional anti‐TB therapy. Such research may facilitate the development of adjunctive therapies in the future that mitigate drug‐induced hepatotoxicity while preserving therapeutic efficacy.

## 5. Conclusions

The current systematic review provides a comprehensive synthesis of preclinical evidence regarding the hepatoprotective potential of medicinal plants against antitubercular DILI.

Medicinal plant extracts, particularly *Luffa acutangula* L., *Lawsonia inermis* L., and *Cucumis trigonus* L., demonstrated significant hepatoprotective activity in experimental models, showing improvements in hepatic biomarkers, antioxidant parameters, and histopathological outcomes comparable to or exceeding those of standard hepatoprotective agents such as silymarin. These protective effects are largely attributed to the presence of bioactive phytochemicals, including flavonoids, phenolics, terpenoids, and sulfur compounds, which may exert antioxidant, anti‐inflammatory, and cytoprotective effects.

The findings of this review support the growing therapeutic potential of plant‐based adjunctive interventions for the management of antitubercular drug‐induced hepatotoxicity. However, further studies are required to establish their translational applicability. Future research should focus on detailed mechanistic investigations, standardization of plant extracts, optimization of dosage regimens, long‐term toxicity evaluation, and well‐designed preclinical and clinical studies to validate safety and efficacy. Such advances may facilitate the development of effective adjunct therapies capable of reducing hepatotoxicity, improving treatment adherence, and enhancing clinical outcomes in TB management.

## Author Contributions

Surthi M. Ravedar, Kranthi Kiran Akula, and Kalesh M. Karun conceptualized the study. Surthi M. Ravedar, Kalesh M. Karun, Kranthi Kiran Akula, and Jyothi Bhat devised the methodology. Surthi M. Ravedar and Flemin Felix conducted data extraction. Surthi M. Ravedar, Kalesh M. Karun, and Flemin Felix performed data analysis. Subarna Roy, Kalesh M. Karun, Kranthi Kiran Akula, Jyothi Bhat, and Subarna Roy drafted the manuscript. Kalesh M. Karun, Kamran Zaman, and Kranthi Kiran Akula critically analyzed and revised the manuscript.

## Funding

The publication charges were met by the ICMR‐NITM’s institutional fund.

## Disclosure

All authors reviewed and approved the final manuscript.

## Conflicts of Interest

The authors declare no conflicts of interest.

## Supporting Information

Additional supporting information can be found online in the Supporting Information section.

## Supporting information


**Supporting Information** The supporting information provides extensive argumentation for this systematic review. Supporting table no.1 describes the primary study’s preliminary methodology, including plant parts, animal models, drug concentrations, study duration, biochemical and oxidative stress data, and references. Supporting table nos. 2–7 gives extensive comparison results for biochemical and oxidative markers, including ALT, ALP, TBil, GSH, CAT, and SOD. Supporting table no. 8 assesses the quality of included studies using the SYRCLE risk of bias tool. Collectively, these supplemental resources enhance the transparency, robustness, and interpretability of the findings presented in the primary text.

## Data Availability

Data supporting this systematic review can be obtained through the corresponding author upon request.

## References

[1] Huang Y. , Ai L. , Wang X. , Sun Z. , and Wang F. , Review and Updates on the Diagnosis of Tuberculosis, Journal of Clinical Medicine. (September 2022) 11, no. 19, 10.3390/jcm11195826. PMC9570811 36233689

[2] World Health Organization , WHO Global Lists of High Burden Countries for Tuberculosis (TB), TB/HIV and Multidrug/Rifampicin-Resistant TB (MDR/RR-TB), 2021.

[3] Maddahi S. Z. , Jokar A. , Kamalinejad M. , and Behnampur N. , The Efficacy of Jujube Syrup on the Prevention of Drug‐Induced Hepatotoxicity in Pulmonary Tuberculosis Patients: A Pilot Randomized Double‐Blind Placebo‐Controlled Clinical Trial, Pharmacology Research and Perspectives. (February 2022) 10, no. 1, 10.1002/prp2.902. PMC8929366 34939363

[4] Zodape G. V. , Effect of Aloe Vera Juice on the Hepatotoxicity Induced by Isoniazid Drug, Journal of Applied and Natural Science. (December 2011) 3, no. 2, 238–241, 10.31018/jans.v3i2.186.

[5] Obogwu M. B. , Akindele A. J. , and Adeyemi O. O. , Hepatoprotective and in Vivo Antioxidant Activities of the Hydroethanolic Leaf Extract of *Mucuna pruriens (Fabaceae)* in Antitubercular Drugs and Alcohol Models, Chinese Journal of Natural Medicines. (April 2014) 12, no. 4, 273–283, 10.1016/s1875-5364(14)60054-6. 24863352

[6] Luangchosiri C. , Thakkinstian A. , Chitphuk S. , Stitchantrakul W. , Petraksa S. , and Sobhonslidsuk A. , A Double-Blinded Randomized Controlled Trial of Silymarin for the Prevention of Antituberculosis Drug-Induced Liver Injury, BMC Complementary and Alternative Medicine. (Decemeber 2015) 15, 1–7, 10.1186/s12906-015-0861-7. PMC4580123 26400476

[7] Choijamts G. , Batsyren C. , Orkhon B. et al., Role of Liv. 52 DS Tablets as a Hepatoprotective Agent in Tuberculosis Patients Receiving Antitubercular Drugs: A Double Blind Placebo Controlled Study, ournal of Liver and Clinical Research. (2018) 5, 1042–1050.

[8] Uduman T. S. , Sundarapandian R. , Muthumanikkam A. et al., Protective Effect of Methanolic Extract of *Annona squamosa* Linn in Isoniazid-Rifampicin Induced Hepatotoxicity in Rats, Pakistan journal of pharmaceutical sciences. (April 2011) 24, no. 2, 129–134. 21454160

[9] Urugonda S. and Vanapatla S. R. , Hepatoprotective Effect of *Abelmoschus ficulneus* Root Extract Against Drug Induced Hepatoprotective Caused by the Combined Action of Isoniazid and Rifampicin, Journal of Advanced Scientific Research. (December 2021) 12, no. 04 Suppl 1, 163–170, 10.55218/jasr.s1202112417.

[10] Samuel A. J. , Mohan S. , Chellappan D. K. , Kalusalingam A. , and Ariamuthu S. , *Hibiscus vitifolius* (Linn.) Root Extracts Shows Potent Protective Action Against Anti-Tubercular Drug Induced Hepatotoxicity, Journal of Ethnopharmacology. (May 2012) 141, no. 1, 396–402, 10.1016/j.jep.2012.02.051. 22421378

[11] Liver test-Lala V. , Zubair M. , and Minter D. A. , Liver Function Tests, Statpearls, 2024, StatPearls Publishing, Treasure Island (FL), https://www.ncbi.nlm.nih.gov/books/NBK482489/. 29494096

[12] Saukkonen J. J. , Cohn D. L. , Jasmer R. M. et al., An Official ATS Statement: Hepatotoxicity of Antituberculosis Therapy, American Journal of Respiratory and Critical Care Medicine. (October 2006) 174, no. 8, 935–952, 10.1164/rccm.200510-1666st. 17021358

[13] Patel S. , Patel S. , Kotadiya A. et al., Age-Related Changes in Hematological and Biochemical Profiles of Wistar Rats, Laboratory Animal Research. (February 2024) 40, no. 1, 10.1186/s42826-024-00194-7. PMC10895735 38409070

[14] Rana S. V. , Pal R. , Vaiphie K. , and Singh K. , Effect of Different Oral Doses of Isoniazid-Rifampicin in Rats, Molecular and Cellular Biochemistry. (September 2006) 289, no. 1, 39–47, 10.1007/s11010-006-9145-3. 16583132

[15] Senior J. R. , Monitoring for Hepatotoxicity: What is the Predictive Value of Liver “Function” Tests?, Clinical Pharmacology and Therapeutics (St. Louis). (March 2009) 85, no. 3, 331–334, 10.1038/clpt.2008.262. 19129750

[16] Vitai M. and Góth L. , Reference Ranges of Normal Blood Catalase Activity and Levels in Familial Hypo Catalasemia in Hungary, Clinica Chimica Acta. (May 1997) 261, no. 1, 35–42, 10.1016/s0009-8981(97)06514-5. 9187503

[17] Pastore A. , Federici G. , Bertini E. , and Piemonte F. , Analysis of Glutathione: Implication in Redox and Detoxification, Clinica Chimica Acta. (July 2003) 333, no. 1, 19–39, 10.1016/s0009-8981(03)00200-6. 12809732

[18] Thalla S. , Ramana V. K. , and Delhiraj N. , Hepatoprotective Effect of Hydroalcoholic Extract of *Ocimum gratissimum* Leaves on rifampicin-isoniazid Induced Rats, Indian Journal of Research in Pharmacy and Biotechnology. (September 2013) 1, no. 5.

[19] Mujahid M. , Siddiqui H. H. , Hussain A. , and Hussain M. S. , Hepatoprotective Effects of *Adenanthera pavonina* (Linn.) Against Anti-Tubercular Drugs-Induced Hepatotoxicity in Rats, Pharmacognosy Journal. (November 2013) 5, no. 6, 286–290, 10.1016/j.phcgj.2013.08.003.

[20] Sagar R. , Kumar P. , Ahmad S. , Kumar H. , and Mandal S. , Hepatoprotective Activity of *Allium cepa* Extract in Antitubercular Drugs Induced Hepatotoxicity in Experimental Animals, European journal of pharmaceutical and Medical Research JPMR. (2022) 9, no. 2, 311–327.

[21] Ilyas N. , Sadiq M. , and Jehangir A. , Hepatoprotective Effect of Garlic *(Allium sativum)* and Milk Thistle (Silymarin) in Isoniazid Induced Hepatotoxicity in Rats, Biomedica. (July 2011) 27, no. 2, 166–170.

[22] Jibril M. M. , Oluchi J. O. , Kabara H. T. , Imam A. A. , Muhammad Y. Y. , and Abdullahi N. , Effect of Homogenates of Avocado Pear *(Persea americana)* Seeds and Fluted Pumpkin *(Telfairia occidentalis)* Leaves Coadministered With Anti-Tuberculosis Drugs on Liver Enzymes of Albino Rats, Bayero Journal of Pure and Applied Sciences. (2015) 8, no. 2, 187–191, 10.4314/bajopas.v8i2.30.

[23] Althaiban M. A. , Evaluation of Hepatoprotective Activity of Neem Extract in Rifampin Induced Acute Hepatic Failure in Rats, International Journal of Pharmaceutical Research and Allied Sciences. (2019) 8, no. 3-2019, 29–36.

[24] Evan P. S. , Udhaya L. B. , Sunitha P. S. , and Arumugam G. , Reparation of Isoniazid and Rifampicin Combinatorial Therapy-Induced Hepatotoxic Effects by *Bacopa monnieri* , Pharmacology. (June 2016) 98, no. 1-2, 29–34. 10.1159/000444856 27007136

[25] Kamil N. and Imran-ul-Haque H. S. , Hepatoprotective Effect of *Calotropis procera* in Isoniazid and Rifampicin Induced Hepatotoxicity, Pharmacognosy Journal. (September 2014) 6, no. 5, 9–14, 10.5530/pj.2014.5.3.

[26] Tamayo A. B. , Abanes M. A. , Biscocho C. J. et al., Hepatoprotective Activity of Methanolic Extract of *Capsicum frutescens* (Silinglabuyo) Against Isoniazid and Rifampicin-Induced Hepatotoxicity in Sprague-Dawley Rats, The Steth. (2014) 8.

[27] Pandit A. , Sachdeva T. , and Bafna P. , Ameliorative Effect of Leaves of *Carica papaya* in Ethanol and Antitubercular Drug Induced Hepatotoxicity, British Journal of Pharmaceutical Research. (October 2013) 3, no. 4, 648–1.

[28] Jaydeokar A. V. , Bandawane D. D. , Bibave K. H. , and Patil T. V. , Hepatoprotective Potential of *Cassia auriculata* Roots on Ethanol and Antitubercular Drug-Induced Hepatotoxicity in Experimental Models, Pharmaceutical Biology. (March 2014) 52, no. 3, 344–355, 10.3109/13880209.2013.837075. 24472085

[29] Ghosh K. , Indra N. , and Jagadeesan G. , The Ameliorating Effect of *Centella asiatica* Ethanolic Extract on Albino Rats Treated with Isoniazid, Journal of Basic and Clinical Physiology and Pharmacology. (January 2017) 28, no. 1, 67–77, 10.1515/jbcpp-2016-0059. 27701138

[30] Sangameswaran Balakrishnan S. B. , Khurana B. S. , Arpita Singh A. S. , Ilango Kaliappan I. K. , and Dubey G. P. , Hepatoprotective Effect of Hydroalcoholic Extract of Cissampelos pareira Against Rifampicin and Isoniazid Induced Hepatotoxicity.

[31] Swamy A. V. , Kulkarni R. V. , Koti B. C. , Gadad P. C. , Thippeswamy A. H. , and Gore A. , Hepatoprotective Effect of *Cissus quadrangularis* Stem Extract Against Rifampicin-Induced Hepatotoxicity in Rats, Indian Journal of Pharmaceutical Sciences. (March 2012) 74, no. 2, 10.4103/0250-474x.103859. PMC3546340 23326004

[32] Pérez-González M. Z. , Macías-Rubalcava M. L. , Hernández-Ortega S. , Siordia-Reyes A. G. , and Jiménez-Arellanes M. A. , Additional Compounds and the Therapeutic Potential of *Cnidoscolus Chayamansa (*McVaugh) Against Hepatotoxicity Induced by Antitubercular Drugs, Biomedicine and Pharmacotherapy. (September 2019) 117, 10.1016/j.biopha.2019.109140. 31387195

[33] Kiyimba K. , Ayikobua E. T. , Mwandah D. C. , and Obakiro S. B. , Assessing the Protective Effect of *Crassocephalum vitellinum* Against Rifampicin-Induced Hepatotoxicity in Wistar Rats, African Health Sciences. (April 2022) 22, no. 1, 352–360. 10.4314/ahs.v22i1.43 PMC9382464 36032444

[34] Dubiwak A. D. , Damtew T. W. , Senbetu M. W. et al., Hepatoprotective Effect of Corm of *Ensete ventricosum (Welw.)* Cheesman Extract Against Isoniazid and Rifampicin Induced Hepatotoxicity in Swiss Albino Mice, Journal of Toxicology. (2021) 2021, no. 1, 4760455–4760458, 10.1155/2021/4760455. PMC8378944 34422040

[35] Gond N. Y. and Khadabadi S. S. , Hepatoprotective Activity of *Ficus carica* Leaf Extract on Rifampicin-Induced Hepatic Damage in Rats, Indian Journal of Pharmaceutical Sciences. (May 2008) 70, no. 3, 10.4103/0250-474x.43003. PMC2792510 20046747

[36] Eram S. , Mujahid M. , Bagga P. , Ahsan F. , and Rahman M. A. , Hepatoprotective Evaluation of Galanga *(Alpinia officinarum)* Rhizome Extract Against Antitubercular Drugs Induced Hepatotoxicity in Rats, Journal of Herbs Spices and Medicinal Plants. (April 2020) 26, no. 2, 113–125, 10.1080/10496475.2019.1679692.

[37] Nasiru A. , Hafsat I. G. , Mohammad M. M. , and Sabo A. A. , Hepatoprotective Effect of Garlic Homogenate Co-Administered With Anti-Tuberculosis Drugs in Rat Liver Enzymes, International Journal of Bioscience, Biochemistry and Bioinformatics. (September 2012) 2, no. 5, 354–357, 10.7763/ijbbb.2012.v2.131.

[38] Lina S. M. , Ashab I. , Bhuiya M. S. , and Shahriar M. , Hepatoprotective Activity of *Hedyotis corymbosa* (Linn*.*) Lam. Extract Against Anti-Tubercular Drug Induced Hepatic Damage in Sprague-Dawley Rats, Pharmaceutical Journal. (August 2018) 21, no. 2, 131–138, 10.3329/bpj.v21i2.37924.

[39] Suresh K. and Singh R. K. , Anti-Hepatotoxic and Antioxidant Influence of *Ipomoea carnea* Against Anti-Tubercular Drugs Induced Acute Hepatopathy in Experimental Rodents, Journal of Coastal Life Medicine. (2013) 1, 293–299.

[40] Reddy G. J. , Reddy V. P. , Sreepavani M. , Rajaram C. , Kumar S. N. , and Kanhere R. S. , Evaluation of Hepatoprotective Potential of Ethanolic Extract of *Ixora pavetta* Against Isoniazid and Rifampicin Induced Hepatotoxicity in Rats, Drug Invention Today. (September 2013) 5, no. 3, 201–206, 10.1016/j.dit.2013.06.007.

[41] Dhamal N. , Patel M. , and Pawar S. , Evaluation of *Jasminum grandiflorum* for Hepatoprotective Activity in Isoniazid Induced Liver Damage, International Journal of Pharmaceutical Sciences and Research. (August 2012) 3, no. 8.

[42] Darvin S. S. , Esakkimuthu S. , Toppo E. et al., Hepatoprotective Effect of *Lawsone* on Rifampicin-Isoniazid Induced Hepatotoxicity in in Vitro and in Vivo Models, Environmental Toxicology and Pharmacology. (July 2018) 61, 87–94, 10.1016/j.etap.2018.05.006. 29859372

[43] Shabbir M. , Afsar T. , Razak S. , Almajwal A. , and Khan M. R. , Phytochemical Analysis and Evaluation of Hepatoprotective Effect of *Maytenus royleanus* Leaves Extract Against Anti-Tuberculosis Drug Induced Liver Injury in Mice, Lipids in Health and Disease. (December 2020) 19, 1–5, 10.1186/s12944-020-01231-9. PMC7077109 32178678

[44] Hafeez A. , Waheed A. , and Yousaf N. , Role of *Nigella sativa* Seeds on Pyrazinamide Induced Hepatotoxicity, Journal of Islamic International Medical College (JIIMC). (March 2019) 14, no. 1, 18–22.

[45] Chaudhary S. , Gupta R. K. , Kumar A. , and Tarazi H. , Hepatoprotective and Antioxidant Potential of *Nyctanthe sarbor-tristis L.* Leaves Against Antitubercular Drugs Induced Hepatotoxicity, Journal of Pharmacy and Pharmacognosy Research. (2018) 6, no. 3, 205–215, 10.56499/jppres17.349_6.3.205.

[46] Radhika J. , Jothi G. , Nivethetha M. , Vijayaroslin L. , and Yuvarani S. , Hepatoprotective Efficacy of Ocimum canum sims, On Isoniazid Induced Hepatotoxicity.

[47] Gugsa E. , Molla T. S. , Bekele T. , and Dejenie T. A. , Hepatoprotective Effect of Hydromethanol Extract of *Otostegia integrifolia benth* Leaves in Isoniazid and Rifampicin Induced Swiss Albino Mice, Metabolism Open. (December 2023) 20, 10.1016/j.metop.2023.100255. PMC10728564 38115863

[48] Ghaia R. , Nagarajanb K. , Groverb P. et al., Bioassay Guided Hepatoprotective Activity of *Polygonatum cirrhifolium* Against Isoniazid and Rifampicin Induced Hepatotoxicity in Rats, Indian Drugs. (July 2023) 60, no. 07, 07–87, 10.53879/id.60.07.13557.

[49] Fayed A. H. , Salama A. A. , Ismaiel I. E. , Attia T. A. , and Hassan A. , Protective Effects of *Solanum nigrum* Methanolic Extract Against Isoniazid/Rifampicin Induced Hepatotoxicity in Rats, Journal of Applied Veterinary Sciences. (October 2019) 4, no. 2, 52–61, 10.21608/javs.2019.63436.

[50] Amir M. , Khan M. A. , Ahmad S. et al., Ameliorating Effects of *Tamarindus indica* Fruit Extract on Anti-Tubercular Drugs Induced Liver Toxicity in Rats, Natural Product Research. (March 2016) 30, no. 6, 715–719, 10.1080/14786419.2015.1039001. 25978515

[51] Nwidu L. L. and Oboma Y. I. , *Telfairia occidentalis* (*Cucurbitaceae*) Pulp Extract Mitigates Rifampicin-Isoniazid-Induced Hepatotoxicity in an in Vivo Rat Model of Oxidative Stress, Journal of Integrative Medicine. (January 2019) 17, no. 1, 46–56, 10.1016/j.joim.2018.11.008. 30555014

[52] Hussain T. , Subaiea G. M. , and Firdous H. , Hepatoprotective Evaluation of *Trapa natans* Against Drug-Induced Hepatotoxicity of Antitubercular Agents in Rats, Pharmacognosy Magazine. (April 2018) 14, no. 54, 10.4103/pm.pm_237_17. PMC5909312 29720828

[53] Tilaye G. A. , Zerihun M. F. , Chuffa K. A. , Arayaselassie M. , and Seifu D. , *Vernonia Amygdalina del* (Bitter Leaf) Extract Ameliorates Isoniazid (INH) Induced Liver Injury in Swiss Albino Mice, BioRxiv. (November 2018) 28.

[54] Tandon V. R. , Khajuria V. , Kapoor B. , Kour D. , and Gupta S. , Hepatoprotective Activity of *Vitex negundo* Leaf Extract Against Anti-Tubercular Drugs Induced Hepatotoxicity, Fitoterapia. (December 2008) 79, no. 7-8, 533–538, 10.1016/j.fitote.2008.05.005. 18621114

[55] Rao C. V. , Rawat A. K. , Singh A. P. , Singh A. , and Verma N. , Hepatoprotective Potential of Ethanolic Extract of *Ziziphus oenoplia* (L.) Mill Roots Against Antitubercular Drugs Induced Hepatotoxicity in Experimental Models, Asian Pacific Journal of Tropical Medicine. (April 2012) 5, no. 4, 283–288, 10.1016/s1995-7645(12)60040-6. 22449519

[56] Himaja N. , Comparative Study of Hepatoprotective Activity of *Acanthospermum hispidum* Plant Extract and Herbal Niosomal Suspension Against Anti-Tubercular Drug Induced Hepatotoxicity in Rats, Asian Journal of Pharmaceutical and Clinical Research. (2015) 8, no. 5, 256–259.

[57] Ishtiaq S. , Afridi M. S. , and Masood N. , Amelioration of Isoniazid and rifampicin-induced Liver Toxicity by *Amaranthus graecizans* Subsp. Silvestris in Rat, Bangladesh Journal of Pharmacology. (September 2017) 12, no. 3, 354–358.

[58] Palanisamy N. and Manian S. , Protective Effects of *Asparagus racemosus* on Oxidative Damage in Isoniazid-Induced Hepatotoxic Rats: an in Vivo Study, Toxicology and Industrial Health. (April 2012) 28, no. 3, 238–244, 10.1177/0748233711410911. 21724661

[59] Lina S. M. , Ashab I. , Ishtiaq Ahmed M. , and Shahriar M. , Hepatoprotective Activity of *Asteracantha Longifolia* (Nees.) Extract Against Anti-Tuberculosis Drugs Induced Hepatic Damage in Sprague-Dawley Rats, Pharmacologyonline. (December 2012) 3, 13–19.

[60] Kosasih E. , Chiuman L. , Lister I. N. , and Fachrial E. , Hepatoprotective Effect of *Citrus Sinensis* Peel Extract Against Isoniazid and Rifampicin-Induced Liver Injury in Wistar Rats, Majalah Obat Tradisional. (December 2019) 24, no. 3, 197–203, 10.22146/mot.45762.

[61] Wali A. F. , Pillai J. R. , Al D. Y. et al., *Crocus Sativus L.* Extract Containing Polyphenols Modulates Oxidative Stress and Inflammatory Response Against Anti-Tuberculosis Drugs-Induced Liver Injury, Plants. (January 2020) 9, no. 2, 10.3390/plants9020167. PMC7076685 32019201

[62] Gopalakrishnan S. B. and Kalaiarasi T. , Hepatoprotective Activity Studies of *Cucumis trigonus ROXB* Against Rifampicin-Isoniazid-Induced Toxicity in Rats, European Journal of Pharmaceutical and Medical Research. (2015) 2, no. 6, 141–146.

[63] Sambrekar S. N. , Patil P. A. , and Kangralkar V. A. , Protective Effect of *Embelia tsjeriam–Cottam* Fruit Extracts on Isoniazid Induced Hepatotoxicity in Wistar Rats, International Journal of Pharma Sciences and Research. (2010) 4, no. 1, 136–139.

[64] Anusuya N. , Raju K. , and Manian S. , Hepatoprotective and Toxicological Assessment of an Ethnomedicinal Plant *Euphorbia fusiformis Buch-Ham*. Ex D. Don, Journal of Ethnopharmacology. (February 2010) 127, no. 2, 463–467, 10.1016/j.jep.2009.10.012. 19837150

[65] Parameswari S. A. , Saleem T. S. , Chandrasekar K. B. , and Chetty C. M. , Protective Role of *Ficus benghalensis* Against Isoniazid-Rifampicin Induced Oxidative Liver Injury in Rat, Revista Brasileira de Farmacognosia. (2012) 22, no. 3, 604–610, 10.1590/s0102-695x2012005000009.

[66] Parameswari S. A. , Chetty C. M. , and Chandrasekhar K. B. , Hepatoprotective Activity of *Ficus religiosa* Leaves Against Isoniazid + Rifampicin and Paracetamol Induced Hepatotoxicity, Pharmacognosy Research. (October 2013) 5, no. 4, 10.4103/0974-8490.118828. PMC3807992 24174821

[67] Rameshwari K. S. and Arockia selvi P. , Anti-Hepatotoxic Effect of *Glinus lotoides* Linn. on Rifampicin Induced Liver Impairment in Albino Rats, Journal of Natural Remedies. (June 2023) 13, 423–427, 10.18311/jnr/2023/29029.

[68] Vyavahare T. , Narwekar M. , Nandedkar S. , and Patil S. , Effect of *Haridra ghrit* in Hepatotoxicity Induced by Antituberculosis Drug–An Animal Study, World Journal of Pharmaceutical Research. (2024) 13, no. Issue 6, 1038–1051.

[69] Bais B. and Saiju P. , Ameliorative Effect of *Leucas cephalotes* Extract on Isoniazid and Rifampicin Induced Hepatotoxicity, Asian Pacific Journal of Tropical Biomedicine. (July 2014) 4, S633–S638, 10.12980/apjtb.4.2014apjtb-2014-0236.

[70] Jadhav V. B. , Thakare V. N. , Suralkar A. A. , Deshpande A. D. , and Naik S. R. , Hepatoprotective Activity of Luffa acutangula Against Ccl 4 and Rifampicin Induced Liver Toxicity in Rats: A Biochemical and Histopathological Evaluation. 21341541

[71] Jyothi B. A. , Mohanalakshmi S. , and Anitha K. , Protective Effect of *Mirabilis jalapa* Leaves on Anti-Tubercular Drugs Induced Hepatotoxicity, Asian Journal of Pharmaceutical and Clinical Research. (2013) 6, no. 3, 221–224.

[72] Ullah I. , Khan J. A. , Adhikari A. , and Shahid M. , Hepatoprotective Effect of *Monotheca buxifolia* Fruit Against Antitubercular Drugs-Induced Hepatotoxicity in Rats, Bangladesh Journal of Pharmacology. (January 2016) 11, no. 1, 248–256, 10.3329/bjp.v11i1.25289.

[73] Pari L. and Kumar N. A. , Hepatoprotective Activity of *Moringa oleifera* on Antitubercular Drug-Induced Liver Damage in Rats, Journal of Medicinal Food. (September 2002) 5, no. 3, 171–177, 10.1089/10966200260398206. 12495589

[74] Ocimum P. L. , Effect of *Ocimum sanctum* (OS) Leaf Extract on Hepatotoxicity Induced by Antitubercular Drugs in Rats, Indian Journal of Physiology and Pharmacology. (2003) 47, no. 4. 15266961

[75] Koppal A. , Praveen S. E. , Shanbhag T. et al., Evaluation of Effect of Ethanolic Extract of *Phyllanthus debilis* on Antitubercular Drugs Induced Hepatotoxicity in Wistar Rats, World Journal of Pharmaceutical Research. (April 2014) 3, no. 4, 1997–2003.

[76] Derangula S. S. , Muthiah N. S. , Babu B. J. , Somashekar H. S. , Sukumar E. , and Prabhu K. , Antioxidant and Hepatoprotective Activities of *Pongamia pinnata* (PP) Linn, Leaves on Anti-Tubercular Medicines (Isoniazid and Rifampin) Induced Hepatotoxicity in Wistar Rats.

[77] Yogeeta S. , Ragavender H. R. , and Devaki T. , Antihepatotoxic Effect of *Punica granatum.* Acetone Extract Against Isoniazid-and Rifampicin-Induced Hepatotoxicity, Pharmaceutical Biology. (January 2007) 45, no. 8, 631–637, 10.1080/13880200701538963.

[78] Khan S. W. , Tahir M. , Lone K. P. , Munir B. , and Latif W. , Protective Effect of *Saccharum officinarum* L.(Sugar Cane) Juice on Isoniazid Induced Hepatotoxicity in Male Albino Mice, Journal of Ayub Medical College, Abbottabad. (June 2015) 27, no. 2, 346–350. 26411113

[79] Kuttiappan A. , Chenchula S. , Vanangamudi M. et al., Hepatoprotective Effect of Flavonoid Rich Fraction of *Sesbania grandiflora*: Results of in Vivo, in Vitro, and Molecular Docking Studies, Journal of Ayurveda and Integrative Medicine. (September 2024) 15, no. 5, 10.1016/j.jaim.2024.101036. PMC11408849 39243548

[80] Ogono T. , Taiwo O. E. , Usman K. et al., An Investigation of the Hepatoprotective Potential of *Garcinia kola* Seed Extract in an Anti-Tubercular Treatment Model, Journal of Medicinal Plants Research. (November 2014) 8, 1156–1163.

[81] Ekor M. , The Growing Use of Herbal Medicines: Issues Relating to Adverse Reactions and Challenges in Monitoring Safety, Frontiers in Pharmacology. (2014) 4, 10.3389/fphar.2013.00177. PMC3887317 24454289

[82] Pan Y. , Tang P. , Cao J. et al., Lipid Peroxidation Aggravates Anti-Tuberculosis Drug-Induced Liver Injury: Evidence of Ferroptosis Induction, Biochemical and Biophysical Research Communications. (December 2020) 533, no. 4, 1512–1518, 10.1016/j.bbrc.2020.09.140. 33121683

[83] Tostmann A. , Boeree M. J. , Aarnoutse R. E. , De Lange W. C. , Van Der Ven A. J. , and Dekhuijzen R. , Antituberculosis Drug‐Induced Hepatotoxicity: Concise Up‐To‐Date Review, Journal of Gastroenterology and Hepatology. (February 2008) 23, no. 2, 192–202, 10.1111/j.1440-1746.2007.05207.x. 17995946

[84] Ramappa V. and Aithal G. P. , Hepatotoxicity Related to Anti-Tuberculosis Drugs: Mechanisms and Management, Journal of Clinical and Experimental Hepatology. (March 2013) 3, no. 1, 37–49, 10.1016/j.jceh.2012.12.001. PMC3940184 25755470

[85] Akkahadsee P. , Sawangjit R. , Phumart P. , Chaiyakunapruk N. , and Sakloetsakun D. , Systematic Review and Network Meta-Analysis of Efficacy and Safety of Interventions for Preventing Anti-Tuberculosis Drug Induced Liver Injury, Scientific Reports. (November 2023) 13, no. 1, 10.1038/s41598-023-46565-3. PMC10645982 37963954

[86] Vargas-Mendoza N. , Madrigal-Santillán E. , Morales-González Á. et al., Hepatoprotective Effect of Silymarin, World Journal of Hepatology. (March 2014) 6, no. 3. 10.4254/wjh.v6.i3.144 PMC3959115 24672644

[87] Kantharia C. , Kumar M. , Jain M. K. , Sharma L. , Jain L. , and Desai A. , Hepatoprotective Effects of Liv. 52 in Chronic Liver Disease Preclinical, Clinical, and Safety Evidence: A Review, Gastroenterology Insights. (July 2023) 14, no. 3, 293–308, 10.3390/gastroent14030021.

[88] Vijaya Padma V. , Suja V. , and Shyamala Devi C. S. , Hepatoprotective Effect of Liv. 52 on Antitubercular Drug Induced Hepatotoxicity in Rats, FITOTERAPIA-MILANO-.(1998) 69, 520–522.

[89] Pe S. K. , Mishra A. , Mandal S. , Chawla S. , and Kalra B. S. , Hepatoprotective Potential of Phyllanthus Niruri and Andrographis Paniculata in Isoniazid-Rifampicin Induced Hepatotoxicity in Rats, Indian Journal of Tuberculosis. (April 2025) 72, no. 2, 189–193, 10.1016/j.ijtb.2023.12.009. 40518209

