# Supplementary material for: Effect of Herbal Extracts on Antituberculosis Drug‐Induced Hepatotoxicity: A Systematic Review
Source: J Toxicol. 2026 Sep 29;2026:6698835. doi: 10.1155/jt/6698835 (PMC13621401; doi:10.1155/jt/6698835)
Supplement: Supplementary file 1 [file JT-2026-6698835-s001.zip › S7.docx]

| **Plant Scientific Name** | **Authors** | **Anti-TB drugs** | **Toxic group (anti-TB drugs) mg/kg** | | **Standard group (Silymarin) mg/kg** | | **Treatment group (PE)**  **mg/kg** | | **% protection by Standard (Silymarin) compared to the Toxic group** | **% protection by Treatment group (PE) compared to the Toxic group**  **(* Mean values are for the highest conc. of PE)** | **% protection by Treatment group (PE) compared to the Standard (Silymarin)** |
| --- | --- | --- | --- | --- | --- | --- | --- | --- | --- | --- | --- |
|  |  |  | **Conc. of drugs mg/kg** | **Mean mg/kg b. w** | **Conc. of Standard mg/kg** | **Mean mg/kg b. w** | **Conc. of PE mg/kg** | **Mean mg/kg b. w** |  |  |  |
| *Abelmoschus ficulneus L.* | Swetha *et al*.,2021 **^(10)^** | INH and RIF | 50 | 5.24 | 100 | 11.63 | 100, 200, 400 | 7.56 | -44.27 | 35.00 | -44.27 |
| *Adenanthera pavonina L.* | Mujahid *et al*.,2013 **^(12)^** | INH and RIF | 50 | 1.48 | 100 | 4.24 | 100 and 200 | 2.33 | -57.43 | 45.05 | -57.43 |
| *Allium cepa L* | Sagar *et al*.,2022 **^(13)^** | INH and RIF | 50 | 427.65 | 100 | 506.46 | 100 | 496.07 | -16.00 | 2.05 | -16.00 |
| *Cassia auriculata L.* | Ashish *et al*.,2014 (**^21)^** | INH, RIF and PZA | 54 | 1.45 | 100 | 0.75 | 300 and 600 | 0.44 | -173.79 | NA | -173.79 |
| *Centella asiatica L.* | Kavisa *et al*.,2016 **^(22)^** | INH | 50 | 3.27 | 50 | 8.41 | 20, 40, 60, and 100 | 8.43 | -176.94 | -28.38 | -176.94 |
| *Cissus quadrangularis L.* | SWAMY *et al*.,2012 **^(24)^** | RIF | 54 | 6.01 | 50 | 11 | 500 | 10.13 | -157.80 | -0.24 | -157.80 |
| *Cnidoscolus chayamansa (McVaugh)* | Mariana *et al.*,2019 **^(25)^** | INH, RIF and PZA | 50 and 100 | 465.67 | 2.5 | 334.33 | 50, 50 and 100 | 213.93 | -68.55 | 7.91 | -68.55 |
| *Alpinia officinarum H.* | Shimaila *et al*.,2019 **^(29)^** | INH and RIF | 50 | 1.24 | 100 | 4.89 | 200 and 400 | 3.63 | 54.06 | 36.01 | 54.06 |
| *Hibiscus vitifolius L.* | Anbu Jeba *et al*.,2012 **^(32)^** | INH, RIF and PZA | 7.5, 10 and 35 | 0.893 | 100 | 3.49 | 400 | 0.98 | -192.74 | 25.77 | -192.74 |
| *Ipomoea carnea J.* | Ramesh Kumar *et al*.,2013 **^(33)^** | INH, RIF and PZA | 7.5, 10 and 35 | 12.73 | 100 | 21.32 | 125,250 and 500 | 13.21 | -157.04 | NA | -157.04 |
| *Nyctanthes arbor-tristis L.* | Chaudhary *et al*.,2018 **^(39)^** | INH, RIF and PZA | 7.5, 10 and 35 | 8.9 | 100 | 21.9 | 250 and 500 | 13.1 | -9.74 | 71.92 | -9.74 |
| *Ocimum canum Sims.* | Radhika J *et al*.,2018 **^(40)^** | INH | 54 | 0.87 | 50 | 1.23 | 200 and 300 | 1.36 | -3.77 | 38.04 | -3.77 |
| *Trapa natans L.* | Hussain *et al*.,2018 **^(46)^** | INH and RIF | 50 | 9.52 | 100 | 4.18 | 200 and 400 | 1.84 | -57.59 | NA | -57.59 |
| *Ziziphus oenoplia L.* | Ch V Rao *et al.*,2012 **^(49)^** | INH and RIF | 50 | 16.04 | 100 | 18.87 | 150 and 300 | 13.83 | -47.19 | 40.18 | -47.19 |
| *Cucumis trigonus Roxb.* | Gopalakrishnan SB *et al.,*2015 (**^56)^** | INH and RIF | 50 | 0.073 | 2.5 | 21.78 | 100, 250 and 500 | 19.84 | -56.32 | -10.57 | -56.32 |
| *Embelia tsjeriam-cottam A.DC.* | Sambrekar SN *et al.,*2010 **^(57)^** | INH | 50 | 19.16 | 5 ml | 0.203 | 200 | 0.129 | -4.55 | 55.98 | -4.55 |
| *Leucas cephalotes R.* | Bais B *et al.,* 2014 **^(63)^** | INH and RIF | 100 | 19.21 | 200 | 32.92 | 200 and 400 | 36.3 | -45.27 | 26.71 | -45.27 |
| *Mirabilis jalapa L.* | Jyothi BA *et al.*,2013 **^(65)^** | INH, RIF, PZA and EMB | 27, 40, 66 and 53 | 1.62 | 100 | 35.06 | 250 and 500 | 31.24 | -23.69 | 8.91 | -23.69 |
| *Moringa oleifera L.* | Pari L *et al.,*2002 **^(67)^** | INH, RIF and PZA | 7.5, 10 and 35 |  | 200 | 3.71 | 150, 200 and 250 | 2.08 | -13.77 | NA | -13.77 |
| *Mucuna pruriens L.* | Obogwu MB *et al*.,2014 **^(68)^** | INH and RIF | 100 | 1.072 | 50 | 1.945 | 100,200 and 400 | 2.203 | -76.71 | 36.45 | -76.71 |
| *Sesbania grandiflora L.* | Kuttiappan A *et al.,*2024 **^(74)^** | INH, RIF and PZA | 0.5 | 4.17 | 100 | 4.12 | 250 and 500 | 3.12 | -89.46 | -10.27 | -89.46 |

***Supplementary table no 7: Effect of plant extracts on the SOD levels compared with toxic group (anti-TB drugs) and Silymarin (standard)***
