# Supplementary material for: Effect of Herbal Extracts on Antituberculosis Drug‐Induced Hepatotoxicity: A Systematic Review
Source: J Toxicol. 2026 Sep 29;2026:6698835. doi: 10.1155/jt/6698835 (PMC13621401; doi:10.1155/jt/6698835)
Supplement: Supplementary file 1 [file JT-2026-6698835-s001.zip › Supplementary files 20260527 SR.docx]

**Supplementary files**

**Search words for Pubmed:**

TB OR Consumption OR Phthisis OR “White Plague” OR “Koch's Disease” OR “Pulmonary tuberculosis” OR “Lung TB” OR “TB infection” OR “Tubercular disease” OR “Tubercle bacillus infection” OR Tuberculosis OR Tuberculoses OR “Mycobacterium tuberculosis Infection” OR “Pulmonary phthisis” OR “White plague” OR Scrofula OR “Kings Touch” AND “Herbal remedies” OR “Medicinal herbs” OR “Traditional herbs” OR “Ethnobotanical plants” OR “Medicinal botanicals” OR “Indigenous remedies” OR “Herbal medicines” OR “Folk remedies” OR “Plant-based therapies” OR “Natural remedies” OR “Traditional Medicinal Plants” OR “Medicinal herbs” OR “Drug yielding plant” OR “Healing plant” OR “Herbal medicinal products” OR “Herbal medicine” OR “Medicinal grasses” OR “Traditional herbs” OR “Traditional herbal medicine” AND “Liver toxicity” OR “Hepatic toxicity” OR “Liver damage” OR “Hepatic injury” OR “Liver harm” OR “Hepatocellular injury” OR “Hepatocellular damage” OR “Hepatic adverse effects” OR “Liver impairment” OR “Liver dysfunction” OR “Drug induced hepatotoxicity” OR Hepatotoxicity OR “Liver damage” OR “Liver injury” OR “Liver failure” OR “Liver disease” OR “Liver lesions” AND “Tuberculosis medications” OR “TB antibiotics” OR “TB drugs” OR “Anti-tubercular agents” OR “Anti-TB medications” OR “Tuberculosis therapy” OR “TB treatment drugs” OR “Anti-Mycobacterial drugs” OR “Anti-tuberculosis medicines” OR “TB chemotherapeutic agents” OR “Anti-tubercular medications” OR “Anti- Tubercular drugs” OR “Anti-Tb drugs” OR “Anti-mycobacterial drugs” OR “Anti-tubercular drugs” OR “anti-TB agent” OR “Anti-Tb regimen” OR “First line anti-TB drugs OR antimycobacterials.

***Supplementary table no.1: Showing Summary of Study Parameters and Experimental Details***

| **Scientific Name** | **PP** | **Country** | **Animals** | **Drug** | **Drug conc** | **No. of animals** | **Conc mg/kg** | **No of days** |  |  | **Biochemical parameters** | | |  |  |  |  | **Oxidative parameter** |  |  |  |  | **References** |
| --- | --- | --- | --- | --- | --- | --- | --- | --- | --- | --- | --- | --- | --- | --- | --- | --- | --- | --- | --- | --- | --- | --- | --- |
|  |  |  |  |  |  |  |  |  | **Groups** | **AST (U/L)** | **ALT**  **(U/L)** | **ALP**  **(U/L)** | **TBil mg/dl** | **DB mg/dl** | **TP g/dl** | **ALB g/dl** | **LDH U/L** | **LPO mM/mg** | **Groups** | **GSH mM/mg** | **CAT U/mg** | **SOD U/mg** |  |
| *Abelmoschus*  *Ficulneus L.* | Root | India | Male and Female Wistar rats | Isoniazid and rifampicin | 50 mg/kg, | n=36 | 100, 200 and 400 | 15 days | Normal | 48.58 ±3.51 | 54.17± 2.53 | 150.91± 5.42 | 0.51 ± 0.03 | 0.25± 0.02 | 6.3±1.21 | 3.9 ± 0.54 | 180.17± 4.24 | 2.54±0.03 | Normal | 6.55±1.02 | 15.75±0.12 | 13.54±0.15 |  |
|  |  |  |  |  |  |  |  |  | toxic | 85.64 ± 4.27 | 119.19± 8.55 | 259.13± 9.11 | 2.63 ± 0.51 | 1.89 ± 0.55 | 2.1± 0.32 | 2.2± 0.04 | 495.13± 9.54 | 5.12±0.12 | toxic | 1.63±0.42 | 3.35±0.31 | 5.25±0.21 |  |
|  |  |  |  |  |  |  |  |  | Standard | 54.13±2.78 | 59.13±3.11 | 145.24± 6.52 | 0.55 ± 0.03 | 0.27± 0.02 | 5.8± 1.11 | 3.7±0.25 | 160.21±7.51 | 2.98±0.02 | Standard | 5.95±0.11 | 14.58±0.24 | 11.63±0.08 |  |
|  |  |  |  |  |  |  |  |  | Test 100 | 73.51± 3.55 | 94.15±6.37 | 203.51± 9.25 | 1.13± 0.10 | 0.81±0.08 | 3.7±0.31 | 2.5± 0.24 | 350.31± 9.95 | 4.33±0.14 | Test 100 | 3.24±0.09 | 6.74±0.03 | 7.56±0.51 |  |
|  |  |  |  |  |  |  |  |  | Test 2 | 67.87± 3.45 | 83.58±5.55 | 159.54± 5.69 | 0.82 ± 0.05 | 0.43± 0.08 | 4.6±0.27 | 3.1± 0.33 | 239.43±9.56 | 3.56±0.22 | Test 2 | 4.66±0.03 | 9.54±0.11 | 9.58±0.23 |  |
|  |  |  |  |  |  |  |  |  | Test 3 | 59.35±2.55 | 65.31± 4.58 | 139.42± 4,.54 | 0.63 ± 0.02 | 0.29± 0.04 | 5.4± 0.51 | 3.5±0.27 | 189.55±5.63 | 3.18±0.04 | Test 3 | 5.69±0.21 | 12.56±0.45 | 10.75±0.33 | Urugonda S, Vanapatla SR. HEPATOPROTECTIVE EFFECT OF ABELMOSCHUS FICULNEUS ROOT EXTRACT AGAINST DRUG INDUCED HEPATOTOXICITY CAUSED BY THE COMBINED ACTION OF ISONIAZID AND RIFAMPICIN. Journal of Advanced Scientific Research. 2021 Dec 31;12(04 Suppl 1):163-70. |
| *Adenantherapavonina (L.)* | leaves | India | Male Sprague-Dawley rats | Isoniazid and rifampicin | 50 mg/kg body wt. | n=6 | 100 and 200 | 28 days | Normal Control | 79.172± 1.407 | 51.48± 0.834 | 151.59± 3.389 |  |  | 6.72± 0.072 | 2.94± 0.049 | 340.87± 2.652 | 25.42± 0.915 | Normal Control | 4.14± 0.009 | 16.13± 0.654 | 4.68± 0.274 |  |
|  |  |  |  |  |  |  |  |  | Hepatotoxic (INH + RIF) | 146.41± 1.991 | 162.83± 2.476 | 290.36± 3.190 |  |  | 3.72± 0.170 | 1.420± 0.121 | 577.79± 2.638 | 72.92± 0.713 | Hepatotoxic (INH + RIF) | 1.61 ± 0.007 | 8.66± 0.377 | 1.48± 0.064 |  |
|  |  |  |  |  |  |  |  |  | MEAP 100 mg | 90.131± 2.407 | 93.95± 1.267 | 175.10 ± 2.926 |  |  | 4.77± 0.134 | 2.09± 0.027 | 358.12± 2.718 | 62.47± 1.282 | MEAP 100 mg | 2.00 ± 0.009 | 11.04± 0.395 | 2.33± 0.097 |  |
|  |  |  |  |  |  |  |  |  | MEAP 200 mg | 84.143± 1.991 | 74.07 ± 3.801 | 164.68 ± 1.863 |  |  | 5.50± 0.155 | 2.25± 0.112 | 352.45± 1.44 | 39.57± 0.614 | MEAP 200 mg | 2.07± 0.006 | 12.63± 0.616 | 3.72± 0.105 |  |
|  |  |  |  |  |  |  |  |  | Silymarin 100mg | 81.177± 1.407 | 66.54 ± 3.590 | 156.31± 2.028 |  |  | 5.66± 0.219 | 2.27± 0.054 | 348.12± 2.718 | 31.86± 1.190 | Silymarin 100mg | 3.22± 0.008 | 14.32± 0.544 | 4.24± 0.163 | Mujahid M, Siddiqui HH, Hussain A, Hussain MS. Hepatoprotective effects of Adenanthera pavonina (Linn.) against anti-tubercular drugs-induced hepatotoxicity in rats. Pharmacognosy Journal. 2013 Nov 1;5(6):286-90. |
| *Allium cepa L.* | whole plant | India | Wistar strain rats | Isoniazid and rifampicin | 100 mg/kg | n=6 | 100mg/kg | 28 | G1 (Normal control, Standard  diet, and water) | 129.96 ± 5.11 | 0.32 ± 0.02 |  | 0.11± 0.01 |  |  |  |  | 94.20 ± 3.97 | G1 (Normal control, Standard  diet, and water) | 26.84 ±1.43 |  | 649.41± 52.39 |  |
|  |  |  |  |  |  |  |  |  | G2 (Disease control, Anti-TB Drug+ Standard diet and water) | 396.71 ± 57.06 | 0.21 ± 0.02 |  | 0.15 ± 0.02 |  |  |  |  | 17.63± 1.56 | G2 (Disease control, Anti-TB Drug+ Standard diet and water) | 24.53 ± 1.21 |  | 427.65 ± 47.07 |  |
|  |  |  |  |  |  |  |  |  | G3 (Anti-TB Drug + Silymarin (100mg/kg) oral) | 428.28 ± 70.71 | 0.29 ± 0.06 |  | 0.14 ± 0.03 |  |  |  |  | 14.40 ±1.12 | G3 (Anti-TB Drug + Silymarin (100mg/kg) oral) | 37.90 ± 4.92 |  | 506.46 ± 25.39 |  |
|  |  |  |  |  |  |  |  |  | G4 Anti-TB Drug + Allium-cepa Extract (100mg/kg) | 495.01 ± 45.91 | 0.24 ± 0.02 |  | 0.19 ± 0.04 |  |  |  |  | 16.58 ± 1.26 | G4 Anti-TB Drug + Allium-cepa Extract (100mg/kg) | 27.79 ± 1.04 |  | 496.07 ± 28.23 | Sagar R, Kumar P, Ahmad S, Kumar H, Mandal S. HEPATOPROTECTIVE ACTIVITY OF ALLIUM CEPA EXTRACT IN ANTITUBERCULAR DRUGS INDUCED HEPATOTOXICITY IN EXPERIMENTAL ANIMALS. |
| *Allium sativum L.* | whole part | Islamabad | male rats | Isoniazid (INH) | 50 mg/kg | n=10 | 0.25 g/kg b | 28 days | Group A | 101.4± 1.87 | 30.5± 0.83 | 123.6± 1.31 | 0.477± 0.04 |  |  |  |  |  | Group A |  |  |  |  |
|  |  |  |  |  |  |  |  |  | Group B | 272.7± 1.56 | 130.9±1.96 | 385.6± 2.55 | 2.4673± 0.11 |  |  |  |  |  | Group B |  |  |  |  |
|  |  |  |  |  |  |  |  |  | Group C | 127.4± 1.56 | 36.4± 0.62 | 143.8± 1.18 | 0.629± 0.03 |  |  |  |  |  | Group C |  |  |  |  |
|  |  |  |  |  |  |  |  |  | Group D | 98.5± 1.22 | 28.1± 0.51 | 118.4± 1.22 | 1.328± 0.03 |  |  |  |  |  | Group D |  |  |  | Ilyas N, Sadiq M, Jehangir A. Hepatoprotective effect of garlic (Allium sativum) and milk thistle (silymarin) in isoniazid induced hepatotoxicity in rats. Biomedica. 2011 Jul;27(2):166-70. |
| *Annona*  *squamosa L.* | leaves | India | Male Wistar albino rats, | Isoniazid and rifampicin | 100 mg/kg, Ip) | n=6 | 250 and 500 mg/kg bw, p.o | 21 days | Normal group (G1) | 118.39± 5.99 | 30.99± 0.38 | 117.39± 2.1 | 0.35± 0.01 |  | 6.47 ± 0.01 |  |  |  | Normal group (G1) |  |  |  |  |
|  |  |  |  |  |  |  |  |  | Toxic group (G2) | 368.72 ± 7.25 | 124.91± 2.64 | 243.64± 7.55 | 1.06 ±0.04 |  | 4.35± 0.04 |  |  |  | Toxic group (G2) |  |  |  |  |
|  |  |  |  |  |  |  |  |  | MEAS 250 mg/kg (G3) | 164.84± 1.66 | 67.02± 2.75 | 150.55 ± 11.49 | 0.84 ±0.6 |  | 7.3± 0.11 |  |  |  | MEAS 250 mg/kg (G3) |  |  |  |  |
|  |  |  |  |  |  |  |  |  | MEAS 500 mg/kg (G4) | 1.66.96 ± 4.39 | 79.46± 575 | 151.99 ± 9.39 | 0.62 ± 0.14 |  | 7.8 ± 0.11 |  |  |  | MEAS 500 mg/kg (G4) |  |  |  |  |
|  |  |  |  |  |  |  |  |  | SIL group (G5) | 147.88 ± 3.82 | 49.52± 3.48 | 111.29± 11.23 | 0.42± 0.03 |  | 7.85± 0.01 |  |  |  | SIL group (G5) |  |  |  | Uduman TS, Sundarapandian R, Muthumanikkam A, Kalimuthu G, Parameswari SA, Vasanthi Srinivas TR, Karunakaran G. Protective effect of methanolic extract of Annona squamosa Linn in isoniazid-rifampicin induced hepatotoxicity in rats. Pak. J. Pharm. Sci. 2011 Apr 1;24(2):129-34. |
| *Persea americana Mill. and Telfairia occidentalis Hook.* | leaves and seed | Kano State | albino rats | Rifampicin +Isoniazid + Pyrazinamide +Ethambutol | 51.4mg/kg | n=2 | 250mg/kg | 7 and 14 days | Group 1 (anti-tb drugs) after 7 days | 47.00 ± 0.28 | 54.10 ± 2.90 | 86.90 ± 0.21 |  |  |  |  |  |  | Group 1 (anti-tb drugs) after 7 days |  |  |  |  |
|  |  |  |  |  |  |  |  |  | Group 2 (Avocado seed) | 38.50 ± 3.54 | 40.40 ± 3.68 | 76.40 ± 0.14 |  |  |  |  |  |  | Group 2 (Avocado seed) |  |  |  |  |
|  |  |  |  |  |  |  |  |  | Group 3 (Fluted pumpkin leaves) | 27.20 ± 0.21 | 23.40 ± 2.33 | 57.00 ± 0.71 |  |  |  |  |  |  | Group 3 (Fluted pumpkin leaves) |  |  |  |  |
|  |  |  |  |  |  |  |  |  | Group 4 (APS and FPL) | 17.00± 0.50 | 15.80 ± 0.00 | 38.60 ± 0.57 |  |  |  |  |  |  | Group 4 (APS and FPL) |  |  |  |  |
|  |  |  |  |  |  |  |  |  | Group 5 (Negative control) | 10.20 ± 0.21 | 10.00 ± 0.13 | 33.00 ± 0.07 |  |  |  |  |  |  | Group 5 (Negative control) |  |  |  | Jibril MM, Oluchi JO, Kabara HT, Imam AA, Muhammad YY, Abdullahi N. Effect of homogenates of avocado pear (Persea americana) seeds and fluted pumpkin (Telfairia occidentalis) leaves coadministered with anti-tuberculosis drugs on liver enzymes of albino rats. Bayero Journal of Pure and Applied Sciences. 2015;8(2):187-91. |
|  |  |  |  |  |  |  |  |  |  |  |  |  |  |  |  |  |  |  |  |  |  |  |  |
|  |  |  |  |  |  |  |  |  | Group 1 (anti-tb drugs) after 14 days | 56.30 ± 1.49 | 56.90 ± 0.14 | 96.80 ± 0.00 |  |  |  |  |  |  | Group 1 (anti-tb drugs) after 14 days |  |  |  |  |
|  |  |  |  |  |  |  |  |  | Group 2 (Avocado seed) | 33.30 ± 0.21 | 43.90 ± 0.14 | 70.40 ± 0.64 |  |  |  |  |  |  | Group 2 (Avocado seed) |  |  |  |  |
|  |  |  |  |  |  |  |  |  | Group 3 (Fluted pumpkin leaves) | 19.50 ± 0.71 | 18.10 ± 0.50 | 55.20 ± 0.35 |  |  |  |  |  |  | Group 3 (Fluted pumpkin leaves) |  |  |  |  |
|  |  |  |  |  |  |  |  |  | Group 4 (APS and FPL) | 14.50 ± 2.12 | 13.20 ± 0.21 | 36.40 ± 0.23 |  |  |  |  |  |  | Group 4 (APS and FPL) |  |  |  |  |
|  |  |  |  |  |  |  |  |  | Group 5 (Negative control) | 11.20 ± 0.21 | 09.68 ± 0.11 | 34.40 ± 0.14 |  |  |  |  |  |  | Group 5 (Negative control) |  |  |  |  |
| *Azadirachta indica L.* | leaves | KSA. | Sprague Dawley adult male rats | Rifampin | 54 mg/kg/day | n=10 | 250 mg/kg/day and 500 mg/kg/day | 30 days | Control group | 53.56 ± 1.65 | 48.21 ± 0.53 | 58.92± 2.15 |  |  |  |  |  |  | Control group | 6.27 ± 0.03 |  | 57.34 ± 1.95 |  |
|  |  |  |  |  |  |  |  |  | RIF group | 141.19 ± 2.13 | 130.36 ± 0.26 | 143.22 ± 2.43 |  |  |  |  |  |  | RIF group | 3.87 ± 0.09 |  | 23.46 ± 1.45 |  |
|  |  |  |  |  |  |  |  |  | NMLE (250 mg/kg) + RIF | 67.53 ± 2.67 | 75.37 ± 2.85 | 81.32 ± 1.45 |  |  |  |  |  |  | NMLE (250 mg/kg) + RIF | 5.07 ± 0.05 |  | 64.23 ± 1.74 |  |
|  |  |  |  |  |  |  |  |  | NMLE (500mg/kg) + RIF | 56.20 ± 3.43 | 54.64 ± 0.62 | 62.59± 2.21 |  |  |  |  |  |  | NMLE (500mg/kg) + RIF | 6.14 ± 0.08 |  | 59.03 ± 1.12 | Althaiban MA. Evaluation of Hepatoprotective Activity of Neem Extract in Rifampin Induced Acute Hepatic Failure in Rats. International Journal of Pharmaceutical Research & Allied Sciences. 2019 Jul 1;8(3). |
| *Bacopa monnieri L.* | whole plant | India | Female Wistar albino rats | Isoniazid and rifampicin | 50 mg/kg | n=6 | 500 mg/kg | 28 days | Group I | 96.00 ± 1.47 | 48.00 ± 1.41 | 98 ± 1.41 | 0.60 ± 0.14 | 0.30 ± 0.14 | 7.3 ± 0.14 | 4.16 ± 0.16 |  |  | Group I |  |  |  |  |
|  |  |  |  |  |  |  |  |  | Group II | 185.00 ± 1.41 | 103 ±1.41 | 198 ± 1.41 | 2.30 ± 0.14 | 2.10 ± 0.14 | 4.2 ±0.14 | 1.50 ± 0.14 |  |  | Group II |  |  |  |  |
|  |  |  |  |  |  |  |  |  | Group III | 98.00 ± 1.41 | 45.00 ± 1.41 | 104 ± 1.41 | 0.80 ± 0.14 | 0.80 ± 0.14 | 6.10 ± 0.14 | 3.10 ± 0.1.4 |  |  | Group III |  |  |  |  |
|  |  |  |  |  |  |  |  |  | Group IV | 96.00 ± 1.41 | 47.00 ± 1.41 | 106 ± 1.41 | 0.70 ± 0.14 | 0.90 ± 0.14 | 6.70 ± 0.14 | 2.90 ± 0.14 |  |  | Group IV |  |  |  |  |
|  |  |  |  |  |  |  |  |  | Group V | 94.00 ± 1.41 | 48.00 ± 1.63 | 80 ± 2.00 | 0.40 ± 0.14 | 0.48 ± 0.15 | 6.50 ± 0.14 | 4.10 ± 0.14 |  |  | Group V |  |  |  | Evan Prince S, Udhaya LB, Sunitha PS, Arumugam G. Reparation of isoniazid and rifampicin combinatorial therapy-induced hepatotoxic effects by Bacopa monnieri. Pharmacology. 2016 Jun 6;98(1-2):29-34. |
| *Calotropis procera A.* | flowers | Karachi | Adult Wistar rats | Isoniazid and rifampicin | 100 and 150 | n=6 | 150 mg/kg | 2 weeks | Group A | 133.25±10.2 | 20.16±4.49 | 74.00±7.2 | 0.22±0.05 | 0.06±0.02 |  |  |  |  | Group A |  |  |  |  |
|  |  |  |  |  |  |  |  |  | Group B | 189.92±17.3 | 41.83±7.09 | 154.67±18.5 | 0.61±0.07 | 0.17±0.03 |  |  |  |  | Group B |  |  |  |  |
|  |  |  |  |  |  |  |  |  | Group C | 113.5±8.7 | 13.83±2.72 | 83.33±11.3 | 0.37±0.03 | 0.11±0.02 |  |  |  |  | Group C |  |  |  | Kamil N, Imran-ul-Haque HS. Hepatoprotective Effect of Calotropis procera in Isoniazid and Rifampicin Induced Hepatotoxicity. Pharmacognosy Journal. 2014 Sep 1;6(5). |
| *C. frutescens L.* | leaves | Batangas City | Adult Sprague-Dawley rats both sexes albino mice | Isoniazid and rifampicin | 50 mg/kg | n=5 | 100 and 200 mg/kg | 14 | Group I Control (saline) 5 ml/kg | 123.8±19.85 | 36±5.47 |  |  |  |  |  |  |  | Group I Control (saline) 5 ml/kg |  |  |  |  |
|  |  |  |  |  |  |  |  |  | Group II | 151.6±17.31 | 39.4± 8.57 |  |  |  |  |  |  |  | Group II |  |  |  |  |
|  |  |  |  |  |  |  |  |  | Group III | 0.8±13.27 | 36±6.33 |  |  |  |  |  |  |  | Group III |  |  |  |  |
|  |  |  |  |  |  |  |  |  | Group IV | 100.4±26.46 | 57.2±7.52 |  |  |  |  |  |  |  | Group IV |  |  |  | Tamayo AB, Abanes MA, Biscocho CJ, Comia NJ, Marasigan ND, Sabanal HM, Cabanela RA, Dumaoal OS. Hepatoprotective activity of methanolic extract of Capsicum frutescens (silinglabuyo) against isoniazid and rifampicin-induced hepatotoxicity in Sprague-Dawley rats. |
| *Carica papaya L..* | leaves | India | Albino Wistar rats | Isoniazid and rifampicin | 150 mg/kg | n=6 | 400mg/kg | 10 days | Group 1 | 31.40 ± 0.50 | 37.51 ± 0.55 | 28.82 ± 0.22 | 0.39 ± 0.03 |  | 2.68 ± 0.06 |  |  |  | Group 1 | 1.83 ± 0.05 |  |  |  |
|  |  |  |  |  |  |  |  |  | Group 2 | 308.40 ± 18.16 | 218.80 ± 14.70 | 251.90 ± 10.78 | 1.71 ± 0.05 |  | 1.37 ± 0.04 |  |  |  | Group 2 | 0.54 ± 0.02 |  |  |  |
|  |  |  |  |  |  |  |  |  | Group 3 | 175.3 ± 4.4 | 139 ± 5.53 | 150.5 ± 5.7 | 0.82 ± 0.05 |  | 1.70 ± 0.01 |  |  |  | Group 3 | 0.75 ± 0.02 |  |  |  |
|  |  |  |  |  |  |  |  |  | Group 4 | 138.90 ± 4.61 | 108.90 ± 4.19 | 124.00 ± 4.2 | 0.63 ± 0.03 |  | 2.01 ± 0.05 |  |  |  | Group 4 | 1.48 ± 0.04 |  |  | Aashish Pandit AP, Tarun Sachdeva TS, Pallavi Bafna PB. Ameliorative effect of leaves of Carica papaya in ethanol and antitubercular drug induced hepatotoxicity. |
| *Cassia auriculata L.* | root | India | Wistar albino rats of either sex | Isoniazid, rifampicin and pyrazinamide | 27, 54 and 135 mg/kg | n=6 | 300 and 600 mg/kg | 30 days | Group I NC | 203.73 ± 5.90 | 53.00 ± 5.58 | 176.4 ± 4.00 | 1.05 ± 0.29 | 7.85 ± 1.57 |  | 6.83 ± 0.23 |  |  | Group I NC |  |  | 5.96± 1.07 |  |
|  |  |  |  |  |  |  |  |  | Group II EC | 08.25 ± 5.44 | 135.53 ± 21.35 | 314.16 ± 18.26 | 2.54 ± 0.19 | 5.51± 0.14 |  | 4.82± 0.38 |  |  | Group II EC |  |  | 1.45 ± 0.67 |  |
|  |  |  |  |  |  |  |  |  | Group III EC+ Self-recovery | 252.75 ± 12.18 | 101.87 ± 3.96 | 268.66 ± 19.33 | 2.53 ± 0.27 | 3.64± 0.62 |  | 3.03 ± 0.25 |  |  | Group III EC+ Self-recovery |  |  | 0.44 ± 0.01 |  |
|  |  |  |  |  |  |  |  |  | Group IV EC + MECA 300 mg/kg | 289.26 ± 19.63 | 70.70 ± 4.80 | 253.6 ± 16.16 | 1.87 ± 0.21 | 7.28 ± 0.33 |  | 2.95 ± 0.33 |  |  | Group IV EC + MECA 300 mg/kg |  |  | 0.44± 0.01 |  |
|  |  |  |  |  |  |  |  |  | Group V EC + MECA 600 mg/kg | 242.18 ± 9.49 | 68.50 ± 14.45 | 176.4 ± 12.52 | 1.42 ± 0.30 | 7.81 ± 0.77 |  | 3.87± 0.41 |  |  | Group V EC + MECA 600 mg/kg |  |  | 0.98 ± 0.13 |  |
|  |  |  |  |  |  |  |  |  | Group VI EC + Silymarin 100 mg/kg | 225.90 ±15.35 | 75.6 ± 10.07 | 186.01 ± 21 | 141.0.18 | 7.70 ± 0.25 |  | 4.01 ± 0.35 |  |  | Group VI EC + Silymarin 100 mg/kg |  |  | 0.75 ± 0.01 | Jaydeokar AV, Bandawane DD, Bibave KH, Patil TV. Hepatoprotective potential of Cassia auriculata roots on ethanol and antitubercular drug-induced hepatotoxicity in experimental models. Pharmaceutical biology. 2014 Mar 1;52(3):344-55. |
| *Centella asiatica L.* | leaves | India | Male Wistar albino rats | Isoniazid | 50 mg/kg | n=8 | 20, 40, 60, and 100 mg/kg | 30 days | Group 1 (control) | 16.62 ± 3.70 | 6.24 ± 2.40 | 28.31 ± 4.44 | 0.39 ± 0.03 |  | 6.60 ± 0.12 | 3.80 ± 0.02 |  |  | Group 1 (control) | 34.98 ± 3. 55 | 1. 04 ± 0.10 | 8.45 ± 0.23 |  |
|  |  |  |  |  |  |  |  |  | Group 2 (INH) | 50. 62 ± 6.66 | 26.23 ± 8.76 | 51.43 ± 6.13 | 0.54 ± 0.09 |  | 4.51 ± 0.28 | 2.12 ± 0.06 |  |  | Group 2 (INH) | 21.15 ± 2.49 | 0.32 ± 0.02 | 3.27 ± 0.09 |  |
|  |  |  |  |  |  |  |  |  | Group 3 (INH + 20 mg/kg CA) | 45.02 ± 8.87 | 24.04 ± 5.52 | 50.49 ± 9.32 | 0.50 ± 0.04 |  | 4.68 ± 0.15 | 2.42 ± 0.13 |  |  | Group 3 (INH + 20 mg/kg CA) | 21.12 ± 3.49 | 0.54 ± 0.01 | 3.29 ± 0.13 |  |
|  |  |  |  |  |  |  |  |  | Group 4 (INH +_40 mg/kg CA) | 40. 98 ± 7.98 | 20. 19 ± 7.46 | 45.12 ± 4.56 | 0.46 ± 0.06 |  | 5.03 ± 0.34 | 2.89 ± 0.15 |  |  | Group 4 (INH +_40 mg/kg CA) | 25. 01 ± 3.55 | 0.72 ± 0.03 | 4.79 ± 0.06 |  |
|  |  |  |  |  |  |  |  |  | Group 5 (INH + 60 mg/kg CA) | 31. 26 ± 11.08 | 18.35 ± 5.88 | 34.32 ± 3.79 | 0.42 ± 0.09 |  | 5.37 ± 0.11 | 3.29 ± 0.20 |  |  | Group 5 (INH + 60 mg/kg CA) | 27.44 ± 3.73 | 0.85 ± 0.04 | 6.23 ± 0.11 |  |
|  |  |  |  |  |  |  |  |  | Group 6 (INH + 100 mg/ kg CA) | 16.88 ± 4.58 | 6.72 ± 3.454 | 30.11 ± 4.78 | 0.40 ± 0.06 |  | 5.58 ± 0.34 | 3.62 ± 0.24 |  |  | Group 6 (INH + 100 mg/ kg CA) | 32.72 ± 3.45 | 1.00 ± 0.14 | 8.43 ± 0.03 |  |
|  |  |  |  |  |  |  |  |  | Group 7 (CA 100 mg/kg) | 16.38 ± 6.04 | 6.01 ± 2.69 | 27. 91 ± 4.68 | 0.37 ± 0.02 |  | 6.58 ± 0.24 | 3.75 ± 0.19 |  |  | Group 7 (CA 100 mg/kg) | 37.72 ± 2.35 | 1.07 ± 0.23 | 8.47 ± 0.02 |  |
|  |  |  |  |  |  |  |  |  | Group 8 (INH /SIL 50 mg/kg) | 18.32 ± 5.52 | 6.97 ± 2.53 | 35.79 ± 4.34 | 0.41 ± 0.09 |  | 5.55 ± 0.46 | 3.65 ± 0.20 |  |  | Group 8 (INH /SIL 50 mg/kg) | 31. 81 ± 3.51 | 0.98 ± 0.10 | 8.41 ± 0.072 | Ghosh K, Indra N, Jagadeesan G. The ameliorating effect of Centella asiatica ethanolic extract on albino rats treated with isoniazid. Journal of Basic and Clinical Physiology and Pharmacology. 2017 Jan 1;28(1):67-77. |
| *Cissampelos pareira L.* | roots | India | Sprague-Dawley rats and Swiss albino mice | Isoniazid and rifampicin | 50 mg/kg | n= 6 | 100, 200 and 400 mg/kg | 28 days | Group 1 (control) | 171.53 ± 6.50 | 25.55 ± 3.04 | 229.50 ± 10.43 | 0.54± 0.03 |  | 6.87 ± 0.43 | 1.32 ± 0.03 |  |  | Group 1 (control) |  |  |  |  |
|  |  |  |  |  |  |  |  |  | Group 2 (RIF +INH) | 376.40 ± 8.55 | 82.33 ± 5.61 | 389.78 ± 8.24 | 1.76± 0.21 |  | 3.48 ± 0.50 | 0.71 ± 0.07 |  |  | Group 2 (RIF +INH) |  |  |  |  |
|  |  |  |  |  |  |  |  |  | Group 3 (RIF +INH+ 100 mg/ kg) | 254.32 ± 11.69 | 73. 69 ± 3.39 | 365.59 ± 8.11 | 1.09 ± 0.09 |  | 4.97 ± 0.41 | 0.94 ± 0.05 |  |  | Group 3 (RIF +INH+ 100 mg/ kg) |  |  |  |  |
|  |  |  |  |  |  |  |  |  | Group 4 RIF +INH+ 200mg/ kg) | 235.60 ± 14.22 | 66.84 ± 4.22 | 304.47 ± 11.52 | 0.90 ± 0.14 |  | 6.59 ± 0.74 | 1.02 ± 0.05 |  |  | Group 4 RIF +INH+ 200mg/ kg) |  |  |  |  |
|  |  |  |  |  |  |  |  |  | Group 5 RIF +INH+ 400 mg/ kg) | 193.66 ± 6.96 | 52.63 ± 4.99 | 248.64 ± 12.49 | 0.61 ± 0.11 |  | 7.64 ± 0.38 | 1.18 ± 0.12 |  |  | Group 5 RIF +INH+ 400 mg/ kg) |  |  |  |  |
|  |  |  |  |  |  |  |  |  | Group 6 (RIF+ INH + SIL) | 168. 36 ± 7.01 | 46. 80 ± 4.14 | 239.22 ± 7.50 | 1.03 ± 0.10 |  | 7.23 ± 0.28 | 1.29 ± 0.07 |  |  | Group 6 (RIF+ INH + SIL) |  |  |  | Balakrishnan S, Khurana BS, Singh A, Kaliappan I, Dubey GP. Hepatoprotective effect of hydroalcoholic extract of Cissampelos pareira against rifampicin and isoniazid induced hepatotoxicity. Cont J Food Sci Technol. 2012;6(1):30-5. |
| *Cissus quadrangularis L.* | stem | India | Wistar albino rats of either sex | Rifampicin | 54 mg/kg | n=6 | 500 mg/kg | 30 days | Group I (normal) | 53.63 ± 3.06 | 45. 22 ± 2.20 | 133. 2 ± 3.60 | 0.78 ± 0.04 | 0.25 ± 0.03 |  |  |  |  | Group I (normal) | 6.97 ± 1.06 |  | 12.76 ± 1.36 |  |
|  |  |  |  |  |  |  |  |  | Group II (RIF) | 90.84 ± 3.39 | 97.62 ± 3.98 | 267.7 ± 10.10 | 1.08 ± 0.10 | 0.52 ± 0.03 |  |  |  |  | Group II (RIF) | 3.51 ± 0.52 |  | 6.01 ± 1.09 |  |
|  |  |  |  |  |  |  |  |  | Group III (CQ 500 mg/kg) | 75. 68 ± 2.63 | 71.02 ± 3.71 | 187.5 ± 4.14 | 0.84 ± 0.02 | 0.37 ± 0.02 |  |  |  |  | Group III (CQ 500 mg/kg) | 6.39 ± 0.50 |  | 10.13 ± 0.44 |  |
|  |  |  |  |  |  |  |  |  | Group IV (SIL+ RIF) | 69.98 ± 2.40 | 64.01 ± 2.66 | 61.7 ± 4.71 | 0.79 ± 0.01 | 0.27 ± 0.01 |  |  |  |  | Group IV (SIL+ RIF) | 6.48 ± 0.76 |  | 11.00 ± 1.37 | Swamy AV, Kulkarni RV, Koti BC, Gadad PC, Thippeswamy AH, Gore A. Hepatoprotective effect of Cissus quadrangularis stem extract against rifampicin-induced hepatotoxicity in rats. Indian Journal of Pharmaceutical Sciences. 2012 Mar;74(2):183. |
| *Cnidoscolus chayamansa Mc.* | leaves and stem | Mexico | Male Balb/C mice | Isoniazid, rifampicin and pyrazinamide | 50, 50 and 100 mg/kg | n= 6 | 200 and 400 mg/kg, | 85 days | Group 1 (Healthy) | 195 ± 12.10 | 124 ± 15.8 | 70. 33± 13.56 |  |  |  |  |  |  | Group 1 (Healthy) |  | 557.2 ± 21.1 | 308. 46 ± 32.1 |  |
|  |  |  |  |  |  |  |  |  | Group 2 (anti-tb) | 329.7 ± 15.83 | 174 ± 17.5 | 182.3 ± 14.16 |  |  |  |  |  |  | Group 2 (anti-tb) |  | 895.3 ± 17.7 | 465.67 ± 27.49 |  |
|  |  |  |  |  |  |  |  |  | Group 3 (anti-tb + SIL) | 308.5 ± 13.62 | 145.5 ± 3.82 | 186 ± 12.72 |  |  |  |  |  |  | Group 3 (anti-tb + SIL) |  | 555.1 ± 20.8 | 334.33 ± 26 |  |
|  |  |  |  |  |  |  |  |  | Group 4 (anti-tb + CNIL) 200 mg/kg | 302. 7 ± 14.13 | 143. 6 ± 14.50 | 184.6 ± 15.3 |  |  |  |  |  |  | Group 4 (anti-tb + CNIL) 200 mg/kg |  | 544.8 ± 17.3 | 213.93 ± 38.6 |  |
|  |  |  |  |  |  |  |  |  | Group 5(anti-tb + CNIL) 400 mg/kg | 218 ± 17.31 | 158.75 ± 13.91 | 175 ± 14.2 |  |  |  |  |  |  | Group 5(anti-tb + CNIL) 400 mg/kg |  | 552. 55 ± 20.22 | 303.48 ± 30.96 | Pérez-González MZ, Macías-Rubalcava ML, Hernández-Ortega S, Siordia-Reyes AG, Jiménez-Arellanes MA. Additional compounds and the therapeutic potential of Cnidoscoluschayamansa (McVaugh) against hepatotoxicity induced by antitubercular drugs. Biomedicine & Pharmacotherapy. 2019 Sep 1; 117:109140. |
| *Crassocephalum vitellinum Benth.* | leaves | Uganda | Wistar albino rats (Rattus norvegicus) of both sex | Rifampicin | 10mg/kg | n=6 | 200,400 and 800 mg/kg | 35 days | Group I (normal) | 117 ± 1.36 | 50.95 ± 1.55 | 70.12 ± 1.72 |  |  |  |  |  |  | Group I (normal) |  |  |  |  |
|  |  |  |  |  |  |  |  |  | Group II (RIF) | 425.9 ± 4.45 | 262.2 ± 1.92 | 173.8 ± 2.27 |  |  |  |  |  |  | Group II (RIF) |  |  |  |  |
|  |  |  |  |  |  |  |  |  | Group III (RIF +200 mg/kg CV) | 375.4 ± 1.98 | 193.3 ± 1.18 | 145.3 ± 1.33 |  |  |  |  |  |  | Group III (RIF +200 mg/kg CV) |  |  |  |  |
|  |  |  |  |  |  |  |  |  | Group IV (RIF + 400 mg/kg CV) | 222.4 ± 0.91 | 136.2 ± 1.62 | 121.7 ± 1.12 |  |  |  |  |  |  | Group IV (RIF + 400 mg/kg CV) |  |  |  |  |
|  |  |  |  |  |  |  |  |  | Group V (RIF +200 mg/kg CV) | 135.3 ± 1.09 | 71.5 ± 1.92 | 94.7 ± 0.57 |  |  |  |  |  |  | Group V (RIF +200 mg/kg CV) |  |  |  |  |
|  |  |  |  |  |  |  |  |  | Group VI (SIL + RIF) | 125 ± 1.81 | 60.98 ± 0.79 | 81.28 ± 1.95 |  |  |  |  |  |  | Group VI (SIL + RIF) |  |  |  | Kiyimba K, Ayikobua ET, Mwandah DC, Obakiro SB. Assessing the protective effect of Crassocephalumvitellinum against Rifampicin-induced hepatotoxicity in Wistar rats. African Health Sciences. 2022 Apr 29;22(1):352-60. |
| *Ensete ventricosum Welw.* | corm | Wanci woreda | Swiss albino mice | Isoniazid and rifampicin | 75 and 150 mg/kg | n= 6 | 200 and 400 mg/kg, | 30 days | Group I (normal) | 53.67 ± 4.40 | 34.66 ± 3.42 | 98.33 ± 2.49 | 0.12 ± 0.01 |  |  |  |  |  | Group I (normal) |  |  |  |  |
|  |  |  |  |  |  |  |  |  | Group II (INH+ RIF) | 152.83 ± 3.84 | 123.67 ± 3.25 | 171.00 ± 4.69 | 0.28 ± 0.02 |  |  |  |  |  | Group II (INH+ RIF) |  |  |  |  |
|  |  |  |  |  |  |  |  |  | Group III (200mg/kg) | 87.50 ± 4.96 | 90.00 ± 4.89 | 120. 83 ± 3.75 | 0.21 ± 0.03 |  |  |  |  |  | Group III (200mg/kg) |  |  |  |  |
|  |  |  |  |  |  |  |  |  | Group IV (400 mg/kg) | 75.33 ± 3.21 | 53.17 ± 4.95 | 108.00 ± 4.09 | 0.19 ± 0.03 |  |  |  |  |  | Group IV (400 mg/kg) |  |  |  |  |
|  |  |  |  |  |  |  |  |  | Group V (silymarin control) | 62.17 ± 1.62 | 40.16 ± 3.09 | 106. 33 ± 2.40 | 0.13 ± 0.01 |  |  |  |  |  | Group V (silymarin control) |  |  |  | Dubiwak AD, Damtew TW, Senbetu MW, Yewhalaw D, Asere TG, Nemo G, Baye MF. Hepatoprotective effect of corm of Enseteventricosum (welw.) cheesman extract against isoniazid and rifampicin induced hepatotoxicity in Swiss albino mice. Journal of Toxicology. 2021 Aug 13;2021. |
| *Ficus carica L.* | leaves | India | Wistar rats of either sex | rifampicin | 50 mg/kg | n=6 | 200mg/kg | 60 days | Group I (normal) | 181 ±1.549 | 63 ± 2.800 |  | 0.77 ± 0.019 |  |  |  |  |  | Group I (normal) |  |  |  |  |
|  |  |  |  |  |  |  |  |  | Group II (RIF) |  | 88 ± 1.048 |  | 0.94 ± 0.029 |  |  |  |  |  | Group II (RIF) |  |  |  |  |
|  |  |  |  |  |  |  |  |  | Group III (Extract) | 200 ± 3.386 | 64 ± 2.316 |  | 0.78 ± 0.009 |  |  |  |  |  | Group III (Extract) |  |  |  |  |
|  |  |  |  |  |  |  |  |  | Group IV (RIF+ extract) | 184 ± 2.16 | 66 ± 2.44 |  | 0.80 ± 0.019 |  |  |  |  |  | Group IV (RIF+ extract) |  |  |  | Gond NY, Khadabadi SS. Hepatoprotective activity of Ficus carica leaf extract on rifampicin-induced hepatic damage in rats. Indian journal of pharmaceutical sciences. 2008 May;70(3):364. |
| *Alpinia officinarum H.* | rhizome | India | Female Sprague Dawley rats | Isoniazid and rifampicin | 50 mg/kg | n=5 | 200 and 400 mg/kg | 21 days | Group I (normal) | 38.64 ± 0.90 | 43.66 ± 2.8 | 90.9 ± 4.32 |  |  | 7.74 ± 0.29 | 4.78 ± 0.09 |  |  | Group I (normal) | 1.572 ± 0.03 | 46.7 ± 1.13 | 5.67 ± 0.04 |  |
|  |  |  |  |  |  |  |  |  | Group II (toxic) | 92.88 ± 3.70 | 98.66 ± 4.9 | 216.48 ± 4.21 |  |  | 4.4 ± 0.21 | 2.46 ± 0.40 |  |  | Group II (toxic) | 0.59 ± 0.05 | 17.7 ± 1.11 | 1.24 ± 0.06 |  |
|  |  |  |  |  |  |  |  |  | Group III (200mg/kg) | 74.18 ± 3.48 | 87.7 ± 3.7 | 202.96 ± 4.29 |  |  | 5.46 ± 0.28 | 3.34 ± 0.27 |  |  | Group III (200mg/kg) | 0.84 ± 0.03 | 30.4 ± 1.04 | 3.63 ± 0.04 |  |
|  |  |  |  |  |  |  |  |  | Group IV (400 mg/kg) | 59.18 ± 5.71 | 75.28 ± 4.1 | 176.3 ± 2.46 |  |  | 6.32 ± 0.35 | 4.12 ± 0.12 |  |  | Group IV (400 mg/kg) | 0.98 ± 0.06 | 35.4 ± 1.18 | 4.01 ± 0.02 |  |
|  |  |  |  |  |  |  |  |  | Group V (STD) | 50.18 ± 4.36 | 57.68 ± 3.7 | 155.82 ± 1.86 |  |  | 6.98 ± 0.31 | 4.48 ± 0.13 |  |  | Group V (STD) | 1.39 ± 0.02 | 41.3 ± 1.24 | 4.89 ± 0.04 | Eram S, Mujahid M, Bagga P, Ahsan F, Rahman MA. Hepatoprotective evaluation of Galanga (Alpinia Officinarum) rhizome extract against antitubercular drugs induced hepatotoxicity in rats. Journal of Herbs, Spices & Medicinal Plants. 2020 Apr 2;26(2):113-25. |
| *Garcinia kola Heckel.* | seeds | Nigeria | albino rats of either sex | Isoniazid and rifampicin | 7.5 mg/kg | 5 | 40, 60 and 80 mg/kg | 35 days | Group I (normal) | 437.75 ± 13.16 | 7.35 ± 2.85 | 89.94 ± 11.41 | 0.169 ± 0.033 |  |  |  |  |  | Group I (normal) | 0.220 ± 0.075 | 15.83 ± 0.05 | 1.570 ± 0.188 |  |
|  |  |  |  |  |  |  |  |  | Group II (G. kola only) | 477.00 ± 13.30 | 10.84 ± 1.30 | 69.00 ± 9.91 | 0.226 ± 0.010 |  |  |  |  |  | Group II (G. kola only) | 0.377 ± 0.208 | 27.50 ± 2.78 | 1.157 ± 0.523 |  |
|  |  |  |  |  |  |  |  |  | Group III (INH + RIF) | 535.20 ± 26.75 | 17.56 ± 3.35 | 116.33 ± 13.34 | 0.380 ± 0.025 |  |  |  |  |  | Group III (INH + RIF) | 0.109 ± 0.097 | 11.53 ± 2.57 | 0.568 ± 0.245 |  |
|  |  |  |  |  |  |  |  |  | Group IV (INH + RIF+ G. kola 40 mg/kg) | 434.80 ± 15.14 | 7.04 ± 1.75 | 63.48 ± 9.03 | 0.139 ± 0.023 |  |  |  |  |  | Group IV (INH + RIF+ G. kola 40 mg/kg) | 0.388 ± 0.145 | 28.38 ± 1.12 | 2.830 ± 0.496 |  |
|  |  |  |  |  |  |  |  |  | Group V (INH + RIF+ G. kola 60 mg/kg) | 362.00 ± 20.94 | 6.40 ± 3.09 | 37.26 ± 12.77 | 0.127 ± 0.009 |  |  |  |  |  | Group V (INH + RIF+ G. kola 60 mg/kg) | 0.303 ± 0.123 | 22.91 ± 2.99 | 1.812 ± 0.305 |  |
|  |  |  |  |  |  |  |  |  | Group VI (INH + RIF+ G. kola 80 mg/kg) | 412.60 ± 11.63 | 7.84 ± 1.80 | 57.41 ± 3.07 | 0.125 ± 0.025 |  |  |  |  |  | Group VI (INH + RIF+ G. kola 80 mg/kg) | 0.460 ± 0.215 | 19.53 ± 1.41 | 1.466 ± 1.071 | Ogono T, Taiwo OE, Usman K, Adeyemi D, Martins E, Obuotor FA, Osoniyi O. An investigation of the hepatoprotective potential of Garcinia kola seed extract in an anti-tubercular treatment model. J. Med. Plants Res. 2014 Nov 26; 8:1156-63. |
| *Allium sativum L.* | whole herb | Nigeria | rats | Rifampicin +Isoniazid + Pyrazinamide +Ethambutol | 20mg/ml | 4 | 28.5, 42.8 and 57.1 mg/kg | 28 | Group I (negative + anti-TB drugs) | 67.5 ± 3.53 | 61.5 ± 2.12 | 106.5 ± 6.36 |  |  |  |  |  |  | Group I (negative + anti-TB drugs) |  |  |  |  |
|  |  |  |  |  |  |  |  |  | Group II (anti-TB drugs + 28.5 mg/kg) | 51.5 ± 2.12 | 51.5 ± 0.70 | 54.5 ± 7.77 |  |  |  |  |  |  | Group II (anti-TB drugs + 28.5 mg/kg) |  |  |  |  |
|  |  |  |  |  |  |  |  |  | Group III (anti-TB drugs + 42.8 mg/kg | 34.0 ± 5.65 | 41.0 ± 1.41 | 47.5 ± 2.12 |  |  |  |  |  |  | Group III (anti-TB drugs + 42.8 mg/kg |  |  |  |  |
|  |  |  |  |  |  |  |  |  | Group IV anti-TB drugs + 57.1 mg/kg) | 27.0 ± 1.40 | 29.5 ± 0.76 | 43.0 ± 5.65 |  |  |  |  |  |  | Group IV anti-TB drugs + 57.1 mg/kg) |  |  |  |  |
|  |  |  |  |  |  |  |  |  | Group v (positive control) | 11.0 ± 1.41 | 9.50 ± 2.10 | 31.5 ± 3.53 |  |  |  |  |  |  | Group v (positive control) |  |  |  | Nasiru A, Hafsat IG, Mohammad MM, Sabo AA. Hepatoprotective effect of garlic homogenate co-administered with anti-tuberculosis drugs in ratliver enzymes. International Journal of Bioscience, Biochemistry and Bioinformatics. 2012 Sep 1;2(5):354. |
| *Hedyotis corymbose L.* | whole plant | Bangladesh | Adult male rats (Rattus novergicus: Sprague-Dawley strain) | Isoniazid and rifampicin | 50 mg/kg | 6 | 500 mg/kg | 28 | Group I (control) | 23.83 ± 4.31 | 17.33 ± 3.39 | 144.50 ± 5.28 | 0.33 ± 0.15 |  | 6.26 ± 0.29 | 4.35 ± 0.44 |  |  | Group I (control) |  |  |  |  |
|  |  |  |  |  |  |  |  |  | Group II (STD) | 31.152 ± 4.11 | 21.23 ± 3.12 | 151. 25 ± 6.78 | 0.31 ± 0.04 |  | 6.08 ± 0.12 | 4.32 ± 0.09 |  |  | Group II (STD) |  |  |  |  |
|  |  |  |  |  |  |  |  |  | Group III (INH + RIF) | 78.00 ± 5.55 | 56.67 ± 3.98 | 177.83 ± 17.07 | 1.22 ± 0.16 |  | 4. 85 ± 0.62 | 3.68 ± 0.31 |  |  | Group III (INH + RIF) |  |  |  |  |
|  |  |  |  |  |  |  |  |  | Group IV (INH + RIF+ HC) | 52.33 ± 9.71 | 33.50 ± 7.87 | 163.17 ± 11.87 | 0.55 ± 0.19 |  | 5.61 ± 0.41 | 4.09 ± 0.12 |  |  | Group IV (INH + RIF+ HC) |  |  |  | Lina SM, Ashab I, Bhuiya MS, Shahriar M. Hepatoprotective activity of Hedyotiscorymbosa (Linn.) Lam. extract against anti-tubercular drug induced hepatic damage in Sprague-Dawley rats. Bangladesh Pharm J. 2018; 21:131-8. |
| *Hibiscus vitifolius L.* | roots | India. | Healthy Wistar albino rats and adult Swiss albino mice | Isoniazid, rifampicin and pyrazinamide | 7.5, 10 and 35 mg/kg | 6 | 400 mg/kg | 45 | Control group | 72.5 ± 0.57 | 46.8 ± 0.67 | 322. 00 ± 2.30 | 0.33 ± 0.018 | 0.16 ± 0.007 | 7.66 ± 0.113 | 3.21 ± 0.11 |  |  | Control group |  | 14. 987 ± 0.177 | 3.586 ± 0.272 |  |
|  |  |  |  |  |  |  |  |  | HPTM | 462.78 ± 2.99 | 326.00 ± 3.53 | 450. 65 ± 2.17 | 2.51 ± 0.022 | 1.43 ± 09.03 | 4.44 ± 0.056 | 1.84 ± 0.24 |  |  | HPTM |  | 7.771 ± 0.102 | 0.893 ± 0.015 |  |
|  |  |  |  |  |  |  |  |  | Petroleum ether 400 + HPTM | 371. 42 ± 2.66 | 300.46 ± 2.91 | 411.53 ± 3.48 | 2.19 ± 0.018 | 1.29 ± 0.09 | 4.67 ± 0.52 | 2.08 ± 0.032 |  |  | Petroleum ether 400 + HPTM |  | 8.213 ± 0.173 | 0.986 ± 0.0234 |  |
|  |  |  |  |  |  |  |  |  | Chloroform 400 + HPTM | 361. 62 ± 2. 14 | 299. 78 ± 3.46 | 401.08 ± 3.991 | 2.03 ± 0.018 | 1.13 ± 0.029 | 5.18 ± 0.036 | 2.12 ± 0.086 |  |  | Chloroform 400 + HPTM |  | 9.723 ± 0.286 | 1.591 ± 0.096 |  |
|  |  |  |  |  |  |  |  |  | Methanolic 400 + HPTM | 74.76 ± 4.24 | 48.44 ± 3.19 | 322.39 ± 9.10 | 0.35 ± 0.013 | 0.18 ± 0.01 | 7.54 ± 1.81 | 3.09 ± 0.07 |  |  | Methanolic 400 + HPTM |  | 12.977 ± 0.571 | 3.404 ± 0.017 |  |
|  |  |  |  |  |  |  |  |  | Aqueous 400 + HPTM | 94.8 ± 5.07 | 60. 5 ± 3.11 | 336.00 ± 3.307 | 0.58 ± 0.015 | 0.33 ± 0.042 | 5.25 ± 0.30 | 2.04 ± 0.151 |  |  | Aqueous 400 + HPTM |  | 10.123 ± 0.489 | 1.921 ± 1.68 |  |
|  |  |  |  |  |  |  |  |  | Silymarin 100 + HPTM | 73.31 ± 1.95 | 48.01 ± 2.72 | 322.84 ± 2.31 | 0.29 ± 0.003 | 0.16 ± 0.004 | 6.99 ± 0.26 | 2.28 ± 0.044 |  |  | Silymarin 100 + HPTM |  | 13.231 ± 0.027 | 3.497 ± 0.112 | Samuel AJ, Mohan S, Chellappan DK, Kalusalingam A, Ariamuthu S. Hibiscus vitifolius (Linn.) root extracts show potent protective action against anti-tubercular drug induced hepatotoxicity. Journal of ethnopharmacology. 2012 May 7;141(1):396-402. |
| *Ipomoea carnea L.* | leaves | India | Healthy albino Wistar rats | Isoniazid, rifampicin and pyrazinamide | 7.5, 10 and 35 mg/kg | 6 | 125, 250 and 500 mg/kg | 35 | Control | 58.2 ± 4.47 | 42.60 ± 3.90 | 54.14 ± 3.88 | 0.76 ± 0.18 |  |  |  |  | 0.32 ± 0.02 | Control | 6.21 ± 0.21 | 49.51 ± 4.52 | 24.13 ± 2.72 |  |
|  |  |  |  |  |  |  |  |  | R+I+P | 172.28 ± 9.20 | 147.21 ± 5.01 | 162.21 ± 8.20 | 3.13 ± 0.14 |  |  |  |  | 1.21 ± 0.07 | R+I+P | 3.41 ± 0.28 | 31.41 ± 2.37 | 12.73 ± 1.42 |  |
|  |  |  |  |  |  |  |  |  | ICE-125 (R+I+P) | 156.70 ± 7.20 | 142.61 ± 4.80 | 144.21 ± 7.10 | 2.61 ± 0.12 |  |  |  |  | 1.04 ± 0.06 | ICE-125 (R+I+P) | 4.77 ± 0.31 | 33.52 ± 2.47 | 13.21 ± 1.01 |  |
|  |  |  |  |  |  |  |  |  | ICE-250 (R+I+P) | 141.20 ± 7.10 | 131.80 ± 4.20 | 131.28 ± 6.20 | 2.41 ± 0.16 |  |  |  |  | 0.97 ± 0.05 | ICE-250 (R+I+P) | 5.01 ± 0.32 | 37.23 ± 3.21 | 15.44 ± 2.23 |  |
|  |  |  |  |  |  |  |  |  | ICE- 500 (R+I+P) | 102.79 ± 6.40 | 98.21 ± 3.90 | 92.08 ± 4.90 | 1.62 ± 0.17 |  |  |  |  | 0.63 ± 0.05 | ICE- 500 (R+I+P) | 5.96 ± 0.39 | 41.50 ± 3.25 | 18.61 ± 2.11 |  |
|  |  |  |  |  |  |  |  |  | SYL-100 (R+I+P) | 89.21 ± 5.80 | 68.21 ± 2.90 | 72.32 ± 3.20 | 1.02 ± 0.11 |  |  |  |  | 0.48 ± 0.03 | SYL-100 (R+I+P) | 6.03 ± 0.41 | 44.80 ± 2.21 | 21.32 ± 2.12 | Suresh K, Singh RK. Anti-hepatotoxic and antioxidant influence of Ipomoea carnea against anti-tubercular drugs induced acute hepatopathy in experimental rodents. J Coast Life Med. 2013; 1:293-9. |
| *Ixora pavetta A.* | leaves | India | Healthy male Wistar rats | Isoniazid and rifampicin | 100 mg/kg | 6 | 200 and 400 mg/kg | 21 | Group I (normal) | 169 ± 3.6 | 178 ± 5.0 | 177.2 ± 3.77 | 0.97 ± 0.046 | 0.78 ± 0.037 | 7.6 ± 0.09 |  |  |  | Group I (normal) |  |  |  |  |
|  |  |  |  |  |  |  |  |  | Group II (Toxic group) | 324 ± 6.4 | 309 ± 4.8 | 319.2 ± 6.9 | 2.02 ± 0.08 | 1.98 ± 0.05 | 5.67 ± 0.03 |  |  |  | Group II (Toxic group) |  |  |  |  |
|  |  |  |  |  |  |  |  |  | Group III (SYL group) | 196 ± 4.5 | 197 ± 3.2 | 203.1 ± 4.8 | 1.13 ± 0.05 | 0.97 ± 0.05 | 7.41 ± 0.05 |  |  |  | Group III (SYL group) |  |  |  |  |
|  |  |  |  |  |  |  |  |  | Group IV (100mg) | 292 ± 7.4 | 276 ± 58.6 | 239.3 ± 4.8 | 1.66 ± 0.06 | 1.45 ± 0.07 | 6.11 ± 0.06 |  |  |  | Group IV (100mg) |  |  |  |  |
|  |  |  |  |  |  |  |  |  | Group V (400 mg) | 225 ± 5.1 | 210.3 ± 4.3 | 210.6 ± 4.15 | 1.18 ± 0.06 | 1.05 ± 0.05 | 7.11 ± 0.1 |  |  |  | Group V (400 mg) |  |  |  | Reddy GJ, Reddy VP, Sreepavani M, Rajaram C, Kumar SN, Kanhere RS. Evaluation of hepatoprotective potential of ethanolic extract of Ixora pavettaagainst isoniazid and rifampicin induced hepatotoxicity in rats. Drug invention today. 2013 Sep 1;5(3):201-6. |
| *Jasminum grandiflorum L.* | leaves | India | Albino Wistar rats | Isoniazid | 54 | 6 | 200 | 30 | Group I (normal) | 17.67 ± 10.70 | 31.17 ± 14.46 |  |  |  |  |  |  |  | Group I (normal) |  |  |  |  |
|  |  |  |  |  |  |  |  |  | Group II (INH) | 323.5 ± 7.719 | 79.17 ± 4.658 |  |  |  |  |  |  |  | Group II (INH) |  |  |  |  |
|  |  |  |  |  |  |  |  |  | Group III (JG 200mg/kg) | 271.2 ± 11.24 | 64.5 ± 5.353 |  |  |  |  |  |  |  | Group III (JG 200mg/kg) |  |  |  |  |
|  |  |  |  |  |  |  |  |  | Group IV (SYL STD) | 244.2 ± 11.6 | 52.33 ± 1.687 |  |  |  |  |  |  |  | Group IV (SYL STD) |  |  |  | Dhamal N, Patel M, Pawar S. Evaluation of Jasminum grandiflorum for hepatoprotective activity in isoniazid induced liver damage. International Journal of Pharmaceutical Sciences and Research. 2012 Aug 1;3(8):2568. |
| *Lawsonia inermis L.* | leaves | India | Male rats (Wistar strain) | Isoniazid and rifampicin | 50 | 6 | 100 | 15 | Group I (normal) | 82.1 0 ± 8.40 | 54.96 ± 13.55 | 103.70 ± 7.52 | 0.558 ± 0.10 |  |  |  |  |  | Group I (normal) |  |  |  |  |
|  |  |  |  |  |  |  |  |  | Group II (Normal + Lawsone (100mg/kg) | 92.10 ± 9.40 | 74.96 ± 10.23 | 123.73 ± 5.52 | 0.498 ± 0.12 |  |  |  |  |  | Group II (Normal + Lawsone (100mg/kg) |  |  |  |  |
|  |  |  |  |  |  |  |  |  | Group III (RMP- INH) | 492.40 ± 156.30 | 208.10 ± 33.84 | 376. 50 ± 70.14 | 2.402 ± 0.65 |  |  |  |  |  | Group III (RMP- INH) |  |  |  |  |
|  |  |  |  |  |  |  |  |  | Group IV (RMP+ INH + SYL) | 112.80 ± 43.33 | 83.58 ± 22.07 | 141.40 ± 34.33 | 0.553 ± 0.18 |  |  |  |  |  | Group IV (RMP+ INH + SYL) |  |  |  |  |
|  |  |  |  |  |  |  |  |  | Group V (RMP-INH + Lawsone 50mg/kg) | 141.50 ± 42.12 | 84.88 ± 25.45 | 190.50 ± 55.58 | 0.761 ± 0.24 |  |  |  |  |  | Group V (RMP-INH + Lawsone 50mg/kg) |  |  |  |  |
|  |  |  |  |  |  |  |  |  | Group VI (RMP-INH + Lawsone (100 mg/kg) | 138.90 ± 39.31 | 52.09 ± 3.17 | 140.30 ± 14.19 | 0.470 ± 0.07 |  |  |  |  |  | Group VI (RMP-INH + Lawsone (100 mg/kg) |  |  |  | Darvin SS, Esakkimuthu S, Toppo E, Balakrishna K, Paulraj MG, Pandikumar P, Ignacimuthu S, Al-Dhabi NA. Hepatoprotective effect of lawsone on rifampicin-isoniazid induced hepatotoxicity in in vitro and in vivo models. Environmental Toxicology and Pharmacology. 2018 Jul 1; 61:87-94. |
| *Maytenus royleanus Wall.* | leaves | Pakistan | Adult BALB/c male mice | Rifampicin +Isoniazid + Pyrazinamide +Ethambutol | 13.5, 6.75,36.0 and 24.8 | 6 | 200 and 400 | 15 | Group I (control) | 94.17 ± 3.41 | 70.46 ± 2.35 | 142.87 ± 4.65 |  |  |  |  |  |  | Group I (control) | 40.82 ± 2.07 | 1.93 ± 0.16 | 9.23 ± 0.21 |  |
|  |  |  |  |  |  |  |  |  | Group II (RIPE) | 204.9 ± 6.11 | 211.2 ± 5.13 | 306.47 ± 6.68 |  |  |  |  |  |  | Group II (RIPE) | 32.18 ± 1.88 | 0.54 ± 0.11 | 4.15 ± 0.23 |  |
|  |  |  |  |  |  |  |  |  | Group III (MEM 200 mg/kg) + RIPE | 170.29 ± 6. 38 | 169.9 ± 4.28 | 252.14 ± 5.31 |  |  |  |  |  |  | Group III (MEM 200 mg/kg) + RIPE | 35.51 ± 2.13 | 1.83 ± 0.10 | 6.54 ± 0.17 |  |
|  |  |  |  |  |  |  |  |  | Group IV (MEM 400 mg/kg) + RIPE | 121.15 ± 4.28 | 123.0 ± 2.24 | 172.13 ± 3.20 |  |  |  |  |  |  | Group IV (MEM 400 mg/kg) + RIPE | 37.71 ± 2.26 | 1.89 ± 0.12 | 8.35 ± 0.19 |  |
|  |  |  |  |  |  |  |  |  | Group V (Vit B6 + RIPE) | 109.62 ± 4.4 | 98.13 ± 3.13 | 161.15 ± 3.98 |  |  |  |  |  |  | Group V (Vit B6 + RIPE) | 36.51 ± 2.05 | 2.08 ± 0.12 | 8.12 ± 0.24 |  |
|  |  |  |  |  |  |  |  |  | Group VI (Vit B6 alone) | 98.26 ± 4.89 | 68.14 ± 2.81 | 143.66 ± 3.63 |  |  |  |  |  |  | Group VI (Vit B6 alone) | 41.46 ± 2.20 | 1.88 ± 0.14 | 11.32 ± 0.18 |  |
|  |  |  |  |  |  |  |  |  | Group VII (MEM 400 mg/kg) alone | 93.73 ± 4.36 | 69.58 ± 2.63 | 144.35 ± 5.87 |  |  |  |  |  |  | Group VII (MEM 400 mg/kg) alone | 42.39 ± 1.67 | 2.42 ± 0.17 | 10.36 ± 0.23 | Shabbir M, Afsar T, Razak S, Almajwal A, Khan MR. Phytochemical analysis and Evaluation of hepatoprotective effect of *Maytenus royleanus* leaves extract against anti-tuberculosis drug induced liver injury in mice. Lipids in health and disease. 2020 Dec;19:1-5. |
| *Nigella sativa L.* | roots | Islamabad | adult male Albino mice | Pyrazinamide | 20 | 17 | 500 | 42 | Group I (A) | 48.3± 15.011 | 27.40 ± 7.29 |  |  |  |  |  |  |  | Group I (A) |  |  |  |  |
|  |  |  |  |  |  |  |  |  | Group II (B) | 103.0 ± 28.063 | 82.80 ± 21.88 |  |  |  |  |  |  |  | Group II (B) |  |  |  |  |
|  |  |  |  |  |  |  |  |  | Group III (c) | 67.40 ± 12.72 | 38.40 ± 7.891 |  |  |  |  |  |  |  | Group III (c ) |  |  |  |  |
|  |  |  |  |  |  |  |  |  | Group IV (D) | 54.40 ± 14.68 | 36.70 ± 8.17 |  |  |  |  |  |  |  | Group IV (D) |  |  |  | Hafeez A, Waheed A, Yousaf N. Role of Nigella Sativa Seeds on Pyrazinamide Induced Hepatotoxicity. Journal of Islamic International Medical College (JIIMC). 2019 Mar 1;14(1):18-22. |
| *Nyctanthes arbor-tristis L.* | leaves | India | Albino Wistar male rats | Isoniazid, rifampicin and pyrazinamide | 7.5, 10 and 35 | 6 | 125 and 250 | 30 | Group I | 121.59 ± 3.5 | 42.60 ± 2.1 | 141.50 ± 2.1 | 0.58 ± 0.06 |  |  |  |  | 1.36 ± 0.12 | Group I | 1.46 ± 0.12 | 53.56 ± 2.2 | 22.7 ±1.2 |  |
|  |  |  |  |  |  |  |  |  | Group II | 242.64 ± 4.7 | 123.43 ± 2.8 | 289.60 ± 2.8 | 1.20 ± 0.03 |  |  |  |  | 0.41 ± 0.01 | Group II | 0.47 ± 0.01 | 28.75 ± 2.1 | 8.9 ± 1.1 |  |
|  |  |  |  |  |  |  |  |  | Group III | 228.38 ± 3.8 | 114.85 ± 2.8 | 279.70 ± 2.8 | 1.06 ± 0.02 |  |  |  |  | 0.59 ± 0.02 | Group III | 0.72 ± 0.03 | 36.8 ± 2.1 | 13.1 ± 2.1 |  |
|  |  |  |  |  |  |  |  |  | Group IV | 221.10 ± 3.1 | 106. 40 ± 2.8 | 241.40 ± 3.1 | 1.01 ± 0.02 |  |  |  |  | 0.68 ± 0.04 | Group IV | 0.84 ± 0.03 | 41.8 ±3.8 | 16.9 ± 1.5 |  |
|  |  |  |  |  |  |  |  |  | Group V | 150. 50 ± 4.1 | 98.30 ± 3.1 | 202.00 ± 3.2 | 0.73 ± 0.04 |  |  |  |  | 0.81 ± 0.07 | Group V | 0.99 ± 0.06 | 46. 2± 2.1 | 19.9 ± 2.1 |  |
|  |  |  |  |  |  |  |  |  | Group VI | 138.32 ± 3.8 | 83.80 ± 2.6 | 183.34 ± 3.2 | 0.64 ± 0.02 |  |  |  |  | 0.98 ± 0.08 | Group VI | 1.24 ± 0.08 | 53.8 ± 2.5 | 21.9 ± 2.5 | Chaudhary S, Gupta RK, Kumar A, Tarazi H. Hepatoprotective and antioxidant potential of Nyctanthes arbor-tristis L. leaves against antitubercular drugs induced hepatotoxicity. Journal of Pharmacy & Pharmacognosy Research. 2018;6(3):205-15. |
| *Ocimum canum L.* | aerial part | India | Wistar strains of Albino rats | Isoniazid | 54 | 6 | 100 and 200 | 21 | Group I | 12.34 ± 0.7 | 41.7 ± 0.66 | 597. 2 ± 0.8 | 0.98 ± 0.045 |  |  |  |  | 2.53 ± 0.12 | Group I | 44.4 ± 0.31 |  | 3.01 ± 0.02 |  |
|  |  |  |  |  |  |  |  |  | Group II | 201.7 ± 0.076 | 312 ± 0.99 | 997.1 ± 0.034 | 4.1 ± 0.56 |  |  |  |  | 5.1 ± 0.237 | Group II | 27 ± 0.33 |  | 0.87 ± 0.66 |  |
|  |  |  |  |  |  |  |  |  | Group III | 104.3 ± 1.22 | 246.71 ± 0.76 | 876.2 ± 0.51 | 3.71 ± 0.098 |  |  |  |  | 4.81 ± 0.44 | Group III | 30.9 ± 0.11 |  | 1.36 ± 0.78 |  |
|  |  |  |  |  |  |  |  |  | Group IV | 43.9 ± 0.64 | 167.7 ± 0.764 | 778.32 ± 0.662 | 2.23 ± 0.673 |  |  |  |  | 3.41 ± 0.72 | Group IV | 40.7 ± 0.12 |  | 1.97 ± 0.34 |  |
|  |  |  |  |  |  |  |  |  | Group V | 10.31 ± 0.7 | 78.31 ± 0.55 | 621.52 ± 0.77 | 1.0 ± 0.007 |  |  |  |  | 2.34 ± 0.48 | Group V | 46.4 ± 0.36 |  | 2.33 ± 0.112 |  |
|  |  |  |  |  |  |  |  |  | Group VI | 32.25 ± 3.16 | 75.30 ± 3.21 | 326.61 ± 2.10 | 1.67 ± 2.01 |  |  |  |  | 5.21 ± 2.12 | Group VI | 32.21 ± 4.12 |  | 1.23 ± 0.98 | Radhika J, Jothi G, Nivethetha M, Vijayaroslin L, Yuvarani S. Hepatoprotective Efficacy of Ocimum Canum Sims. on Isoniazid Induced Hepatotoxicity. |
| *Otostegia integrifolia Benth.* | leaves | Ethiopia | male Swiss albino mice | Isoniazid, rifampicin | 100 and 150 | 6 | 200 and 400 | 14 | Group I (normal) | 50.16 ± 15.11 | 35.00 ± 2.92 |  | 0.52 ± 0.1 |  |  |  |  |  | Group I (normal) |  |  |  |  |
|  |  |  |  |  |  |  |  |  | Group II (INH and RIF) | 175. 50 ± 21.64 | 84.00 ± 12.97 |  | 0.92 ± 0.09 |  |  |  |  |  | Group II (INH and RIF) |  |  |  |  |
|  |  |  |  |  |  |  |  |  | Group III (200mg/kg) | 131.17 ± 25.93 | 62.83 ± 6.67 |  | 0.71 ± 0.2 |  |  |  |  |  | Group III (200mg/kg) |  |  |  |  |
|  |  |  |  |  |  |  |  |  | Group IV (400 mg/kg) | 94.83 ± 19.26 | 42.67 ± 5.35 |  | 0.56 ± 0.06 |  |  |  |  |  | Group IV (400 mg/kg) |  |  |  |  |
|  |  |  |  |  |  |  |  |  | Group V (N-acetyl cysteine control) | 76.16 ± 14.70 | 37.01 ± 2.5 |  | 0.54 ± 0.03 |  |  |  |  |  | Group V (N-acetyl cysteine control) |  |  |  | Gugsa E, Molla TS, Bekele T, Dejenie TA. Hepatoprotective effect of hydromethanol extract of Otostegia integrifolia benth leaves in isoniazid and rifampicin induced Swiss albino mice. Metabolism Open. 2023 Dec 1;20:100255. |
| *Polygonatum cirrhifolium wall.* | whole plant | India | Wistar rats of either sex | Isoniazid, rifampicin | 50 | 6 | 200 and 400 | 28 | Group I | 50.06 ± 6.31 | 47.12 ± 4.87 | 141.50 ± 4.02 | 0.29 ± 0.01 |  |  |  |  |  | Group I |  |  |  |  |
|  |  |  |  |  |  |  |  |  | Group II | 80.27 ± 6.04 | 70. 58 ± 5.38 | 284.30 ± 3.14 | 0.67 ± 0.01 |  |  |  |  |  | Group II |  |  |  |  |
|  |  |  |  |  |  |  |  |  | Group III | 41.39 ± 2.03 | 44. 89 ±2.52 | 172.80 ± 3.33 | 0.45 ± 0.02 |  |  |  |  |  | Group III |  |  |  |  |
|  |  |  |  |  |  |  |  |  | Group IV | 26.08 ± 3.59 | 26.63 ± 2.77 | 149.80 ± 2.65 | 0.37 ± 0.01 |  |  |  |  |  | Group IV |  |  |  |  |
|  |  |  |  |  |  |  |  |  | Group V | 24.21 ± 4.59 | 19.32 ± 1.95 | 130.30 ±1.45 | 0.32 ± 0.02 |  |  |  |  |  | Group V |  |  |  | Ghai R, Nagarajan K, Grover P, Kumar V, Goel R, Kaur C, Chauhan R. BIOASSAY GUIDED HEPATOPROTECTIVE ACTIVITY OF POLYGONATUM CIRRHIFOLIUM AGAINST ISONIAZID AND RIFAMPICIN INDUCED HEPATOTOXICITY IN RATS. Indian Drugs. 2023 Jul 1;60(07):07. |
| *Rosmarinus officinalis L.* | aerial parts | Mexico | Male Balb/C mice | Isoniazid, rifampicin and pyrazinamide | 10, 10 and 30 | 8 | 250 | 91 | Group I | 231 ± 11.39 | 146.15 ± 11.02 | 184.61 ± 9.78 |  |  |  |  |  |  | Group I |  | 237.45 ± 12.75 |  |  |
|  |  |  |  |  |  |  |  |  | Group II | 346.50 ± 12.75 | 280.70 ± 13.03 | 198.77 ± 10.05 |  |  |  |  |  |  | Group II |  | 326.29 ± 13.39 |  |  |
|  |  |  |  |  |  |  |  |  | Group III | 247.43 ± 12.62 | 238.46 ± 12.19 | 190.31 ± 11.04 |  |  |  |  |  |  | Group III |  | 332.72 ± 19.19 |  |  |
|  |  |  |  |  |  |  |  |  | Group IV | 247.14 ± 11.00 | 239.23 ± 11.65 | 192.30 ± 10.67 |  |  |  |  |  |  | Group IV |  | 383.90 ± 11.10 |  | Jorge CG, Adelina JA. Nephro-and hepatoprotective effect of Rosmarinus officinalis against damage induced with antiTB drugs using a chronic model. Pharmacognosy Journal. 2021;13(3). |
| *Solanum nigrum L.* | whole plant | Egypt | adult male Wistar albino rats, | Isoniazid, rifampicin | 50 | 8 | 250, 500 and 1000 mg/kg | 28 | Group I (negative) | 157.01 ± 1.87 | 180.40 ± 7.80 | 55.03 ± 3.22 | 2.96 ± 0.15 |  |  |  |  |  | Group I (negative) |  |  |  |  |
|  |  |  |  |  |  |  |  |  | Group II (INH and RIF) | 179.27 ± 0.85 | 245.49 ± 8.78 | 111.76 ± 0.85 | 4.52 ± 0.21 |  |  |  |  |  | Group II (INH and RIF) |  |  |  |  |
|  |  |  |  |  |  |  |  |  | Group III (SYL group) | 162. 78 ± 5.58 | 207.53 ± 4.94 | 56.71 ± 1.13 | 4.16 ± 0.06 |  |  |  |  |  | Group III (SYL group) |  |  |  |  |
|  |  |  |  |  |  |  |  |  | Group IV (INH+ RIF + SNE) 250 mg/kg | 159.8 ± 1.63 | 215.73 ± 4.86 | 70.21 ± 3.58 | 3.36 ± 0.07 |  |  |  |  |  | Group IV (INH+ RIF + SNE) 250 mg/kg |  |  |  |  |
|  |  |  |  |  |  |  |  |  | Group V (INH+ RIF + SNE) 500 mg/kg | 159.28 ± 7.13 | 212.13 ± 5.79 | 55.93 ± 2.48 | 3.23 ±0.13 |  |  |  |  |  | Group V (INH+ RIF + SNE) 500 mg/kg |  |  |  |  |
|  |  |  |  |  |  |  |  |  | Group VI (INH+ RIF + SNE) 1000 mg/kg | 159.17 ± 5.93 | 201.34 ± 7.48 | 55.65 ± 0.37 | 3.01 ± 0.19 |  |  |  |  |  | Group VI (INH+ RIF + SNE) 1000 mg/kg |  |  |  | Fayed AH, Salama AA, Ismaiel IE, Attia TA, Hassan A. Protective Effects OfSolanium Nigrum Methanolic Extract Against Isoniazid/Rifampicin Induced Hepatotoxicity In Rats. Journal of Applied Veterinary Sciences. 2019 Oct 7;4(2):52-61. |
| *Tamarindus indica L.* | fruit | Saudi Arabia | Wistar Albino rats | Isoniazid, rifampicin | 50 and 100 | 6 | 250 and 500 mg/kg | 14 | Group I (normal) | 51.21 ± 6.85 | 44.87 ± 5.17 | 28.70 ± 6.92 | 0.51 ± 0.05 |  |  |  |  |  | Group I (normal) | 0.727 ± 0.12 | 9.05 ± 1.35 | 4.45 ± 0.89 |  |
|  |  |  |  |  |  |  |  |  | Group II (anti-TB drugs) | 113.56 ± 6.61 | 133.15 ± 8.06 | 110.03 ± 12.27 | 1.77 ± 0.10 |  |  |  |  |  | Group II (anti-TB drugs) | 0.327 ± 0.05 | 2.33 ± 0.31 | 1.76 ± 0.06 |  |
|  |  |  |  |  |  |  |  |  | Group III (SLY) | 58.20 ± 5.24 | 49.07 ± 7.88 | 33.40 ± 6.78 | 0.62 ± 0.06 |  |  |  |  |  | Group III (SLY) | 0.690 ± 0.08 | 8.02 ± 1.02 | 4.18 ± 0.41 |  |
|  |  |  |  |  |  |  |  |  | Group IV (250 mg/kg) | 100.08 ± 9.11 | 127.08 ± 7.01 | 95.7 ± 9.09 | 1.72 ± 0.07 |  |  |  |  |  | Group IV (250 mg/kg) | 0.371 ± 0.08 | 2.75 ± 0.09 | 1.84 ± 0.08 |  |
|  |  |  |  |  |  |  |  |  | Group V (500 mg/kg) | 65.69 ± 7.18 | 69.04 ± 6.51 | 65.89 ± 6.16 | 0.96 ± 0.11 |  |  |  |  |  | Group V (500 mg/kg) | 0.646 ± 0.09 | 5.85 ± 0.72 | 3.60 ± 0.31 | Amir M, Khan MA, Ahmad S, Akhtar M, Mujeeb M, Ahmad A, Khan SA, Al-Abbasi FA. Ameliorating effects of Tamarindus indica fruit extract on anti-tubercular drugs induced liver toxicity in rats. Natural product research. 2016 Mar 18;30(6):715-9. |
| *Telfairia occidentalis H.* | fruit | Nigeria | male Wistar albino rats | Isoniazid, rifampicin | 100 | 6 | 125, 250 and 500 mg/kg | 60 | Group I (negative) | 99.0 ± 10.0 | 34.3 ± 4.2 | 37.3 ± 2.5 | 14.1 ± 2.7 |  |  |  |  |  | Group I (negative) |  |  |  |  |
|  |  |  |  |  |  |  |  |  | Group II (INH and RIF) | 124.0 ± 11.1 | 40.0 ± 3.6 | 49.3 ± 4.9 | 17.8 ± 2.0 |  |  |  |  |  | Group II (INH and RIF) |  |  |  |  |
|  |  |  |  |  |  |  |  |  | Group III (INH + RIF + TOPE 125mg/kg) | 79.0 ± 7.4 | 26.0 ± 5.0 | 29.8 ± 4.6 | 8.5 ± 1.0 |  |  |  |  |  | Group III (INH + RIF + TOPE 125mg/kg) |  |  |  |  |
|  |  |  |  |  |  |  |  |  | Group IV (INH + RIF +250 mg/kg) | 76.3 ± 5.5 | 21.7 ± 2.9 | 28.0 ± 2.7 | 8.3 ± 1.5 |  |  |  |  |  | Group IV (INH + RIF +250 mg/kg) |  |  |  |  |
|  |  |  |  |  |  |  |  |  | Group V (INH+ RIF + TOPE 500 mg/kg) | 70.3 ± 9.6 | 19.3 ± 2.1 | 26.3 ± 4.0 | 8.0 ± 0.6 |  |  |  |  |  | Group V (INH+ RIF + TOPE 500 mg/kg) |  |  |  |  |
|  |  |  |  |  |  |  |  |  | Group VI (SYL) | 75.0 ± 5.6 | 21.0 ± 1.7 | 26.0 ± 3.6 | 8.8 ± 0.5 |  |  |  |  |  | Group VI (SYL) |  |  |  |  |
|  |  |  |  |  |  |  |  |  | Group VII (TOPE) alone | 70.7 ± 4.0 | 22.0 ± 1.0 | 21.0 ± 1.0 | 8.9 ± 0.5 |  |  |  |  |  | Group VII (TOPE) alone |  |  |  | Nwidu LL, Oboma YI. Telfairia occidentalis (Cucurbitaceae) pulp extract mitigates rifampicin-isoniazid-induced hepatotoxicity in an in vivo rat model of oxidative stress. Journal of Integrative Medicine. 2019 Jan 1;17(1):46-56. |
| *Trapa natans Var.* | fruit peel | India | Adult Wistar rats | Isoniazid, rifampicin | 50 | 6 | 200 and 400 | 15 | Group I (control) |  |  |  |  |  |  |  |  | 0.52 ± 0.06 | Group I (control) | 0.78 ± 0.08 | 48.32 ± 3.02 | 20.12 ± 1.48 |  |
|  |  |  |  |  |  |  |  |  | Group II (INH and RIF) |  |  |  |  |  |  |  |  | 1.95 ± 0.08 | Group II (INH and RIF) | 0.25 ± 0.05 | 17.84 ± 2.33 | 9.52 ± 0.92 |  |
|  |  |  |  |  |  |  |  |  | Group III (TN 200 mg/kg) + INH + RIF |  |  |  |  |  |  |  |  | 1.03 ± 0.06 | Group III (TN 200 mg/kg) + INH + RIF | 0.51 ± 0.04 | 33.74 ± 3.12 | 13.83 ± 1.17 |  |
|  |  |  |  |  |  |  |  |  | Group IV (TN 400mg/kg) + INH + RIF |  |  |  |  |  |  |  |  | 0.62 ± 0.04 | Group IV (TN 400mg/kg) + INH + RIF | 0.72 ± 0.07 | 42.46 ± 3.48 | 17.76 ± 1.46 |  |
|  |  |  |  |  |  |  |  |  | Group V (SYL) |  |  |  |  |  |  |  |  | 0.56 ± 0.05 | Group V (SYL) | 0.74 ± 0.05 | 46.55 ± 3.86 | 18.87 ± 1.31 | Hussain T, Subaiea GM, Firdous H. Hepatoprotective evaluation of Trapa natans against drug-induced hepatotoxicity of antitubercular agents in rats. Pharmacognosy magazine. 2018 Apr;14(54):180. |
| *Vernonia amygdalina Del.* | leaves | Ethiopia | female SAM | Isoniazid | 75 | 6 | 250 and 375 | 14 | Group I (sterile saline) | 114.0 ± 6.52 | 29.80 ± 4.45 | 75.96 ± 6.58 | 0.095 ± 0.016 |  |  |  |  |  | Group I (sterile saline) |  |  |  |  |
|  |  |  |  |  |  |  |  |  | Group II (VA 375 mg/ml) | 115.0 ± 4.47 | 30.88 ± 3.81 | 76.64 ± 4.57 | 0.098 ± 0.016 |  |  |  |  |  | Group II (VA 375 mg/ml) |  |  |  |  |
|  |  |  |  |  |  |  |  |  | Group III (INH) | 125.3 ± 7.69 | 39.42 ± 4.19 | 90.02 ± 5.80 | 0.200 ± 0.031 |  |  |  |  |  | Group III (INH) |  |  |  |  |
|  |  |  |  |  |  |  |  |  | Group IV (INH + 250 VA) | 117.5 ± 1.78 | 32.74 ± 2.43 | 80.24 ± 6.56 | 0.154 ± 0.030 |  |  |  |  |  | Group IV (INH + 250 VA) |  |  |  |  |
|  |  |  |  |  |  |  |  |  | Group V (INH + 375 VA) | 114.0 ± 6.49 | 29.34 ± 2.78 | 79.12 ± 4.58 | 0.137 ± 0.020 |  |  |  |  |  | Group V (INH + 375 VA) |  |  |  |  |
|  |  |  |  |  |  |  |  |  | Group VI (INH + SYL) | 114.4 ± 5.15 | 28.22 ± 2.75 | 79.14 ± 4.70 | 0.129 ± 0.012 |  |  |  |  |  | Group VI (INH + SYL) |  |  |  | Tilaye GA, Zerihun MF, Chuffa KA, Arayaselassie M, Seifu D. Vernonia amygdalina del (bitter leaf) extract ameliorates isoniazid (INH) induced liver injury in Swiss albino mice. bioRxiv. 2018 Nov 28:482091. |
| *Vitex negundo L.* | leaves | India | Wistar rats of either sex, | Isoniazid, rifampicin and pyrazinamide | 7.5, 10 and 35 | 6 | 100, 250 and 500 | 35 | Group I(DW) | 50.66 ± 5.64 | 45. 33 ± 3.45 | 113.67 ± 3.80 | 0.50 ± 0.001 |  | 6.33 ± 0.14 |  |  |  | Group I(DW) |  |  |  |  |
|  |  |  |  |  |  |  |  |  | Group II (ATT) | 106.83 ± 7.85 | 129.83 ± 8.45 | 215. 66 ± 20.36 | 1.75 ± 0.05 |  | 6.11 ± 0.34 |  |  |  | Group II (ATT) |  |  |  |  |
|  |  |  |  |  |  |  |  |  | Group III (ATT+ VNE 100) | 115.66 ± 5.04 | 105. 33 ± 9.40 | 119.16 ± 2.92 | 1.60 ± 0.03 |  | 6.7 ± 0.22 |  |  |  | Group III (ATT+ VNE 100) |  |  |  |  |
|  |  |  |  |  |  |  |  |  | Group IV (ATT + 250) | 59.16 ± 7.22 | 55.50 ± 5.97 | 176.33 ± 6.31 | 0.68 ± 0.01 |  | 6.71 ± 0.17 |  |  |  | Group IV (ATT + 250) |  |  |  |  |
|  |  |  |  |  |  |  |  |  | Group V (ATT + 500) | 53.00 ± 3.39 | 47.50 ± 1.78 | 130.66 ± 6.16 | 0.66 ± 0.02 |  | 6.85 ± 0.11 |  |  |  | Group V (ATT + 500) |  |  |  |  |
|  |  |  |  |  |  |  |  |  | Group VI (LIV 52 + ATT) | 48.50 ± 5.96 | 43.33 ± 2.76 | 105.83 ± 9.93 | 0.64 ± 0.02 |  | 7.55 ± 0.73 |  |  |  | Group VI (LIV 52 + ATT) |  |  |  | Tandon VR, Khajuria V, Kapoor B, Kour D, Gupta S. Hepatoprotective activity of Vitex negundo leaf extract against anti-tubercular drugs induced hepatotoxicity. Fitoterapia. 2008 Dec 1;79(7-8):533-8. |
| *Ziziphus oenoplia Mill.* | roots | India | Male Wistar albino rats | Isoniazid and rifampicin | 50 | 6 | 150 and 300 | 21 | Group I (NC) | 75.56 ± 3.12 | 45.02 ± 5.25 | 139.2 ± 2.1 | 0.53 ± 0.03 |  |  |  |  |  | Group I (NC) | 271.2 ± 12.9 | 20.72 ± 3.12 | 22.39 ± 1.30 |  |
|  |  |  |  |  |  |  |  |  | Group II (HC) | 131.34 ± 6.39 | 145.26 ± 6.12 | 275.6 ± 1.82 | 1.22 ± 0.81 |  |  |  |  |  | Group II (HC) | 200.5 ± 11.2 | 12.72 ± 1.86 | 16.04 ± 3.62 |  |
|  |  |  |  |  |  |  |  |  | Group III (HC+ E1) | 104.12 ± 4.42 | 102.11 ± 2.44 | 179.2 ± 2.90 | 1.10 ± 0.12 |  |  |  |  |  | Group III (HC+ E1) | 197.5 ± 12.9 | 14.02 ± 1.53 | 19.84 ± 1.11 |  |
|  |  |  |  |  |  |  |  |  | Group IV (HC + E2) | 85.12 ± 2.56 | 69.27 ± 1.43 | 116.1 ± 3.80 | 0.89 ± 1.20 |  |  |  |  |  | Group IV (HC + E2) | 207.8 ± 10.3 | 16.98 ± 0.86 | 20.09 ± 2.04 |  |
|  |  |  |  |  |  |  |  |  | Group V (HC + SD) | 82.42 ± 7.31 | 65.41 ± 4.71 | 102.7 ± 3.98 | 0.85 ± 0.59 |  |  |  |  |  | Group V (HC + SD) | 228.2 ± 13.2 | 18.44 ± 1.51 | 21.78 ± 1.27 | Rao CV, Rawat AK, Singh AP, Singh A, Verma N. Hepatoprotective potential of ethanolic extract of Ziziphus oenoplia (L.) Mill roots against antitubercular drugs induced hepatotoxicity in experimental models. Asian Pacific Journal of Tropical Medicine. 2012 Apr 1;5(4):283-8. |
| *Acanthospermum hispidum DC.* | aerial | India | Albino Wistar rats of both the sexes | . Isoniazid, rifampicin, pyrazinamide, and ethambutol | 27,40,66 and53 | 6 | 400 | 28 | Group I (control) | 35.02±0.06 **AST** | 36.87±0.31 **ALT** | 45.20±0.34 **ALP** | 0.3325±0.00 |  | 5.283±0.10**TP** |  |  |  |  |  |  |  |  |
|  |  |  |  |  |  |  |  |  | Group II (toxic) | 171.8±2.027 | 183.6±0.88 | 122.8±0.88 | 1.257±0.01 |  | 3.30±0.10 |  |  |  |  |  |  |  |  |
|  |  |  |  |  |  |  |  |  | Group III (STD) | 83.92±0.23 | 82.62±0.79 | 93.90±0.77 | 0.438±0.00 |  | 4.252±0.016 |  |  |  |  |  |  |  |  |
|  |  |  |  |  |  |  |  |  | Group IV (AS extract) | 124.2±1.35 | 139.3±0.90 | 134.1±0.54 | 0.640±0.008 |  | 3.385±0.013 |  |  |  |  |  |  |  |  |
|  |  |  |  |  |  |  |  |  | Group V (AS extract) | 112.6±2.82 | 72.08±0.80 | 156.1±1.42 | 0.399±0.003 |  | 4.642±0.015 |  |  |  |  |  |  |  | Himaja N. Comparative study of hepatoprotective activity of Acanthospermumhispidum plant extract and herbal niosomal suspension against anti-tubercular drug induced hepatotoxicity in rats. Asian J Pharm Clin Res. 2015;8(5):256-9. |
| *Amaranthus graecizans L.* | whole plant | Pakistan | Wistar rats of either sex | isoniazid and rifampicin | 50 and 100 | 5 | 400 | 21 | Group I (control) | 20.6 ± 1.2 | 23.8 ± 2.1 | 50.9 ± 0.6 | 2.1 ± 0.5 |  |  |  |  |  |  |  |  |  |  |
|  |  |  |  |  |  |  |  |  | Group II (toxic) | 45.2 ± 4.8 | 49.1 ± 4.4 | 124.6 ± 19.5 | 4.4 ± 0.1 |  |  |  |  |  |  |  |  |  |  |
|  |  |  |  |  |  |  |  |  | Group III (STD +INH+ RIF) | 40.6 ± 3.5 | 36.3 ±1.7 | 56.7 ± 0.8 | 1.3 ± 0.2 |  |  |  |  |  |  |  |  |  |  |
|  |  |  |  |  |  |  |  |  | Group IV (AG extracts+ INH+RIF) | 26.4 ± 1.7 | 24.7 ± 2.9 | 48.1 ± 0.9 | 1.9 ± 0.1 |  |  |  |  |  |  |  |  |  | Ishtiaq S, Afridi MS, Masood N. Amelioration of isoniazid and rifampicin-induced liver toxicity by Amaranthus graecizans subsp. silvestris in rat. \|\|\| Bangladesh Journal of Pharmacology\|\|\|. 2017 Sep 30;12(3):354-8. |
| *Asparagus racemosus Wild.* | root | India | Male albino rats of Wistar | Isoniazid | 100 | 6 | 100 | 21 | Group I (control) | 27.1 + 1.8 | 62.3 + 4.2 | 115.3 + 7.8 |  |  | 6.70 + 0.45 |  |  |  |  | 6.92 + 0.47 | 45.7 + 3.1 | 4.82 + 0.32 |  |
|  |  |  |  |  |  |  |  |  | Group II (toxic) | 109.5 + 6.5 | 479.6 + 28.3 | 527.8 + 31.2 |  |  | 5.25 + 0.30 |  |  |  |  | 5.15 + 0.30 | 37.5 + 2.2 | 3.85 + 0.22 |  |
|  |  |  |  |  |  |  |  |  | Group III (AR+INH) | 31.7 + 2.3 | 90.4 + 6.6 | 165.7 + 12.1 |  |  | 6.0 + 0.44 |  |  |  |  | 6.25 + 0.46 | 41.2 + 3.0 | 4.38 + 0.32 |  |
|  |  |  |  |  |  |  |  |  | Group IV (Con +INH) | 27.2 + 1.7 | 62.12 + 3.9 | 115.0 + 7.3 |  |  | 6.53 + 0.41 |  |  |  |  | 6.92 + 0.44 | 46.1 + 3.0 | 4.88 + 0.31 | Palanisamy N, Manian S. Protective effects of Asparagus racemosus on oxidative damage in isoniazid-induced hepatotoxic rats: an in vivo study. Toxicology and industrial health. 2012 Apr;28(3):238-44. |
| *Asteracantha longifolia L.* | aerial parts | Bangladesh | Adult male rats | isoniazid and rifampicin | 50 | 6 | 500 | 28 | Group I (control) | 44.166± 10.12 | 22.66± 1.63 | 108.5± 1.87 | 0.766 ± 103 |  | 6.70±219 |  |  |  |  |  |  |  |  |
|  |  |  |  |  |  |  |  |  | Group II (toxic) | 106.33± 4.17 | 51.0± 5.05 | 172.5± 4.41 | 1.80± 0.89 |  | 5.361± 41 |  |  |  |  |  |  |  |  |
|  |  |  |  |  |  |  |  |  | Group III (Treatment) | 81.83 ± 10.67 | 36.83± 6.85 | 152.5± 10.51 | 1.38± 1.32 |  | 6.05± 0.94 |  |  |  |  |  |  |  | Lina SM, Ashab I, Ishtiaq Ahmed M, Shahriar M. Hepatoprotective activity of Asteracantha longifolia (Nees.) extract against anti-tuberculosis drugs induced hepatic damage in Sprague-Dawley rats. Pharmacologyonline. 2012 Dec 30;3:13-9. |
| *Citrus sinensis L.* | peel | Indonesia | male Wistar rats | isoniazid and rifampicin | 50 | 5 | 300, 450 and 600 | 14 | Group I (control) | 130.66 ± 28.04 | 42.96 ± 5.40 |  |  |  |  |  |  |  |  |  |  |  |  |
|  |  |  |  |  |  |  |  |  | Group II (toxic) | 334.68 ± 79.16 | 179.12 ± 41.68 |  |  |  |  |  |  |  |  |  |  |  |  |
|  |  |  |  |  |  |  |  |  | Group III (CS+I+R) | 196.2 ± 40.22 | 71.96 ± 21.30 |  |  |  |  |  |  |  |  |  |  |  |  |
|  |  |  |  |  |  |  |  |  | Group IV (CS +I+R) | 164.72 ± 41.20 | 57.84 ± 16.03 |  |  |  |  |  |  |  |  |  |  |  |  |
|  |  |  |  |  |  |  |  |  | Group V (CS+I +R) | 147.16 ± 20.45 | 51.40 ± 9.38 |  |  |  |  |  |  |  |  |  |  |  | Kosasih E, Chiuman L, Lister IN, Fachrial E. Hepatoprotective effect of citrus sinensis peel extract against isoniazid and rifampicin-induced liver injury in wistar rats. Majalah Obat Tradisional. 2019 Dec 31;24(3):197-203. |
| *Crocus sativus L.* | petals | India | Wistar rats | isoniazid and rifampicin | 50 | 6 | 100 and 200 | 14 | Group I (control) | 87.21 ± 5.23 | 62.23 ± 5.32 | 100.43 ± 5.9 |  |  | 9.34 ± 0.2 |  |  |  |  |  |  |  |  |
|  |  |  |  |  |  |  |  |  | Group II (toxic) | 302.43 ± 4.32 | 143.90 ± 3.54 | 278.21 ± 3.65 |  |  | 3.18 ± 0.53 |  |  |  |  |  |  |  |  |
|  |  |  |  |  |  |  |  |  | Group III (STD+I+R) | 95.01 ± 8.93 | 90.98 ± 2.98 | 138.32 ± 2.43 |  |  | 7.02 ± 0.39 |  |  |  |  |  |  |  |  |
|  |  |  |  |  |  |  |  |  | Group IV (CS +I+R) | 289.83 ± 8.93 | 132.76 ± 4.34 | 193.43 ± 3.64 |  |  | 3.98 ± 0.53 |  |  |  |  |  |  |  |  |
|  |  |  |  |  |  |  |  |  | Group V (CS+I +R) | 145.83 ± 6.83 | 99.21 ± 7.03 | 143.29 ± 4.83 |  |  | 5.08 ± 0.98 |  |  |  |  |  |  |  | Wali AF, Pillai JR, Al Dhaheri Y, Rehman MU, Shoaib A, Sarheed O, Jabnoun S, Razmpoor M, Rasool S, Paray BA, Ahmad P. Crocus sativus L. extract containing polyphenols modulates oxidative stress and inflammatory response against anti-tuberculosis drugs-induced liver injury. Plants. 2020 Jan 30;9(2):167. |
| *Cucumis trigonus Roxb.* | fruits | India | Male Wistar albino rats | isoniazid and rifampicin | 50 | 6 | 100, 250 and 500 | 21 | Group I (control) | 27.27±0.94 | 36.59±0.93 | 191.26±10.11 | 0.63±0.04 |  | 7.94±0.51 |  |  | 1.89±0.024 |  | 31.59±0.93 | 3.931±0.018 | 0.193±0.046 |  |
|  |  |  |  |  |  |  |  |  | Group II (toxic) | 144.51±4.56 | 163.59±2.8 | 263.16±11.36 | 3.84±0.84 |  | 6.24±0.36 |  |  | 6.93±0.013 |  | 11.46±0.73 | 1.583±0.024 | 0.073±0.011 |  |
|  |  |  |  |  |  |  |  |  | Group III (CT+I+R) | 83.14±1.36 | 69.36±4.54 | 193.56±8.24 | 2.04±0.92 |  | 7.68±0.14 |  |  | 4.77±0.026 |  | 16.91±0.34 | 1.893±0.014 | 0.101±0.061 |  |
|  |  |  |  |  |  |  |  |  | Group IV (CT +I+R) | 41.33±2.64 | 28.24±5.64 | 163.22±3.84 | 1.02±0.76 |  | 8.14±0.73 |  |  | 2.14±0.019 |  | 19.14±0.53 | 2.671±0.016 | 0.129±0.054 |  |
|  |  |  |  |  |  |  |  |  | Group V (CT+I +R) | 29.11±2.66 | 20.26±1.87 | 152.54±8.14 | 0.59±0.021 |  | 8.33±0.51 |  |  | 1.73±0.011 |  | 24.59±0.49 | 3.73 ±0.01 | 0.151±0.034 |  |
|  |  |  |  |  |  |  |  |  | Group VI (STD +I+R) | 32.63±1.69 | 29.59±1.94 | 182.69±3.91 | 0.96±0.02 |  | 7.89±0.53 |  |  | 1.13±0.054 |  | 30.11±0.73 | 3.91±0.014 | 0.203±0.0123 | Gopalakrishnan SB, Kalaiarasi T. Hepatoprotective activity studies of Cucumis trigonus ROXB against rifampicin-isoniazid-induced toxicity in rats. Eur J Pharm Med Res. 2015;2(6):141-6. |
| *Embeliatsjeriam-cottam A.DC.* | Fruit | India | adult male Wistar rats | isoniazid | 50 | 6 | 200 | 30 | Group I (control) | 45.67 ± 2.89 | 35.76 ± 1.40 |  | 0.77 ± 0.03 |  | 7.60 ± 0.11 |  |  |  |  | 101.4± 1.31 | 280.9± 1.50 | 45.36 ±1.22 |  |
|  |  |  |  |  |  |  |  |  | Group II (toxic) | 83.33 ±1.49 | 188.3 ± 1.81 |  | 1.90 ± 0.02 |  | 5.22 ± 0.16 |  |  |  |  | 50.35 ±1.26 | 92.75 ±1.4 | 19.16±0.28 |  |
|  |  |  |  |  |  |  |  |  | Group III (ETC) | 56.00 ± 1.15 | 85.35 ± 1.23 |  | 0.86 ± 0.01 |  | 7.21 ± 0.05 |  |  |  |  | 81.56± 0.60 | 246.4 ±0.55 | 36.30 ±0.56 |  |
|  |  |  |  |  |  |  |  |  | Group IV (ETC) | 73.50 ± 1.62 | 106.1 ±1.21 |  | 0.53 ± 0.18 |  | 6.44± 0.08 |  |  |  |  | 98.44± 0.44 | 259.5 ±0.89 | 30.64 ±0.48 |  |
|  |  |  |  |  |  |  |  |  | Group V (STD LIV52) | 56.50 ± 1.25 | 80.21 ± 1.13 |  | 0.92 ± 0.02 |  | 6.76 ± 0.12 |  |  |  |  | 84.79± 0.60 | 254.5 ±1.86 | 32.92± 0.62 | Sambrekar SN, Patil PA, Kangralkar VA. Protective effect of EmbeliaTsjeriam–Cottam fruit extracts on isoniazid induced hepatotoxicity in wistar rats. Int J Pharm Sci Res. 2010;4(1):136-9. |
| *Euphorbia fusiformis Buch-Ham.l* | tuber | India | Male albino Wistar rats and Swiss albino mice | Rifampicin | 1000 | 6 | 125, 250 and 500 | 15 | Group I (control) | 99.67 ± 6.90 | 99.83 ± 6.82 | 268.50 ± 7.40 | 0.50 ± 0.03 |  | 6.5 ± 0.53 |  |  |  |  |  |  |  |  |
|  |  |  |  |  |  |  |  |  | Group II (toxic) | 247.50 ± 7.41 | 158.67 ± 6.55 | 433.17 ± 8.91 | 2.05 ± 0.29 |  | 4.7 ± 0.42 |  |  |  |  |  |  |  |  |
|  |  |  |  |  |  |  |  |  | Group III (STD) | 139.17 ± 7.09 | 108.33 ± 5.30 | 272.33 ± 0.03 | 0.7 ± 0.03 |  | 5.7 ± 0.52 |  |  |  |  |  |  |  |  |
|  |  |  |  |  |  |  |  |  | Group IV (GC) | 140.67 ± 6.83 | 110.83 ± 4.52 | 282.67 ± 0.02 | 0.08 ± 0.02 |  | 5.4 ± 0.51 |  |  |  |  |  |  |  | Anusuya N, Raju K, Manian S. Hepatoprotective and toxicological assessment of an ethnomedicinal plant Euphorbia fusiformis Buch.-Ham. ex D. Don. Journal of ethnopharmacology. 2010 Feb 3;127(2):463-7. |
| *Ficus benghalensisL.* | root | India | Male albino Wistar rats and Swiss albino mice | isoniazid and rifampicin | 100 | 6 | 100, 200 and 300 | 21 | Group I (control) | 187±11 | 69±0.6 | 261±32 | 0.43±0.02 |  | 13±0.77 |  |  |  |  | 91.13±0.14 |  |  |  |
|  |  |  |  |  |  |  |  |  | Group II (toxic) | 239±18 | 122±27 | 308±38 | 0.57±0.14 |  | 11±0.23 |  |  |  |  | 43.6±0.27 |  |  |  |
|  |  |  |  |  |  |  |  |  | Group III (FB) | 149±1.1 | 54±1.8 | 250±26 | 0.37±0.04 |  | 12±0.23 |  |  |  |  | 94.52±0.36 |  |  |  |
|  |  |  |  |  |  |  |  |  | Group IV (FB) | 175±3.6 | 94±15 | 256±24 | 0.53±0.08 |  | 12±0.26 |  |  |  |  | 87.27±0.12 |  |  |  |
|  |  |  |  |  |  |  |  |  | Group V (FB) | 158±3.7 | 66±1.1 | 266±16 | 0.33±0.02 |  | 12±0.06 |  |  |  |  | 98.15±0.6 |  |  |  |
|  |  |  |  |  |  |  |  |  | Group VI (STD Liv52) | 170±2.6 | 67±1.7 | 212±18 | 0.73±0.11 |  | 13±0.31 |  |  |  |  | 89.6±0.25 |  |  | Parameswari SA, Saleem TS, Chandrasekar KB, Chetty CM. Protective role of Ficus benghalensis against isoniazid-rifampicin induced oxidative liver injury in rat. Revista Brasileira de Farmacognosia. 2012;22:604-10. |
| *Ficus religiosa L.* | Leaves | India | Male albino Wistar rats and Swiss albino mice | isoniazid and rifampicin | 100 | 6 | 100, 200 and 300 | 21 | Group I (control) | 289±0.3 | 92±0.2 | 347±0.01 | 1.8±0.2 |  | 5.7±0.2 |  |  |  |  | 32.4±0.63 |  |  |  |
|  |  |  |  |  |  |  |  |  | Group II (toxic) | 584±0.5 | 212±0.6 | 421±0.36 | 2.4±0.3 |  | 6.3±0.6 |  |  |  |  | 18.3± .04 |  |  |  |
|  |  |  |  |  |  |  |  |  | Group III (STD liv52) | 351±0.9 | 150±0.6 | 356±0.07 | 1.9±0.5 |  | 5.8±0.5 |  |  |  |  | 19.3±0.13 |  |  |  |
|  |  |  |  |  |  |  |  |  | Group IV (FR) | 272±0.2 | 138±0.4 | 335±0.07 | 2±0.1 |  | 5.9±0.3 |  |  |  |  | 15.5±0.24 |  |  |  |
|  |  |  |  |  |  |  |  |  | Group V (FR) | 309±0.5 | 102±0.2 | 347±0.03 | 1.5±0.4 |  | 5.7±0.4 |  |  |  |  | 28.2±0.3 |  |  |  |
|  |  |  |  |  |  |  |  |  | Group VI (FR) | 365±0.1 | 143±0.8 | 556±0.9 | 1.6±0.1 |  | 5.7±0.1 |  |  |  |  | 21.2±1.12 |  |  | Parameswari SA, Chetty CM, Chandrasekhar KB. Hepatoprotective activity of Ficus religiosa leaves against isoniazid+ rifampicin and paracetamol induced hepatotoxicity. Pharmacognosy research. 2013 Oct;5(4):271. |
| *Glinus lotoides L.* | whole plant | India | Wister strain albino rats | Rifampicin | 100 | 6 | 200 and 400 | 40 hrs. | Group I (control) | 31 ± 1.25 | 33 ± 1.25 | 23 ± 1.25 | 0.6 ± 0.17 |  |  |  |  |  |  |  |  |  |  |
|  |  |  |  |  |  |  |  |  | Group II (toxic) | 103 ± 1.25 | 155 ± 1.25 | 65 ± 1.25 | 1.8 ± 0.0285 |  |  |  |  |  |  |  |  |  |  |
|  |  |  |  |  |  |  |  |  | Group III (STD) | 45 ± 1.25 | 32.5 ± 1.7 | 27 ± 1.25 | 0.7 ± 0.12 |  |  |  |  |  |  |  |  |  |  |
|  |  |  |  |  |  |  |  |  | Group IV (EEGL) | 54 ± 1.8 | 47 ±1.25 | 43.5 ± 1.7 | 1.6 ± 0.03 |  |  |  |  |  |  |  |  |  |  |
|  |  |  |  |  |  |  |  |  | Group V (EEGL) | 49 ± 1.1 | 36.5 ± 1.7 | 31 ± 1.25 | 1.1 ± 0.12 |  |  |  |  |  |  |  |  |  | Rameshwari KS. Anti-Hepatotoxic Effect of *Glinus lotoides* Linn. on Rifampicin Induced Liver Impairment in Albino Rats. Journal of Natural Remedies. 2023 Jun 13:423-7. |
| *Curcuma longa L. (Haridra ghrit)* | rhizomes | India | Wistar male and female rats | Isoniazid, rifampicin and pyrazinamide | 0.8ml | 6 | 100,300 and 700 | 21 | Group I (control) | 120.45±2.75 | 48.90±3.39 | 88.96±3.49 | 0.50±0.04 |  |  |  |  |  |  |  |  |  |  |
|  |  |  |  |  |  |  |  |  | Group II (toxic) | 282.07±3.89 | 90.99±2.99 | 382.53±5.50 | 2.53±0.41 |  |  |  |  |  |  |  |  |  |  |
|  |  |  |  |  |  |  |  |  | Group III (STD) | 153.83±5.62 | 58.53±2.72 | 148.76±3.75 | 0.79±0.05 |  |  |  |  |  |  |  |  |  |  |
|  |  |  |  |  |  |  |  |  | Group IV (HG) | 163.34±4.06 | 60.30±3.99 | 155.09±4.76 | 0.83±0.05 |  |  |  |  |  |  |  |  |  | Vyavahare T, Narwekar M, Nandedkar S, Patil S. EFFECT OF HARIDRA GHRIT IN HEPATOTOXICITY INDUCED BY ANTITUBERCULOSIS DRUGS-AN ANIMAL STUDY. |
| *Leucas cephalotes Roth.* | whole Plant | India | Sprague Dawley rats | Isoniazid and rifampicin | 100 | 6 | 200 and 400 | 21 | Group I (control) | 58.21±4.66 | 45.23±2.84 | 102.00±5.02 | 0.280±0.031 |  |  |  |  | 58.37±4.21 |  | 1.63±0.09 |  | 38.67±2.63 |  |
|  |  |  |  |  |  |  |  |  | Group II (toxic) | 136.44 ±9.24 | 86.63±6.42 | 202.57±8.55 | 0.860±0.052 |  |  |  |  | 86.78±5.64 |  | 0.42±0.02 |  | 19.21±1.24 |  |
|  |  |  |  |  |  |  |  |  | Group III (LC) | 76.6 ±5.83 | 62.66±4.67 | 132.21±8.75 | 0.450±0.021 |  |  |  |  | 73.63±5.23 |  | 1.32±0.08 |  | 29.67±2.05 |  |
|  |  |  |  |  |  |  |  |  | Group IV (LC) | 69.32±4.76 | 54.82±3.56 | 121.05±10.67 | 0.410±0.035 |  |  |  |  | 68.44±4.67 |  | 1.53±0.1 |  | 31.24±1.56 |  |
|  |  |  |  |  |  |  |  |  | Group V (STD) | 61.35±5.21 | 49.67±3.98 | 118.63±7.83b | 0.350±0.022 |  |  |  |  | 63.45±3.98 |  | 1.58 ±0.09 |  | 35.06±.04 | Bais B, Saiju P. Ameliorative effect of Leucas cephalotes extract on isoniazid and rifampicin induced hepatotoxicity. Asian Pacific Journal of Tropical Biomedicine. 2014 Jul 1;4:S633-8. |
| *Luffa acutangular L.* | Fruits | India | Wistar rats of either sex | Rifampicin | 100 | 6 | 100, 200 and 400 | 15 | Group I (control) | 33.2± 5.04 | 15.8± 8.13 | 3.3±073 |  |  | 7.88±0.37 |  |  |  |  | 8.60±0.62 | 38.83±3.44 |  |  |
|  |  |  |  |  |  |  |  |  | Group II (toxic) | 114±12.53 | 108±11.19 | 30.82±2.06 |  |  | 4.93±0.13 |  |  |  |  | 2.80±0.84 | 15.9±2.26 |  |  |
|  |  |  |  |  |  |  |  |  | Group III (STD) | 36.4±8.04 | 22.2±9.52 | 8.56±1.23 |  |  | 6.88±0.37 |  |  |  |  | 8.0±0.60 | 37.04±3.33 |  |  |
|  |  |  |  |  |  |  |  |  | Group IV (HAELA) | 68.2±5.87 | 70±8.86 | 20.62±0.94 |  |  | 4.59±0.20 |  |  |  |  | 5.10±0.78 | 33.11±1.31 |  |  |
|  |  |  |  |  |  |  |  |  | Group V (HAELA) | 47±8.69 | 69±5.51 | 18.08±1.98 |  |  | 6.88±0.23 |  |  |  |  | 6.87±0.68 | 38.32±4.67 |  |  |
|  |  |  |  |  |  |  |  |  | Group VI (HAELA) | 21±5.14 | 17.8± 3.11 | 6.02±0.73 |  |  | 7.08±0.21 |  |  |  |  | 8.68±0.36 | 41.95±2.86 |  | Jadhav VB, Thakare VN, Suralkar AA, Deshpande AD, Naik SR. Hepatoprotective activity of Luffa acutangula against CCl 4 and rifampicin induced liver toxicity in rats: a biochemical and histopathological evaluation. |
| *Mirabilis jalapa L.* | leaves | India | Adult albino rats | Isoniazid, rifampicin, pyrazinamide and ethambutol | 27, 40, 66 and 53 | 6 | 250 and 500 | 35 | Group I (control) | 122.67± 6.38 | 63 ±3.02 | 182 ±6.27 | 0.06 ±0.01 |  |  |  |  |  |  | 2.47 ±0.12 | 34.99 ±0.98 | 3.55±0.26 |  |
|  |  |  |  |  |  |  |  |  | Group II (toxic) | 386.50 ± 14.60 | 639.33 ±17.96 | 315.83 ±7.46 | 0.39 ±0.01 |  |  |  |  |  |  | 0.74 ±0.06 | 15.49 ±0.57 | 1.62 ±0.66 |  |
|  |  |  |  |  |  |  |  |  | Group III (STD) | 166.67 ±2.90 | 136.33 ±5.76 | 138 ±9.65 | 0.08 ±0.02 |  |  |  |  |  |  | 2.27 ±0.09 | 33.89 ±0.08 | 3.71 ±0.19 |  |
|  |  |  |  |  |  |  |  |  | Group IV (EMJ) | 306.67 ±9.89 | 282 ±13.4 | 208.66 ±6.93 | 0.15 ±0.01 |  |  |  |  |  |  | 1.14±0.04 | 23.44 ±0.89 | 2.08 ±0.78 |  |
|  |  |  |  |  |  |  |  |  | Group V (EMJ) | 201.67 ±12.90 | 192.33 ±16.64 | 160.66 ±4.84 | 0.08 ±0.04 |  |  |  |  |  |  | 1.77 ±0.06 | 29.33 ±0.53 | 2.53 ±0.38 | Jyothi BA, MohanalakshmiS, Anitha K. Protective effect of Mirabilis jalapa leaves on anti-tubercular drugs induced hepatotoxicity. Asian J Pharm Clin Res. 2013;6(3):221-4. |
| *Monotheca buxifolia A.DC.* | Leaves | Pakistan | Male Sprague Dawley rats | Isoniazid and rifampicin | 50 | 6 | 150 and 300 | 21 | Group I (control) | 84.1 ± 7.7 | 48.3 ± 4.0 | 119 ± 5.0 | 0.4 ± 0.1 |  | 7.8 ± 0.1 |  |  |  |  |  |  |  |  |
|  |  |  |  |  |  |  |  |  | Group II (toxic) | 131 ± 6.1 | 140 ± 6.2 | 184 ± 7.6 | 1.2 ± 0.1 |  | 3.5 ± 0.2 |  |  |  |  |  |  |  |  |
|  |  |  |  |  |  |  |  |  | Group III (MBE) | 115 ± 7.2 | 114 ± 8.0 | 120 ± 8.3 | 0.9 ± 0.1 |  | 5.7 ± 0.5 |  |  |  |  |  |  |  |  |
|  |  |  |  |  |  |  |  |  | Group IV (MBE) | 91.3 ± 7.1 | 62.5 ± 5.5 | 114 ± 7.0 | 0.7 ± 0.2 |  | 6.7 ± 0.3 |  |  |  |  |  |  |  |  |
|  |  |  |  |  |  |  |  |  | Group V (STD) | 85.5 ± 6.3 | 54.3 ± 4.6 | 118 ± 9.4 | 0.4 ± 0.1 |  | 7.4 ± 0.3b |  |  |  |  |  |  |  | Ullah I, Khan JA, Adhikari A, Shahid M. Hepatoprotective effect of Monothecabuxifolia fruit against antitubercular drugs-induced hepatotoxicity in rats. \|\|\| Bangladesh Journal of Pharmacology\|\|\|. 2016 Jan 18;11(1):248-56. |
| *Moringa oleifera L.* | Leaves | India | Male Wistar rats | Isoniazid, rifampicin, pyrazinamide | 7.5, 10 and 35 | 6 | 150, 200 and 250 | 45 | Group I (control) | 62.79 ± 3.79 | 23.68 ± 2.08 | 9.05 ± 0.527 | 0.26 ± 0.02 |  |  |  |  |  |  |  |  |  |  |
|  |  |  |  |  |  |  |  |  | Group II (toxic) | 105.22 ± 4.10 | 45.83 ± 2.12 | 23.11 ± 1.20 | 0.99 ±0.07 |  |  |  |  |  |  |  |  |  |  |
|  |  |  |  |  |  |  |  |  | Group III (MOtE) | 92.91 ±3.50 | 40.53 ± 2.34 | 19.81 ± 1.41 | 0.82 ±0.01 |  |  |  |  |  |  |  |  |  |  |
|  |  |  |  |  |  |  |  |  | Group IV (MOtE) | 85.06 ± 3.03 | 36.39 ± 1.33 | 16.33 ±1.28 | 0.67 ± 0.03 |  |  |  |  |  |  |  |  |  |  |
|  |  |  |  |  |  |  |  |  | Group V (MOtE) | 78.37 ±3.37 | 31.56 ± 2.06 | 12.43 ±0.83 | 0.48 ±0.03 |  |  |  |  |  |  |  |  |  |  |
|  |  |  |  |  |  |  |  |  | Group VI (STD) | 87.11±2.91 | 37.21 ± 1.65 | 17.70 ± 1.12 | 0.69 ±0.03 |  |  |  |  |  |  |  |  |  | Pari L, Kumar NA. Hepatoprotective activity of Moringa oleifera on antitubercular drug-induced liver damage in rats. Journal of Medicinal Food. 2002 Sep 1;5(3):171-7. |
| *Mucuna pruriens L.* | Leaves |  | Adult Wistar albino mice and rats of either sex | Isoniazid and rifampicin | 100 | 6 | 100,200 and 400 | 21 | Group I (control) | 70.88 ± 6.04 | 16.00 ± 0.58 | 38.43 ± 6.29 | 0.70 ± 0.06 |  |  |  |  |  |  | 1.483 ± 0.080 |  | 3.642 ± 0.194 |  |
|  |  |  |  |  |  |  |  |  | Group II (toxic) | 109.00 ± 6.42 | 27.73 ± 2.14 | 148.30 ± 13.2 | 0.70 ± 0.04 |  |  |  |  |  |  | 0.435 ± 0.05 |  | 1.072 ± 0.138 |  |
|  |  |  |  |  |  |  |  |  | Group III (MPE) | 71.67 ± 4.47 | 20.18 ± 0.99 | 90.47 ± 12.42 | 0.58 ± 0.05 |  |  |  |  |  |  | 0.763 ± 0.050 |  | 1.867 ± 0.123 |  |
|  |  |  |  |  |  |  |  |  | Group IV (MPE) | 68.82 ± 3.88 | 18.82 ± 0.89 | 101.00 ± 16.46 | 1.80 ± 0.46 |  |  |  |  |  |  | 0.898 ± 0.103 |  | 2.203 ± 0.253 |  |
|  |  |  |  |  |  |  |  |  | Group V (MPE) | 73.00 ± 5.36 | 20.38 ± 1.79 | 57.10 ± 5.23 | 0.63 ± 0.04 |  |  |  |  |  |  | 0.742 ± 0.096 |  | 1.822 ± 0.236 |  |
|  |  |  |  |  |  |  |  |  | Group VI (STD) | 66.10 ± 7.04 | 13.55 ± 2.12 | 57.73 ± 10.61 | 1.75 ± 0.51 |  |  |  |  |  |  | 0.793 ± 0.063 |  | 1.945 ± 0.155 |  |
|  |  |  |  |  |  |  |  |  | Group VII (MPE Alone) | 61.10 ± 5.58 | 17.25 ± 1.68 | 64.78 ± 8.27 | 0.95 ± 0.04 |  |  |  |  |  |  | 1.162 ± 0.193 |  | 2.848 ± 0.474 | Obogwu MB, Akindele AJ, Adeyemi OO. Hepatoprotective and in vivo antioxidant activities of the hydroethanolic leaf extract of Mucuna pruriens (Fabaceae) in antitubercular drugs and alcohol models. Chinese journal of natural medicines. 2014 Apr 1;12(4):273-83. |
| *Ocimum sanctum L.* | Leaves | India | adult albino rats of either sex of Wistar strain | Isoniazid, rifampicin, pyrazinamide | 27,54 and 135 | 6 | 200 | 50 | Group I (control) | 31.0±2.30 | 35.16±0.80 | 11.0±0.89 | 2.1±0.32 |  | 7.6±0.50 |  |  |  |  |  |  |  |  |
|  |  |  |  |  |  |  |  |  | Group II (Control 2) | 32.1±3.20 | 34.26±0.50 | 11.5±0.66 | 2.0±0.22 |  | 7.7±0.40 |  |  |  |  |  |  |  |  |
|  |  |  |  |  |  |  |  |  | Group III (Toxic) | 75±2.91 | 144±2.02 | 42.6±0.99 | 4.05±0.50 |  | 5.0±0.036 |  |  |  |  |  |  |  |  |
|  |  |  |  |  |  |  |  |  | Group IV (OSE) | 33±1.53 | 30±0.58 | 11.5±0.77 | 2.5±0.5 |  | 7.5±0.56 |  |  |  |  |  |  |  |  |
|  |  |  |  |  |  |  |  |  | Group V (Toxic + No Treatment) | 69.5±0.88 | 80.66±2.11 | 37.6±1.21 | 3.2±0.52 |  | 5.3±0.5 |  |  |  |  |  |  |  |  |
|  |  |  |  |  |  |  |  |  | Group VI (OSE) | 28±2.79 | 29.8±2.15 | 11±1.99 | 1±1.24 |  | 6.66±0.17 |  |  |  |  |  |  |  | Ocimum PL. Effect of Ocimum sanctum (OS) leaf extract on hepatotoxicity induced by antitubercular drugs in rats. Indian J PhysiolPharmacol. 2003;47(4). |
| *Phyllanthus debilis J.G* | whole plant | India | male Wistar rats | Isoniazid, rifampicin, pyrazinamide | 50, 100 and 350 | 6 | 200 and 400 | 14 | Group I (control) | 157.35±30.10 | 63.65±5.39 | 262.38±15.64 |  |  |  |  |  |  |  |  |  |  |  |
|  |  |  |  |  |  |  |  |  | Group II (Toxic) | 347.08±28.51 | 78.35±5.80 | 347.57±19.53 |  |  |  |  |  |  |  |  |  |  |  |
|  |  |  |  |  |  |  |  |  | Group III (PDE) | 322.28±13.84 | 72.36±8.71 | 331.82±7.80 |  |  |  |  |  |  |  |  |  |  |  |
|  |  |  |  |  |  |  |  |  | Group IV (PDE) | 291.51±18.75 | 64.58±4.69 | 314.24±18.57 |  |  |  |  |  |  |  |  |  |  |  |
|  |  |  |  |  |  |  |  |  | Group V (STD) | 145.32±10.22 | 57.60±3.45 | 256.76±19.74 |  |  |  |  |  |  |  |  |  |  | Anand Koppal AK, Praveen SE, Tara Shanbhag TS, Ankita Kushal AK, Don Mathew DM, Smita Shenoy SS, Navin Patil NP. Evaluation of effect of ethanolic extract of Phyllanthus debilis on antitubercular drugs induced hepatotoxicity in wistar rats. |
| *Pongamia pinnata L.* | Leaves | India | Adult Wistar rats | Isoniazid and rifampicin | 27 and 40 | 6 | 200 and 400 | 21 | Group I (control) | 128.76±7.32 | 65±4.12 | 188±6.78 | 0.12±0.02 |  |  |  |  |  |  | 3.12±0.32 |  | 4.62±0.34 |  |
|  |  |  |  |  |  |  |  |  | Group II (Toxic) | 390.50±18.70 | 645.45±18.12 | 324.45±8.76 | 0.41±0.03 |  |  |  |  |  |  | 0.79±0.02 |  | 2.82±0.31 |  |
|  |  |  |  |  |  |  |  |  | Group III (STD) | 168.78±3.92 | 138.24±6.23 | 142±7.23 | 0.06±0.04 |  |  |  |  |  |  | 3.12±0.02 |  | 4.12±0.23 |  |
|  |  |  |  |  |  |  |  |  | Group IV (APP) | 312.76±9.12 | 294±14.24 | 211.45±7.23 | 0.17±0.03 |  |  |  |  |  |  | 1.23±0.06 |  | 3.12±0.13 |  |
|  |  |  |  |  |  |  |  |  | Group V (APP) | 211.67±13.80 | 196.45±17.23 | 163.23±6.23 | 0.12±0.07 |  |  |  |  |  |  | 1.92±0.12 |  | 3.45±0.46 | Derangula SS, Muthiah NS, Babu BJ, Somashekar HS, Sukumar E, Prabhu K. Antioxidant and Hepatoprotective Activities of Pongamia Pinnata (PP) Linn. Leaves on Anti-Tubercular Medicines (Isoniazid & Rifampin) Induced Hepatotoxicity in Wistar Rats. |
| *Punica granatum L.* | fruits | India | Adult male albino rats of Wistar strain | Isoniazid and rifampicin | 50 | 6 | 400 | 15 | Group I (control) | 23.65 ± 2.30 | 33.93 ± 3.30 | 47.45 ± 4.69 |  |  |  |  |  |  |  | 633.75 ± 63.36 | 4.58 ± 0.46 | 3.61 ± 0.36 |  |
|  |  |  |  |  |  |  |  |  | Group II (Toxic) | 39.64 ±3.86 | 55.45 ± 5.52 | 87.39 ± 8.65 |  |  |  |  |  |  |  | 357.83 ± 35.12 | 3.536 ± 0.35 | 1.66 ± 0.15 |  |
|  |  |  |  |  |  |  |  |  | Group III (PGE) | 24.41 ± 2.41 | 34.12 ± 3.36 | 47.52 ± 4.60 |  |  |  |  |  |  |  | 621.84 ± 62.35 | 4.43 ± 0.43 | 3.56 ± 0.31 |  |
|  |  |  |  |  |  |  |  |  | Group IV (PGE) | 26.45 ± 2.68 | 38.31 ±3.84 | 50.05 ± 5.02 |  |  |  |  |  |  |  | 633.73 ± 62.64 | 4.46 ± 0.44 | 3.58 ± 0.35 | Yogeeta S, Ragavender HR, Devaki T. Antihepatotoxic effect of Punica granatum. acetone extract against isoniazid-and rifampicin-induced hepatotoxicity. Pharmaceutical biology. 2007 Jan 1;45(8):631-7. |
| *Saccharum officinarum L.* | Stem | Pakistan | adult male albino mice | Isoniazid | 100 | 10 | 50ml | 30 | Group I (control) | 56.00±6.06 | 76.20±7.07 | 160.10±11.94 |  |  |  |  |  |  |  |  |  |  |  |
|  |  |  |  |  |  |  |  |  | Group II (Toxic) | 156.40±42.51 | 265.00±57.6 | 220.20±22.43 |  |  |  |  |  |  |  |  |  |  |  |
|  |  |  |  |  |  |  |  |  | Group III (SCE) | 58.00±3.67 | 83.00±6.86 | 142.20±11.59 |  |  |  |  |  |  |  |  |  |  | Khan SW, Tahir M, Lone KP, Munir B, Latif W. Protective effect of Saccharum officinarum L.(sugar cane) juice on isoniazid induced hepatotoxicity in male albino mice. J Ayub Med Coll Abbottabad. 2015 Jun 20;27(2):346-50. |
| *Sesbania grandiflora L.* | whole plant | India | albino Wistar rats | Isoniazid, rifampicin, pyrazinamide | 0.5ml | 6 | 250 and 500 | 28 | Group I (control) | 30 ± 0.16 | 34.2 ± 0.21 |  | 0.205 ± 0.02 |  | 5.5 ± 0.92 |  |  | 5.83 ± 0.19 |  |  |  | 13.1 ± 0.86 |  |
|  |  |  |  |  |  |  |  |  | Group II (Toxic) | 124.5 ± 5.38 | 175.5 ± 3.12 |  | 1.258 ± 0.06 |  | 3.1 ± 0.08 |  |  | 8.16 ± 0.17 |  |  |  | 4.17 ± 0.04 |  |
|  |  |  |  |  |  |  |  |  | Group III (STD) | 51.5 ± 0.62 | 83.8 ± 0.67 |  | 0.356 ± 0.07 |  | 5.15 ± 1.2 |  |  | 6.11 ± 0.09 |  |  |  | 8.78 ± 0.08 |  |
|  |  |  |  |  |  |  |  |  | Group IV (EESG) | 90.6 ± 0.92 | 122.3 ± 4.62 |  | 0.562 ± 1.02 |  | 3.4 ± 0.82 |  |  | 7.44 ± 0.32 |  |  |  | 6.15 ± 1.04 |  |
|  |  |  |  |  |  |  |  |  | Group V (EESG) | 72.5 ± 0.61 | 111.5 ± 5.26 |  | 0.403 ± 0.06 |  | 5.5 ± 0.04 |  |  | 5.06 ± 0.46 |  |  |  | 7.91 ± 0.14 | Kuttiappan A, Chenchula S, Vanangamudi M, Bhatt S, Chikatipalli R, Bhanu PS, Bandaru N. Hepatoprotective effect of flavonoid rich fraction of Sesbania grandiflora: Results of In vivo, in vitro, and molecular docking studies. Journal of Ayurveda and Integrative Medicine. 2024 Sep 1;15(5):101036. |
| *Telfairia occidentalis Hook.* | Fruit |  | male Wistar albino rats | Isoniazid and rifampicin | 100 | 6 | 125, 250 and 500 | 60 | Group I (control) | 99.0 ± 10.0 | 34.3 ± 4.2 | 37.3 ± 2.5 | 14.1 ± 2 |  | 43.3 ± 2.9 |  |  |  |  |  |  |  |  |
|  |  |  |  |  |  |  |  |  | Group II (Toxic) | 124.0 ± 11.1 | 40.0 ± 3.6 | 49.3 ± 4.9 | 17.8 ± 2.0 |  | 30.0 ± 0.8 |  |  |  |  |  |  |  |  |
|  |  |  |  |  |  |  |  |  | Group III (STD) | 79.0 ± 7.4 | 26.0 ± 5.0 | 29.8 ± 4.6 | 8.5 ± 1.0 |  | 46.3 ± 4.8 |  |  |  |  |  |  |  |  |
|  |  |  |  |  |  |  |  |  | Group IV (TOPE) | 76.3 ± 5.5 | 21.7 ± 2.9 | 28.0 ± 2.7 | 8.3 ± 1.5 |  | 48.3 ± 7.6 |  |  |  |  |  |  |  |  |
|  |  |  |  |  |  |  |  |  | Group V (TOPE) | 70.3 ± 9.6 | 19.3 ± 2.1 | 26.3 ± 4.0 | 8.0 ± 0.6 |  | 49.7 ± 0.6 |  |  |  |  |  |  |  |  |
|  |  |  |  |  |  |  |  |  | Group VI (STD) | 75.0 ± 5.6 | 21.0 ± 1.7 | 26.0 ± 3.6 | 8.8 ± 0.5 |  | 49.7 ± 0.6 |  |  |  |  |  |  |  |  |
|  |  |  |  |  |  |  |  |  | Group VII (TOPE alone) | 70.7 ± 4.0 | 22.0 ± 1.0 | 21.0 ± 1.0 | 8.9 ± 0.5 |  | 44.3 ± 8.0 |  |  |  |  |  |  |  | Nwidu LL, Oboma YI. Telfairia occidentalis (Cucurbitaceae) pulp extract mitigates rifampicin-isoniazid-induced hepatotoxicity in an in vivo rat model of oxidative stress. Journal of Integrative Medicine. 2019 Jan 1;17(1):46-56. |
| *Terminala chebula Retz.* | fruit | India | male Wistar rats | Isoniazid, rifampicin, pyrazinamide | 50 250 and 100 | 6 | 5, 100 and 200 | 12 weeks | Group I (control) | 110±4.3 | 155 ± 5.4 | 144±3.9 | 0.27±0.07 |  |  |  |  | 19.4±1.4 |  | 25.3±1.8 |  |  |  |
|  |  |  |  |  |  |  |  |  | Group II (toxic) | 503±6.7 | 554± 7.9 | 410±5.8 | 0.54± 0.09 |  |  |  |  | 59.1±4.2 |  | 7.1±0.8 |  |  | Tasduq SA, Singh K, Satti NK, Gupta DK, Suri KA, Johri RK. Terminalia chebula (fruit) prevents liver toxicity caused by sub-chronic administration of rifampicin, isoniazid and pyrazinamide in combination. Human &experimental toxicology. 2006 Mar;25(3):111-8. |

| **Plant Scientific Name** | **Authors** | **Anti-TB drugs** | **Toxic group (anti-TB drugs) mg/kg** | | **Standard group (Silymarin) mg/kg** | | **Treatment group (PE)**  **mg/kg** | | **% protection by Standard (Silymarin) compared to the Toxic group** | **% protection by Treatment group (PE) compared to the Toxic group**  **(* Mean values are for the highest conc. of PE)** | **% protection by Treatment group (PE) compared to the Standard (Silymarin)** |
| --- | --- | --- | --- | --- | --- | --- | --- | --- | --- | --- | --- |
|  |  |  | **Conc. of drugs mg/kg** | **Mean mg/kg b. w** | **Conc. of Standard mg/kg** | **Mean mg/kg b. w** | **Conc. of PE mg/kg** | **Mean mg/kg b. w** |  |  |  |
| *Abelmoschus ficulneus L.* | Swetha et al.,2021 **^(10)^** | INH and RIF | 50 | 119.19 | 100 | 59.13 | 100,200, 400 | 94.15 | 50.39 | 21.01 | -59.23 |
| *Adenanthera pavonina L.* | Mujahid *et al*.,2013 **^(12)^** | INH and RIF | 50 | 162.83 | 100 | 66.54 | 100 and 200 | 93.95 | 59.14 | 42.30 | -41.19 |
| *Allium cepa L* | Sagar *et al*.,2022**^(13)^** | INH and RIF | 50 | 0.21 | 100 | 0.29 | 100 | 0.24 | -38.10 | -14.29 | 17.24 |
| *Allium sativum L* | NASIM *et al*.,2017 **^(14)^** | INH | 50 | 130.9 | 200 | 36.4 | 250 | 28.1 | 72.19 | 78.53 | 22.80 |
| *Annona squamosa L.* | Uduman *et al.*,2011 **^(9)^** | INH and RIF | 100 | 124.91 | 2.5 | 49.52 | 250 and 500 | 67.02 | 60.36 | 46.35 | -35.34 |
| *Persea americana Mill. and Telfairia occidentalis Hook.f.* | Jibril *et al*.,2015 **^(15)^** | INH, RIF, PZA and EMB | 51.4 | 56.9 | NA | NA | 125 and 200 | 13.2 | 100.00 | 76.80 | NA |
| *Azadirachta indica A.* | Althaiban *et al.*,2019 **^(16)^** | RIF | 50 | 130.36 | 25 |  | 250 and 500 | 75.37 | 100.00 | 42.18 | NA |
| *Bacopa monnieri L.* | Evan *et al*.,2016 **^(17)^** | INH and RIF | 50 | 103 | 25 | 47 | 500 | 45 | 54.37 | 56.31 | 4.26 |
| *Calotropis procera (Aiton)* | Noor *et al*.,2014 **^(18)^** | INH and RIF | 50 | 41.83 | NA | NA | 150 | 13.83 | 100.00 | 66.94 | NA |
| *C. frutescens* | Angeli B *et al*.,2014 **^(19)^** | INH and RIF | 50 | 39.4 | NA | NA | 100 and 200 | 36 | 100.00 | 8.63 | NA |
| *Carica papaya L.* | Aashish *et al*., 2013 **^(20)^** | INH and RIF | 125 | 218.8 | 25 | 108.9 | 400 | 139 | 50.23 | 36.47 | -27.64 |
| *Cassia auriculata L.* | Ashish *et al*.,2014 **^(21)^** | INH, RIF and PZA | 54 | 135.53 | 100 | 75.6 | 300 and 600 | 70.7 | 44.22 | 47.83 | 6.48 |
| *Centella asiatica L.* | Kavisa *et al*.,2016 **^(22)^** | INH | 50 | 26.23 | 50 | 6.97 | 20, 40, 60, and 100 | 6.72 | 73.43 | 74.38 | 3.59 |
| *Cissampelos pareira L.* | Sangameswaran *et al*.,2012 **^(23)^** | INH and RIF | 50 | 82.33 | 100 | 46.8 | 100, 200 and 400 | 73.69 | 43.16 | 10.49 | -57.46 |
| *Cissus quadrangularisL.* | SWAMY *et al*.,2012 **^(24)^** | RIF | 54 | 97.62 | 50 | 64.01 | 500 | 71.02 | 34.43 | 27.25 | -10.95 |
| *Cnidoscolus chayamansa McVaugh.* | Mariana *et al.*,2019 **^(25)^** | INH, RIF and PZA | 50 and 100 | 174 | 2.5 | 145.5 | 50, 50 and 100 | 143.6 | 16.38 | 17.47 | 1.31 |
| *Crassocephalum*  *vitellinum Benth.* | Kenedy *et al*.,2022 **^(26)^** | RIF | 10 | 262.2 | 100 | 60.98 | 200,400 and 800 | 193.3 | 76.74 | 26.28 | -216.99 |
| *Enseteven tricosum Welw.* | Abebe *et al.*,2021 **^(27)^** | INH and RIF | 75 and 150 | 123.67 | 100 | 40.16 | 200 and 400 | 90 | 67.53 | 27.23 | -124.10 |
| *Ficus carica L.* | N. Y. GOND *et al*.,2008 **^(28)^** | RIF | 50 | 88 |  |  | 200 | 66 | 100.00 | 25.00 | NA |
| *Alpinia officinarum H.* | Shimaila *et al*.,2019 **^(29)^** | INH and RIF | 50 | 98.66 | 100 | 57.68 | 200 and 400 | 87.7 | 41.54 | 11.11 | -52.05 |
| *Garcinia kola H.* | Tweneme *et al*.,2014 **^(89)^** | INH and RIF | 7.5 | 17.56 | NA | NA | 40, 60 and 80 | 7.84 | 100.00 | 55.35 | NA |
| *Allium sativum* | Nasiru A. *et al*.,2012**^(14)^** | INH, RIF, PZA and EMB | 20 | 61.5 | NA | NA | 28.5, 42.8 and 57.1 | 29.5 | 100.00 | 52.03 | NA |
| *Hedyotis corymbosa L.* | Shah Marzia *et al.*,2018**^(31)^** | INH and RIF | 50 | 56.67 | 50 | 21.23 | 500 | 33.5 | 62.54 | 40.89 | -57.80 |
| *Hibiscus vitifolius L.* | Anbu Jeba *et al*.,2012**^(32)^** | INH, RIF and PZA | 7.5, 10 and 35 | 326 | 100 | 48.01 | 400 | 300.46 | 85.27 | 7.83 | -525.83 |
| *Ipomoea carnea J.* | Kumar *et al*.,2013 (**^33)^** | INH, RIF and PZA | 7.5, 10 and 35 | 147.21 | 100 | 68.21 | 125,250 and 500 | 142.61 | 53.66 | 3.12 | -109.07 |
| *Ixora pavetta A.* | Jyothi Reddy *et al*.,2013**^(34)^** | INH and RIF | 100 | 309 | 2.5 | 197 | 100 and 400 | 276 | 36.25 | 10.68 | -40.10 |
| *Jasminum grandiflorum L.* | Netranjali *et al*.,2012**^(35)^** | INH | 54 | 79.17 | 50 | 52.33 | 200 | 64.5 | 33.90 | 18.53 | -23.26 |
| *Lawsonia inermis L.* | S. Sylvester *et al*.,2018**^(36)^** | INH and RIF | 50 and 100 | 208.1 | 100 | 83.58 | 50 and 100 | 52.09 | 59.84 | 74.97 | 37.68 |
| *Maytenus royleanus W.* | Shabbir *et al*.,2020**^(37)^** | INH, RIF, PZA and EMB | 13.5, 6.75,36.0 and 24.8 | 211.2 | NA | NA | 200 and 400 | 169.9 | 100.00 | 19.55 | NA |
| *Nigella sativa L.* | Hafeez *et al.*,2013**^(38)^** | PZA | 20 | 36.7 | NA | NA | 500 | 82.8 | 100.00 | -125.61 | NA |
| *Nyctanthes arbor-tristis L.* | Chaudhary *et al*.,2018**^(39)^** | INH, RIF and PZA | 7.5, 10 and 35 | 123.43 | 100 | 83.8 | 250 and 500 | 114.85 | 32.11 | 6.95 | -37.05 |
| *Ocimum canum Sims.* | Radhika J *et al*.,2018**^(40)^** | INH | 54 | 312 | 50 | 75.3 | 200 and 300 | 246.71 | 75.87 | 20.93 | -227.64 |
| *Otostegia integrifolia B.* | Gugsa *et al*.,2023**^(41)^** | INH and RIF | 100 and 150 | 84 | NA | NA | 200 and 400 | 62.83 | 100.00 | 25.20 | NA |
| *Polygonatum cirrhifolium W.* | Roma Gha *et al*.,2023**^(42)^** | INH and RIF | 50 | 70.58 | 50 | 19.32 | 200 and 400 | 44.89 | 72.63 | 36.40 | -132.35 |
| *Solanum nigrum L.* | Abdel-Hameed *et al.*,2019**^(43)^** | INH and RIF | 50 | 245.49 | 50 | 207.53 | 250, 500 and 1000 | 215.73 | 15.46 | 12.12 | -3.95 |
| *Tamarindus indica L.* | Mohd Amir *et al*.,2015**^(44)^** | INH and RIF | 50 and 100 | 133.15 | 50 | 49.07 | 250 and 500 | 127.08 | 63.15 | 4.56 | -158.98 |
| *Telfairia occidentalis Hook.* | Legbosi Nwidu *et al.*,2018**^(45)^** | INH and RIF | 100 | 40 | 50 | 21 | 125, 250 and 500 | 21.7 | 47.50 | 45.75 | -3.33 |
| *Vernonia amygdalina Del* | Addisu Tilaye *et al*.,2018**^(47)^** | INH | 75 | 39.42 | 100 | 28.22 | 250 and 375 | 32.74 | 28.41 | 16.95 | -16.02 |
| *Vitex negundo L.* | Vishal R. *et al.*,2008 **^(48)^** | INH, RIF and PZA | 7.5, 10 and 35 | 129.83 | NA | NA | 100, 200 and 500 | 105.33 | 100.00 | 18.87 | NA |
| *Ziziphus oenoplia L.* | Ch V Rao *et al.*,2012**^(49)^** | INH and RIF | 50 | 145.26 | 100 | 65.41 | 150 and 300 | 102.11 | 54.97 | 29.71 | -56.11 |
| *Acanthospermum hispidum DC.* | HIMAJA N *et al.,*2015 **^(50)^** | INH, RIF, PZA and EMB | 27,40,66 and53 | 183.6 | 100 | 82.62 | 400 | 72.08 | 55.00 | 60.74 | 12.76 |
| *Amaranthus graecizans L.* | Ishtiaq S *et al.,*2017 **^(51)^** | INH and RIF | 50 and 100 | 49.1 | 100 | 36.3 | 400 | 24.7 | 26.07 | 49.69 | 31.96 |
| *Asparagus racemosus W.* | N Palanisamy *et al*.,2012**^(52)^** | INH | 100 | 479.6 | NA | NA | 100 | 90.4 | 100.00 | 81.15 | NA |
| *Asteracantha longifolia Nees.* | Lina SM *et al*.,2012**^(53)^** | INH and RIF | 50 | 51 | NA | NA | 500 | 36.83 | 100.00 | 27.78 | NA |
| *Citrus sinensis L.* | Kosasih E *et al.*,2019**^(54)^** | INH and RIF | 50 | 179.12 | NA | NA | 300, 450 and 600 | 51.4 | 100.00 | 71.30 | NA |
| *Crocus sativus L.* | Wali AF *et al*.,2020**^(55)^** | INH and RIF | 50 | 143.9 | 10 | 90.98 | 100 and 200 | 99.21 | 36.78 | 31.06 | -9.05 |
| *Cucumis trigonus Roxb.* | Gopalakrishnan SB *et al.*,2015 **^(56)^** | INH and RIF | 50 | 163.59 | 2.5 | 29.59 | 100, 250 and 500 | 20.26 | 81.91 | 87.62 | 31.53 |
| *Embelia tsjeriam-cottam A.DC.* | Sambrekar SN *et a*l.,2010 **^(57)^** | INH | 50 | 188.3 | 5 ml | 80.21 | 200 | 85.35 | 57.40 | 54.67 | -6.41 |
| *Euphorbia fusiformis B.* | Anusuya *et al.,*2010**^(58)^** | RIF | 1000 | 158.67 | 100 | 108.33 | 125, 250 and 500 | 110.83 | 31.73 | 30.15 | -2.31 |
| *Ficus benghalensis L.* | Parameswari SA *et al*.,2012**^(59)^** | INH and RIF | 100 | 122 | 10 | 67 | 100, 200 and 300 | 54 | 45.08 | 55.74 | 19.40 |
| *Ficus religiosa L.* | Parameswari SA *et al.,*2013**^(60)^** | INH and RIF | 100 | 212 | 10 | 150 | 100, 200 and 300 | 102 | 29.25 | 51.89 | 32.00 |
| *Glinus lotoides L.* | Rameshwari KS *et al.,*2023**^(61)^** | RIF | 100 | 155 | 100 | 32.5 | 200 and 400 | 36.5 | 79.03 | 76.45 | -12.31 |
| *Curcuma longa* ***L. (****Haridra Ghrit)* | Vyavahare T *et al.,*2024**^(62)^** | INH, RIF and PZA | 100,300 and 700 | 90.99 | 25 | 58.53 | 100,300 and 700 | 60.3 | 35.67 | 33.73 | -3.02 |
| *Leucas cephalotes R.* | Bais B *et al.,* 2014**^(63)^** | INH and RIF | 100 | 86.63 | 200 | 49.67 | 200 and 400 | 54.82 | 42.66 | 36.72 | -10.37 |
| *Luffa acutangula L.* | Jadhav VB *et al.*,2010**^(64)^** | RIF | 100 | 108 | 200 | 22.2 | 100, 200 and 400 | 17.8 | 79.44 | 83.52 | 19.82 |
| *Mirabilis jalapa L.* | Jyothi BA *et al.*,2013**^(65)^** | INH, RIF, PZA and EMB | 27, 40, 66 and 53 | 639.33 | 100 | 136.33 | 250 and 500 | 192.33 | 78.68 | 69.92 | -41.08 |
| *Monotheca buxifolia F.* | Ullah I *et al.,*2016**^(66)^** | INH and RIF | 50 | 140 | 100 | 54.3 | 150 and 300 | 62.5 | 61.21 | 55.36 | -15.10 |
| *Moringa oleifera L.* | Pari L *et al.*,2002**^(67)^** | INH, RIF and PZA | 7.5, 10 and 35 | 45.83 | 200 | 37.21 | 150, 200 and 250 | 31.56 | 18.81 | 31.14 | 15.18 |
| *Mucuna pruriens L.* | Obogwu MB *et al*.,2014**^(68)^** | INH and RIF | 100 | 27.73 | 50 | 13.55 | 100,200 and 400 | 18.82 | 51.14 | 32.13 | -38.89 |
| *Ocimum sanctum L.* | Ocimum P *et al*.,2003**^(69)^** | INH, RIF and PZA | 27,54 and 135 | 144 | NA | NA | 200 | 30 | 100.00 | 79.17 | NA |
| *Phyllanthus debilis K.* | Anand K et al.,2014**^(70)^** | INH, RIF and PZA | 50, 100 and 350 | 78.35 | 50 | 57.6 | 200 and 400 | 64.58 | 26.48 | 17.57 | -12.12 |
| *Pongamia pinnata L.* | Derangula SS *et al*.,2022**^(71)^** | INH and RIF | 27 and 40 | 645.45 | 100 | 138.24 | 200 and 400 | 196.45 | 78.58 | 69.56 | -42.11 |
| *Punica granatum L.* | Yogeeta S *et al*.,2007 **^(72)^** | INH and RIF | 50 | 55.45 | NA | NA | 400 | 34.12 | 100.00 | 38.47 | NA |
| *Saccharum officinarum L.* | Khan SW *et al.,2*015**^(73)^** | INH | 50 | 265 | NA | NA | 50 | 83 | 100.00 | 68.68 | NA |
| *Sesbania grandiflora L.* | Kuttiappan A *et al*.,2024 **^(74)^** | INH, RIF and PZA | 0.5 | 175.5 | 100 | 83.8 | 250 and 500 | 111.5 | 52.25 | 36.47 | -33.05 |

***Supplementary table no.2: Effect of plant extracts on the ALT levels compared with toxic group (anti-TB drugs) and Silymarin (standard)***

| **Plant Scientific Name** | **Authors** | **Anti-TB drugs** | **Toxic group (anti-TB drugs) mg/kg** | | **Standard group (Silymarin) mg/kg** | | **Treatment group (PE)**  **mg/kg** | | **% protection by Standard (Silymarin) compared to the Toxic group** | **% protection by Treatment group (PE) compared to the Toxic group**  **(* Mean values are for the highest conc. of PE)** | **% protection by Treatment group (PE) compared to the Standard (Silymarin)** |
| --- | --- | --- | --- | --- | --- | --- | --- | --- | --- | --- | --- |
|  |  |  | **Conc. of drugs mg/kg** | **Mean mg/kg b. w** | **Conc. of Standard mg/kg** | **Mean mg/kg b. w** | **Conc. of PE mg/kg** | **Mean mg/kg b. w** |  |  |  |
| *Abelmoschus ficulneus L.* | Swetha *et al*.,2021**^(10)^** | INH and RIF | 50 | 259.13 | 100 | 145.24 | 100,200, 400 | 203.51 | 43.95 | 21.46 | -40.12 |
| *Adenanthera pavonina L.* | Mujahid *et al*.,2013**^(12)^** | INH and RIF | 50 | 290.36 | 100 | 156.31 | 100 and 200 | 175.1 | 46.17 | 39.70 | -12.02 |
| *Allium sativum L* | NASIM *et al*.,2017**^(14)^** | INH | 50 | 385.6 | 200 | 143.8 | 250 | 118.4 | 62.71 | 69.29 | 17.66 |
| *Annona squamosa L.* | Uduman *et al.*,2011**^(9)^** | INH and RIF | 100 | 243.64 | 2.5 | 111.29 | 250 and 500 | 150.55 | 54.32 | 38.21 | -35.28 |
| *Persea americana Mill. and Telfairia occidentalis Hook.f.* | Jibril *et al*.,2015**^(15)^** | INH, RIF, PZA and EMB | 51.4 | 96.8 | NA | NA | 125 and 200 | 36.4 | 100.00 | 62.40 | NA |
| *Azadirachta indica A. Juss.* | Althaiban *et al.*,2019**^(16)^** | RIF | 50 | 143.22 | 25 | NA | 250 and 500 | 81.32 | 100.00 | 43.22 | NA |
| *Bacopa monnieri L.* | Evan *et al*.,2016**^(17)^** | INH and RIF | 50 | 198 | 25 | 106 | 500 | 104 | 46.46 | 47.47 | 1.89 |
| *Calotropis procera Aiton* | Noor *et al*.,2014**^(18)^** | INH and RIF | 50 | 154.67 | NA | NA | 150 | 83.33 | 100.00 | 46.12 | NA |
| *Carica papaya L.* | Aashish *et al*., 2013**^(20)^** | INH and RIF | 125 | 251.9 | NA | NA | 400 | 150.5 | 100.00 | 40.25 | NA |
| *Cassia auriculata L.* | Ashish *et al*.,2014**^(21)^** | INH, RIF and PZA | 54 | 314.16 | 100 | 186.01 | 300 and 600 | 253.6 | 40.79 | 19.28 | -36.34 |
| *Centella asiatica L.* | Kavisa *et al*.,2016**^(22)^** | INH | 50 | 51.43 | 50 | 35.79 | 20, 40, 60, and 100 | 30.11 | 30.41 | 41.45 | 15.87 |
| *Cissampelos pareira L.* | Sangameswaran *etal*.,2012**^(23)^** | INH and RIF | 50 | 389.78 | 100 | 239.22 | 100, 200 and 400 | 365.59 | 38.63 | 6.21 | -52.83 |
| *Cissus quadrangularisL.* | SWAMY *et al*.,2012**^(24)^** | RIF | 54 | 267.7 | 50 | 61.7 | 500 | 187.5 | 76.95 | 29.96 | -203.89 |
| *Cnidoscolus chayamansa McVaugh* | Mariana *et al.*,2019 **^(25)^** | INH, RIF and PZA | 50 and 100 | 182.3 | 2.5 | 186 | 50, 50 and 100 | 184.6 | -2.03 | -1.26 | 0.75 |
| *Crassocephalum vitellinum Benth.* | Kenedy *et al*.,2022**^(26)^** | RIF | 10 | 173.8 | 100 | 81.28 | 200,400 and 800 | 145.3 | 53.23 | 16.40 | -78.76 |
| *Enseteven tricosum Welw.* | Abebe *et al.*,2021**^(27)^** | INH and RIF | 75 and 150 | 171 | 100 | 106.33 | 200 and 400 | 120.83 | 37.82 | 29.34 | -13.64 |
| *Alpinia officinarum H.* | Shimaila *et al*.,2019**^(29)^** | INH and RIF | 50 | 216.48 | 100 | 155.82 | 200 and 400 | 202.96 | 28.02 | 6.25 | -30.25 |
| *Garcinia kola H.* | Tweneme *et al*.,2014**^(89)^** | INH and RIF | 7.5 | 116.33 | NA | NA | 40, 60 and 80 | 57.41 | 100.00 | 50.65 | NA |
| *Allium sativum* | Nasiru A. *et al*.,2012**^(30)^** | INH, RIF, PZA and EMB | 20 | 106.5 | NA | NA | 28.5, 42.8 and 57.1 | 43 | 100.00 | 59.62 | NA |
| *Hedyotis corymbosa L.* | Shah Marzia *et al.*,2018**^(31)^** | INH and RIF | 50 | 177.83 | 50 | 151.25 | 500 | 163.17 | 14.95 | 8.24 | -7.88 |
| *Hibiscus vitifolius L.* | Anbu Jeba *et al*.,2012**^(32)^** | INH, RIF and PZA | 7.5, 10 and 35 | 450.65 | 100 | 322.84 | 400 | 411.53 | 28.36 | 8.68 | -27.47 |
| *Ipomoea carnea J.* | Ramesh Kumar *et al*.,2013**^(33)^** | INH, RIF and PZA | 7.5, 10 and 35 | 162.21 | 100 | 72.32 | 125,250 and 500 | 144.21 | 55.42 | 11.10 | -99.41 |
| *Ixora pavetta A.* | Jyothi Reddy *et al*.,2013**^(34)^** | INH and RIF | 100 | 319.2 | 2.5 | 203.1 | 100 and 400 | 239.3 | 36.37 | 25.03 | -17.82 |
| *Lawsonia inermis L.* | S. Sylvester *et al*.,2018**^(36)^** | INH and RIF | 50 and 100 | 376.5 | 100 | 141.4 | 50 and 100 | 140.3 | 62.44 | 62.74 | 0.78 |
| *Maytenus royleanus W.* | Shabbir *et al*.,2020**^(37)^** | INH, RIF, PZA and EMB | 13.5, 6.75,36.0 and 24.8 | 306.47 | NA | NA | 200 and 400 | 252.14 | 100.00 | 17.73 | NA |
| *Nyctanthes arbor-tristis L.* | Chaudhary *et al*.,2018**^(39)^** | INH, RIF and PZA | 7.5, 10 and 35 | 289.6 | 100 | 183.34 | 250 and 500 | 279.7 | 36.69 | 3.42 | -52.56 |
| *Ocimum canum Sims.* | Radhika J *et al*.,2018**^(40)^** | INH | 54 | 997.1 | 50 | 326.61 | 200 and 300 | 876.2 | 67.24 | 12.13 | -168.27 |
| *Polygonatum cirrhifolium W.* | Roma Gha *et al*.,2023**^(42)^** | INH and RIF | 50 | 284.3 | 50 | 130.3 | 200 and 400 | 172.8 | 54.17 | 39.22 | -32.62 |
| *Solanum nigrum L.* | Abdel-Hameed *et al.*,2019**^(43)^** | INH and RIF | 50 | 111.76 | 50 | 56.71 | 250, 500 and 1000 | 70.21 | 49.26 | 37.18 | -23.81 |
| *Tamarindus indica L.* | Mohd Amir *et al*.,2015**^(44)^** | INH and RIF | 50 and 100 | 110.03 | 50 | 33.4 | 250 and 500 | 95.7 | 69.64 | 13.02 | -186.53 |
| *Telfairia occidentalis* | Legbosi Nwidu *et al.*,2018**^(45)^** | INH and RIF | 100 | 49.3 | 50 | 26 | 125, 250 and 500 | 29.8 | 47.26 | 39.55 | -14.62 |
| *Vernonia amygdalina Del* | Addisu Tilaye *et al*.,2018**^(47)^** | INH | 75 | 90.02 | 100 | 79.14 | 250 and 375 | 80.24 | 12.09 | 10.86 | -1.39 |
| *Vitex negundo L.* | Vishal R. *et al.*,2008 (**^48)^** | INH, RIF and PZA | 7.5, 10 and 35 | 215.66 | NA | NA | 100, 200 and 500 | 119.16 | 100.00 | 44.75 | NA |
| *Ziziphus oenoplia L.* | Ch V Rao *et al.*,2012**^(49)^** | INH and RIF | 50 | 275.6 | 100 | 102.7 | 150 and 300 | 179.2 | 62.74 | 34.98 | -74.49 |
| *Acanthospermum hispidum DC.* | HIMAJA N *et al.,*2015 **^(50)^** | INH, RIF, PZA and EMB | 27,40,66 and53 | 122.8 | 100 | 93.9 | 400 | 134.1 | 23.53 | -9.20 | -42.81 |
| *Amaranthus graecizans L.* | Ishtiaq S *et al.,*2017 **^(51)^** | INH and RIF | 50 and 100 | 124.6 | 100 | 56.7 | 400 | 48.1 | 54.49 | 61.40 | 15.17 |
| *Asparagus racemosus W.* | N Palanisamy *et a*l.,2012**^(52)^** | INH | 100 | 527.8 | NA | NA | 100 | 165.7 | 100.00 | 68.61 | NA |
| *Asteracantha longifolia Nees.* | Lina SM *et al.*,2012**^(53)^** | INH and RIF | 50 | 172.5 | NA | NA | 500 | 152.5 | 100.00 | 11.59 | NA |
| *Crocus sativus L.* | Wali AF *et al.*,2020**^(55)^** | INH and RIF | 50 | 278.21 | 10 | 138.32 | 100 and 200 | 143.29 | 50.28 | 48.50 | -3.59 |
| *Cucumis trigonus Roxb.* | Gopalakrishnan SB *et al.*,2015 **^(56)^** | INH and RIF | 50 | 263.16 | 2.5 | 182.69 | 100, 250 and 500 | 152.54 | 30.58 | 42.04 | 16.50 |
| *Euphorbia fusiformis B.* | Anusuya *et al.,*2010**^(58)^** | RIF | 1000 | 433.17 | 100 | 272.33 | 125, 250 and 500 | 282.67 | 37.13 | 34.74 | -3.80 |
| *Ficus benghalensis L.* | Parameswari SA et al.,2012**^(59)^** | INH and RIF | 100 | 308 | 10 | 212 | 100, 200 and 300 | 250 | 31.17 | 18.83 | -17.92 |
| *Ficus religiosa L.* | Parameswari SA *et al.,*2013**^(60)^** | INH and RIF | 100 | 421 | 10 | 356 | 100, 200 and 300 | 335 | 15.44 | 20.43 | 5.90 |
| *Glinus lotoides L.* | Rameshwari KS *et al.,*2023**^(61)^** | RIF | 100 | 65 | 100 | 27 | 200 and 400 | 31 | 58.46 | 52.31 | -14.81 |
| *Haridra Ghrit* | Vyavahare T *et al*.,2024**^(62)^** | INH, RIF and PZA | 100,300 and 700 | 382.53 | 25 | 148.76 | 100,300 and 700 | 155.09 | 61.11 | 59.46 | -4.26 |
| *Leucas cephalotes R.* | Bais B *et al.,* 2014**^(63)^** | INH and RIF | 100 | 202.57 | 200 | 118.63 | 200 and 400 | 121.05 | 41.44 | 40.24 | -2.04 |
| *Luffa acutangula L.* | Jadhav VB *et al.*,2010**^(64)^** | RIF | 100 | 30.82 | 200 | 8.56 | 100, 200 and 400 | 6.02 | 72.23 | 80.47 | 29.67 |
| *Mirabilis jalapa L.* | Jyothi *BA et al.,*2013**^(65)^** | INH, RIF, PZA and EMB | 27, 40, 66 and 53 | 315.83 | 100 | 138 | 250 and 500 | 160.66 | 56.31 | 49.13 | -16.42 |
| *Monotheca buxifolia F.* | Ullah I *et al.,*2016**^(66)^** | INH and RIF | 50 | 184 | 100 | 118 | 150 and 300 | 114 | 35.87 | 38.04 | 3.39 |
| *Moringa oleifera L.* | Pari L *et* al.,2002**^(67)^** | INH, RIF and PZA | 7.5, 10 and 35 | 23.11 | 200 | 17.7 | 150, 200 and 250 | 12.43 | 23.41 | 46.21 | 29.77 |
| *Mucuna pruriens L.* | Obogwu MB *et al*.,2014 (68) | INH and RIF | 100 | 148.3 | 50 | 57.73 | 100,200 and 400 | 57.1 | 61.07 | 61.50 | 1.09 |
| *Ocimum sanctum L.* | Ocimum P *et al.,*2003**^(69)^** | INH, RIF and PZA | 27,54 and 135 | 42.6 | NA | NA | 200 | 11.5 | 100.00 | 73.00 | NA |
| *Phyllanthus debilis K.* | Anand K *et al.*,2014**^(70)^** | INH, RIF and PZA | 50, 100 and 350 | 347.57 | 50 | 256.76 | 200 and 400 | 314.24 | 26.13 | 9.59 | -22.39 |
| *Pongamia pinnata L.* | Derangula SS *et al*.,2022**^(71)^** | INH and RIF | 27 and 40 | 324.4 | 100 | 142 | 200 and 400 | 163.23 | 56.23 | 49.68 | -14.95 |
| *Punica granatum L.* | Yogeeta S *et al.*,2007**^(72)^** | INH and RIF | 50 | 87.39 |  |  | 400 | 47.52 | 100.00 | 45.62 | NA |
| *Saccharum officinarum L..* | Khan SW *et al.*,2015**^(73)^** | INH | 50 | 220.2 | NA | NA | 50 | 142.2 | 100.00 | 35.42 | NA |

***Supplementary table no.3: Effect of plant extracts on the ALP levels compared with toxic group (anti-TB drugs) and Silymarin (standard)***

| **Plant Scientific Name** | **Authors** | **Anti-TB drugs** | **Toxic group (anti-TB drugs) mg/kg** | | **Standard group (Silymarin) mg/kg** | | **Treatment group (PE)**  **mg/kg** | | **% protection by Standard (Silymarin) compared to the Toxic group** | **% protection by Treatment group (PE) compared to the Toxic group**  **(* Mean values are for the highest conc. of PE)** | **% protection by Treatment group (PE) compared to the Standard (Silymarin)** |
| --- | --- | --- | --- | --- | --- | --- | --- | --- | --- | --- | --- |
|  |  |  | **Conc. of drugs mg/kg** | **Mean mg/kg b. w** | **Conc. of Standard mg/kg** | **Mean mg/kg b. w** | **Conc. of PE mg/kg** | **Mean mg/kg b. w** |  |  |  |
| *Abelmoschus ficulneus L.* | Swetha *et* *al.,*2021 (**^10)^** | INH and RIF | 50 | 2.63 | 100 | 0.55 | 100,200, 400 | 1.13 | 79.09 | 57.03 | -105.45 |
| *Allium cepa L* | Sagar *et al*.,2022 **^(13)^** | INH and RIF | 50 | 0.15 | 100 | 0.14 | 100 | 0.19 | 6.67 | -26.67 | -35.71 |
| *Allium sativum L* | NASIM *et al*.,2017 **^(14)^** | INH | 50 | 2.46 | 200 | 0.62 | 250 | 1.32 | 74.80 | 46.34 | -112.90 |
| *Annona squamosa L.* | Uduman *et al.*,2011 **^(9)^** | INH and RIF | 100 | 1.06 | 2.5 | 0.42 | 250 and 500 | 0.84 | 60.38 | 20.75 | -100.00 |
| *Bacopa monnieri L.* | Evan *et al*.,2016 **^(17)^** | INH and RIF | 50 | 1.71 | 25 | 0.7 | 400 | 0.8 | 69.57 | 65.22 | -14.29 |
| *Carica papaya L.* | Aashish *et al*., 2013 **^(20)^** | INH and RIF | 125 | 2.54 | 100 | 0.63 | 300 and 600 | 0.82 | 100.00 | 39.34 | NA |
| *Cassia auriculata L.* | Ashish *et al*.,2014 **^(21)^** | INH, RIF and PZA | 54 | 0.54 | 50 | 141 | 20, 40, 60, and 100 | 1.87 | 63.16 | 52.05 | -30.16 |
| *Centella asiatica L.* | Kavisa *et al*.,2016 **^(22)^** | INH | 50 | 1.76 | 100 | 0.41 | 100, 200 and 400 | 0.4 | -5451.18 | 26.38 | 98.67 |
| *Cissampelos pareira L.* | Sangameswaran *et al*.,2012 **^(23)^** | INH and RIF | 50 | 1.08 | 50 | 1.03 | 500 | 1.09 | 24.07 | 25.93 | 2.44 |
| *Cissus quadrangularis L.* | SWAMY *et al*.,2012 **^(24)^** | RIF | 54 | 0.28 | 100 | 0.79 | 200 and 400 | 0.84 | 41.48 | 38.07 | -5.83 |
| *Ensete ventricosum Welw.* | Abebe *et al.*,2021 **^(27)^** | INH and RIF | 75 and 150 | 1.22 | 50 | 0.13 | 500 | 0.21 | 26.85 | 22.22 | -6.33 |
| *Hedyotis corymbosa L.* | Shah Marzia *et al.*,2018 **^(31)^** | INH and RIF | 50 | 3.13 | 100 | 0.31 | 125,250 and 500 | 0.55 | 53.57 | 25.00 | -61.54 |
| *Ipomoea carnea J.* | Ramesh Kumar *et al*.,2013 **^(33)^** | INH, RIF and PZA | 7.5, 10 and 35 | 2.02 | 2.5 | 1.02 | 100 and 400 | 2.61 | 100.00 | 14.89 | NA |
| *Ixora pavetta A.* | Jyothi Reddy *et al*.,2013 **^(34)^** | INH and RIF | 100 | 2.4 | 100 | 1.13 | 50 and 100 | 1.66 | 100.00 | 68.42 | NA |
| *Lawsonia inermis L.* | S. Sylvester *et al*.,2018 **^(36)^** | INH and RIF | 50 and 100 | 1.2 | 100 | 0.55 | 250 and 500 | 0.47 | 74.59 | 54.92 | -77.42 |
| *Nyctanthes arbor-tristis L.* | Chaudhary *et al*.,2018 **^(39)^** | INH, RIF and PZA | 7.5, 10 and 35 | 4.1 | 50 | 0.64 | 200 and 300 | 1.06 | 88.45 | 12.75 | -655.17 |
| *Ocimum canum Sims.* | Radhika J *et al*.,2018 **^(40)^** | INH | 54 | 0.67 | 50 | 1.67 | 200 and 400 | 3.71 | 67.41 | 16.61 | -155.88 |
| *Polygonatum cirrhifolium W.* | Roma Gha *et al*.,2023 **^(42)^** | INH and RIF | 50 | 4.52 | 50 | 0.32 | 250, 500 and 1000 | 0.45 | 44.06 | 17.82 | -46.90 |
| *Solanium nigrum L.* | Abdel-Hameed *et al.*,2019 **^(43)^** | INH and RIF | 50 | 1.77 | 50 | 4.16 | 250 and 500 | 3.36 | 77.08 | 80.42 | 14.55 |
| *Tamarindus indica L.* | Mohd Amir *et al*.,2015 **^(44)^** | INH and RIF | 50 and 100 | 17.8 | 50 | 0.62 | 125, 250 and 500 | 1.72 | 46.67 | 11.67 | -65.63 |
| *Telfairia occidentalis* | Legbosi Nwidu *et al.*,2018 **^(45)^** | INH and RIF | 100 | 0.2 | 100 | 8.8 | 250 and 375 | 8.5 | 59.27 | 9.51 | -122.16 |
| *Vernonia amygdalina Del* | Addisu Tilaye *et al*.,2018 **^(47)^** |  | 75 | 3.84 | 2.5 | 0.12 | 100, 250 and 500 | 0.15 | 100.00 | 22.83 | NA |
| *Ziziphus oenoplia L.* | Ch V Rao *et al.*,2012 **^(49)^** | INH and RIF | 50 | 1.9 | 5 ml | 0.85 | 200 | 1.1 | 52.24 | 32.84 | -40.63 |
| *Cucumis trigonus Roxb.* | Gopalakrishnan SB *et al.,*2015 (**^56)^** | INH and RIF | 50 | 2.05 | 100 | 0.96 | 125, 250 and 500 | 0.59 | 7.96 | 25.66 | 19.23 |
| *Embelia tsjeriam-cottam A.DC.* | Sambrekar SN *et al.,*2010 (**^57)^** | INH | 50 | 0.57 | 10 | 0.92 | 100, 200 and 300 | 0.53 | 64.97 | 2.82 | -177.42 |
| *Euphorbia fusiformis B.* | Anusuya *et al.*,2010 **^(58)^** | RIF | 1000 | 2.4 | 10 | 0.7 | 100, 200 and 300 | 0.08 | 50.56 | 52.25 | 3.41 |
| *Ficus benghalensis L.* | Parameswari SA *et al*.,2012 ^(59)^ | INH and RIF | 100 | 1.8 | 100 | 0.73 | 200 and 400 | 0.33 | 40.00 | 25.00 | -25.00 |
| *Ficus religiosa L.* | Parameswari SA *et al*.,2013 **^(60)^** | INH and RIF | 100 | 2.53 | 25 | 1.9 | 100,300 and 700 | 1.5 | 100.00 | 8.57 | NA |
| *Glinus lotoides L.* | Rameshwari KS et al.,2023 **^(61)^** | RIF | 100 | 0.86 | 200 | 0.7 | 200 and 400 | 1.1 | 30.33 | 9.84 | -29.41 |
| *Curcuma longa L. (Haridra Ghrit)* | Vyavahare T *et al.,*2024 **^(62)^** | INH, RIF and PZA | 100,300 and 700 | 2.63 | 200 | 0.79 | 100, 200 and 400 | 0.83 | 65.16 | 68.26 | 8.90 |
| *Leucas cephalotes R.* | Bais B et al., 2014 **^(63)^** | INH and RIF | 100 | 0.15 | 100 | 0.35 | 250 and 500 | 0.41 | 70.45 | 56.82 | -46.15 |
| *Mirabilis jalapa L.* | Jyothi BA *et al.,*2013 **^(65)^** | INH, RIF, PZA and EMB | 27, 40, 66 and 53 | 2.46 | 100 | 0.08 | 150 and 300 | 0.08 | 100.00 | 23.33 | NA |
| *Monotheca buxifolia F.* | Ullah I *et al.*,2016 **^(66)^** | INH and RIF | 50 | 1.06 | 200 | 0.4 | 150, 200 and 250 | 0.7 | 75.00 | 84.64 | 38.54 |
| *Moringa oleifera L.* | Pari *L et al*.,2002 **^(67)^** | INH, RIF and PZA | 7.5, 10 and 35 | 1.71 | 50 | 0.69 | 100,200 and 400 | 0.48 | 51.58 | 72.11 | 42.39 |
| *Mucuna pruriens L.* | Obogwu MB *et al.*,2014 **^(68)^** | INH and RIF | 100 | 2.54 | 100 | 1.75 | 200 and 400 | 0.58 | 65.85 | 96.10 | 88.57 |
| *Pongamia pinnata L.* | Derangula SS et al.,2022 **^(71)^** | INH and RIF | 27 and 40 | 0.54 | 100 | 0.06 | 250 and 500 | 0.12 | -28.07 | 42.11 | 54.79 |
| *Sesbania grandiflora L.* | Kuttiappan A *et al.,*2024 (**^74)^** | INH, RIF and PZA | 0.5 | 1.76 | 50 | 0.356 | 125, 250 and 500 | 0.403 | 20.83 | 37.50 | 21.05 |

***Supplementary table no. 4:*** ***Effect of plant extracts on the TBil levels compared with toxic group (anti-TB drugs) and Silymarin (standard)***

| **Plant Scientific Name** | **Authors** | **Anti-TB drugs** | **Toxic group (anti-TB drugs) mg/kg** | | **Standard group (Silymarin) mg/kg** | | **Treatment group (PE)**  **mg/kg** | | **% protection by Standard (Silymarin) compared to the Toxic group** | **% protection by Treatment group (PE) compared to the Toxic group**  **(* Mean values are for the highest conc. of PE)** | **% protection by Treatment group (PE) compared to the Standard (Silymarin)** |
| --- | --- | --- | --- | --- | --- | --- | --- | --- | --- | --- | --- |
|  |  |  | **Conc. of drugs mg/kg** | **Mean mg/kg b. w** | **Conc. of Standard mg/kg** | **Mean mg/kg b. w** | **Conc. of PE mg/kg** | **Mean mg/kg b. w** |  |  |  |
| *Abelmoschus ficulneus L.* | Swetha *et al.,*2021 **^(10)^** | INH and RIF | 50 | 1.63 | 100 | 5.95 | 100,200 , 400 | 3.24 | -265.03 | -98.77 | 45.55 |
| *Adenanthera pavonina L.* | Mujahid *et al*.,2013 **^(12)^** | INH and RIF | 50 | 1.61 | 100 | 3.22 | 100 and 200 | 2 | -100.00 | -24.22 | 37.89 |
| *Allium cepa L* | Sagar *et al*.,2022 **^(13)^** | INH and RIF | 50 | 24.53 | 100 | 37.9 | 100 | 27.7 | -54.50 | -12.92 | 26.91 |
| *Carica papaya L.* | Aashish *et al*., 2013 **^(20)^** | INH and RIF | 125 | 0.54 | 25 | 1.48 | 400 | 0.75 | 100.00 | -31.01 | NA |
| *Centella asiatica L.* | Kavisa *et al*.,2016 **^(22)^** | INH | 50 | 21.15 | 50 | 31.81 | 20, 40, 60, and 100 | 32.72 | -174.07 | -38.89 | 49.32 |
| *Cissus quadrangularisL.* | SWAMY *et al*.,2012 **^(24)^** | RIF | 54 | 3.51 | 50 | 6.48 | 500 | 6.39 | -50.40 | -54.70 | -2.86 |
| *Ipomoea carnea J.* | Ramesh Kumar *et al*.,2013 **^(33)^** | INH, RIF and PZA | 7.5, 10 and 35 | 3.41 | 100 | 6.03 | 125,250 and 500 | 4.77 | -84.62 | -82.05 | 1.39 |
| *Nyctanthes arbor-tristis L.* | Chaudhary *et al*.,2018 **^(39)^** | INH, RIF and PZA | 7.5, 10 and 35 | 0.47 | 100 | 1.24 | 250 and 500 | 0.72 | -135.59 | -42.37 | 39.57 |
| *Ocimum canum Sims.* | Radhika J *et al*.,2018 **^(40)^** | INH | 54 | 27 | 50 | 32.21 | 200 and 300 | 30.9 | 100.00 | -360.00 | NA |
| *Tamarindus indica L.* | Mohd Amir *et al*.,2015 **^(44)^** | INH and RIF | 50 and 100 | 0.32 | 50 | 0.69 | 250 and 500 | 0.37 | -76.83 | -39.88 | 20.90 |
| *Trapa natans L.* | Hussain *et al*.,2018 **^(46)^** | INH and RIF | 50 | 0.25 | 100 | 0.74 | 200 and 400 | 0.51 | 100.00 | -10.35 | NA |
| *Ziziphus oenoplia L.* | Ch V Rao *et al.*,2012 **^(49)^** | INH and RIF | 50 | 200.5 | 100 | 228.2 | 150 and 300 | 197.5 | -163.83 | -53.19 | 41.94 |
| *Cucumis trigonus Roxb.* | Gopalakrishnan SB *et al.,*2015 (**^56)^** | INH and RIF | 50 | 11.46 | 2.5 | 30.11 | 100, 250 and 500 | 16.91 | -19.30 | -14.44 | 4.07 |
| *Embelia tsjeriam-cottam A.DC.* | Sambrekar SN *et al*.,2010 **^(57)^** | INH | 50 | 50.35 | 5 ml | 84.79 | 200 | 81.56 | -115.63 | -15.63 | 46.38 |
| *Ficus benghalensis L.* | Parameswari SA *et al*.,2012 **^(59)^** | INH and RIF | 100 | 43.6 | 10 | 89.6 | 100, 200 and 300 | 87.27 | -196.00 | -104.00 | 31.08 |
| *Ficus religiosa L.* | Parameswari SA *et al.,*2013 **^(60)^** | INH and RIF | 100 | 18.3 | 10 | 19.3 | 100, 200 and 300 | 15.5 | -13.82 | 1.50 | 13.45 |
| *Leucas cephalotes R.* | Bais B *et al.,* 2014 **^(62)^** | INH and RIF | 100 | 0.42 | 200 | 1.58 | 200 and 400 | 1.32 | 100.00 | -21.36 | NA |
| *Luffa acutangula L.* | Jadhav VB *et al.,*2010 **^(64)^** | RIF | 100 | 2.8 | 200 | 8 | 100, 200 and 400 | 5.1 | -162.74 | -47.56 | 43.84 |
| *Mirabilis jalapa L.* | Jyothi BA *et al.*,2013 **^(65)^** | INH, RIF, PZA and EMB | 27, 40, 66 and 53 | 0.74 | 100 | 2.27 | 250 and 500 | 1.14 | -68.40 | -61.99 | 3.81 |
| *Mucuna pruriens L.* | Obogwu MB *et al*.,2014 **^(68)^** | INH and RIF | 100 | 0.435 | 50 | 0.793 | 100,200 and 400 | 0.742 | -105.50 | -100.16 | 2.60 |
| *Pongamia pinnata L.* | Derangula SS *et al.*,2022 **^(71)^** | INH and RIF | 27 and 40 | 0.79 | 100 | 3.12 | 200 and 400 | 1.23 | -5.46 | 15.30 | 19.69 |

***Supplementary table no.5:***  ***Effect of plant extracts on the GSH levels compared with toxic group (anti-TB drugs) and Silymarin (standard)***

| **Plant Scientific Name** | **Authors** | **Anti-TB drugs** | **Toxic group (anti-TB drugs) mg/kg** | | **Standard group (Silymarin) mg/kg** | | **Treatment group (PE)**  **mg/kg** | | **% protection by Standard (Silymarin) compared to the Toxic group** | **% protection by Treatment group (PE) compared to the Toxic group**  **(* Mean values are for the highest conc. of PE)** | **% protection by Treatment group (PE) compared to the Standard (Silymarin)** |
| --- | --- | --- | --- | --- | --- | --- | --- | --- | --- | --- | --- |
|  |  |  | **Conc. of drugs mg/kg** | **Mean mg/kg b. w** | **Conc. of Standard mg/kg** | **Mean mg/kg b. w** | **Conc. of PE mg/kg** | **Mean mg/kg b. w** |  |  |  |
| *Abelmoschus ficulneus L.* | Swetha *et* *al.,*2021 **^(10)^** | INH and RIF | 50 | 3.35 | 100 | 14.58 | 100,200, 400 | 6.74 | -335.22 | -101.19 | 53.77 |
| *Adenanthera pavonina L.* | Mujahid *et al*.,2013 **^(12)^** | INH and RIF | 50 | 8.66 | 100 | 14.32 | 100 and 200 | 11.04 | -65.36 | -27.48 | 22.91 |
| *Annona squamosa L.* | Uduman *et al.*,2011 **^(9)^** | INH and RIF | 100 | 0.32 | 2.5 | 0.98 | 250 and 500 | 1 | -206.25 | -212.50 | -2.04 |
| *Cnidoscolus chayamansa (McVaugh)* | Mariana *et al.*,2019 **^(25)^** | INH, RIF, and PZA | 50 and 100 | 895.3 | 2.5 | 555.1 | 50, 50 and 100 | 544.8 | 38.00 | 39.15 | 1.86 |
| *Alpinia officinarum H.* | Shimaila *et al*.,2019 **^(29)^** | INH and RIF | 50 | 17.7 | 100 | 41.3 | 200 and 400 | 30.4 | -133.33 | -71.75 | 26.39 |
| *Hibiscus vitifolius L.* | Anbu Jeba *et al*.,2012 **^(32)^** | INH, RIF and PZA | 7.5, 10 and 35 | 7.77 | 100 | 13.23 | 400 | 8.21 | 100.00 | -69.38 | NA |
| *Ipomoea carnea J.* | Ramesh Kumar *et al*.,2013 **^(33)^** | INH, RIF and PZA | 7.5, 10 and 35 | 31.41 | 100 | 44.8 | 125,250 and 500 | 33.52 | -70.27 | -5.66 | 37.94 |
| *Nyctanthes arbor-tristis L.* | Chaudhary *et al*.,2018 **^(39)^** | INH, RIF and PZA | 7.5, 10 and 35 | 28.75 | 100 | 53.8 | 250 and 500 | 36.8 | -42.63 | -6.72 | 25.18 |
| *Tamarindus indica L.* | Mohd Amir *et al*.,2015 **^(44)^** | INH and RIF | 50 and 100 | 2.33 | 50 | 8.02 | 250 and 500 | 2.75 | 100.00 | -238.89 | NA |
| *Trapa natans L.* | Hussain *et al*.,2018 **^(46)^** | INH and RIF | 50 | 17.84 | 100 | 46.55 | 200 and 400 | 33.74 | -87.13 | -28.00 | 31.60 |
| *Ziziphus oenoplia L.* | Ch V Rao *et al.*,2012 **^(49)^** | INH and RIF | 50 | 12.72 | 100 | 18.44 | 150 and 300 | 14.02 | -244.21 | -18.03 | 65.71 |
| *Cucumis trigonus Roxb.* | Gopalakrishnan SB *et al.,*2015 (**^56)^** | INH and RIF | 50 | 1.583 | 2.5 | 3.91 | 100, 250 and 500 | 2.671 | -160.93 | -89.13 | 27.52 |
| *Embelia tsjeriam-cottam A.DC.* | Sambrekar SN *et al.,*2010 **^(57)^** | INH | 50 | 92.75 | 5 ml | 254.5 | 200 | 246.4 | -44.97 | -10.22 | 23.97 |
| *Luffa acutangula L.* | Jadhav VB *et al.*,2010 **^(64)^** | RIF | 100 | 15.9 | 200 | 37.04 | 100, 200 and 400 | 33.11 | 100.00 | -9.87 | NA |
| *Mirabilis jalapa L.* | Jyothi BA *et al.,*2013 **^(65)^** | INH, RIF, PZA and EMB | 27, 40, 66 and 53 | 15.49 | 100 | 33.89 | 250 and 500 | 23.44 | -147.00 | -68.73 | 31.69 |
| *Punica granatum L.* | Yogeeta S *et al*.,2007 **^(72)^** | INH and RIF | 50 | 3.536 | NA | NA | 400 | 4.43 | 100.00 | -25.28 | NA |

***Supplementary table no.6: Effect of plant extracts on the CAT levels compared with toxic group (anti-TB drugs) and Silymarin (standard)***

| **Plant Scientific Name** | **Authors** | **Anti-TB drugs** | **Toxic group (anti-TB drugs) mg/kg** | | **Standard group (Silymarin) mg/kg** | | **Treatment group (PE)**  **mg/kg** | | **% protection by Standard (Silymarin) compared to the Toxic group** | **% protection by Treatment group (PE) compared to the Toxic group**  **(* Mean values are for the highest conc. of PE)** | **% protection by Treatment group (PE) compared to the Standard (Silymarin)** |
| --- | --- | --- | --- | --- | --- | --- | --- | --- | --- | --- | --- |
|  |  |  | **Conc. of drugs mg/kg** | **Mean mg/kg b. w** | **Conc. of Standard mg/kg** | **Mean mg/kg b. w** | **Conc. of PE mg/kg** | **Mean mg/kg b. w** |  |  |  |
| *Abelmoschus ficulneus L.* | Swetha *et al*.,2021 **^(10)^** | INH and RIF | 50 | 5.24 | 100 | 11.63 | 100, 200, 400 | 7.56 | -44.27 | 35.00 | -44.27 |
| *Adenanthera pavonina L.* | Mujahid *et al*.,2013 **^(12)^** | INH and RIF | 50 | 1.48 | 100 | 4.24 | 100 and 200 | 2.33 | -57.43 | 45.05 | -57.43 |
| *Allium cepa L* | Sagar *et al*.,2022 **^(13)^** | INH and RIF | 50 | 427.65 | 100 | 506.46 | 100 | 496.07 | -16.00 | 2.05 | -16.00 |
| *Cassia auriculata L.* | Ashish *et al*.,2014 (**^21)^** | INH, RIF and PZA | 54 | 1.45 | 100 | 0.75 | 300 and 600 | 0.44 | -173.79 | NA | -173.79 |
| *Centella asiatica L.* | Kavisa *et al*.,2016 **^(22)^** | INH | 50 | 3.27 | 50 | 8.41 | 20, 40, 60, and 100 | 8.43 | -176.94 | -28.38 | -176.94 |
| *Cissus quadrangularis L.* | SWAMY *et al*.,2012 **^(24)^** | RIF | 54 | 6.01 | 50 | 11 | 500 | 10.13 | -157.80 | -0.24 | -157.80 |
| *Cnidoscolus chayamansa (McVaugh)* | Mariana *et al.*,2019 **^(25)^** | INH, RIF and PZA | 50 and 100 | 465.67 | 2.5 | 334.33 | 50, 50 and 100 | 213.93 | -68.55 | 7.91 | -68.55 |
| *Alpinia officinarum H.* | Shimaila *et al*.,2019 **^(29)^** | INH and RIF | 50 | 1.24 | 100 | 4.89 | 200 and 400 | 3.63 | 54.06 | 36.01 | 54.06 |
| *Hibiscus vitifolius L.* | Anbu Jeba *et al*.,2012 **^(32)^** | INH, RIF and PZA | 7.5, 10 and 35 | 0.893 | 100 | 3.49 | 400 | 0.98 | -192.74 | 25.77 | -192.74 |
| *Ipomoea carnea J.* | Ramesh Kumar *et al*.,2013 **^(33)^** | INH, RIF and PZA | 7.5, 10 and 35 | 12.73 | 100 | 21.32 | 125,250 and 500 | 13.21 | -157.04 | NA | -157.04 |
| *Nyctanthes arbor-tristis L.* | Chaudhary *et al*.,2018 **^(39)^** | INH, RIF and PZA | 7.5, 10 and 35 | 8.9 | 100 | 21.9 | 250 and 500 | 13.1 | -9.74 | 71.92 | -9.74 |
| *Ocimum canum Sims.* | Radhika J *et al*.,2018 **^(40)^** | INH | 54 | 0.87 | 50 | 1.23 | 200 and 300 | 1.36 | -3.77 | 38.04 | -3.77 |
| *Trapa natans L.* | Hussain *et al*.,2018 **^(46)^** | INH and RIF | 50 | 9.52 | 100 | 4.18 | 200 and 400 | 1.84 | -57.59 | NA | -57.59 |
| *Ziziphus oenoplia L.* | Ch V Rao *et al.*,2012 **^(49)^** | INH and RIF | 50 | 16.04 | 100 | 18.87 | 150 and 300 | 13.83 | -47.19 | 40.18 | -47.19 |
| *Cucumis trigonus Roxb.* | Gopalakrishnan SB *et al.,*2015 (**^56)^** | INH and RIF | 50 | 0.073 | 2.5 | 21.78 | 100, 250 and 500 | 19.84 | -56.32 | -10.57 | -56.32 |
| *Embelia tsjeriam-cottam A.DC.* | Sambrekar SN *et al.,*2010 **^(57)^** | INH | 50 | 19.16 | 5 ml | 0.203 | 200 | 0.129 | -4.55 | 55.98 | -4.55 |
| *Leucas cephalotes R.* | Bais B *et al.,* 2014 **^(63)^** | INH and RIF | 100 | 19.21 | 200 | 32.92 | 200 and 400 | 36.3 | -45.27 | 26.71 | -45.27 |
| *Mirabilis jalapa L.* | Jyothi BA *et al.*,2013 **^(65)^** | INH, RIF, PZA and EMB | 27, 40, 66 and 53 | 1.62 | 100 | 35.06 | 250 and 500 | 31.24 | -23.69 | 8.91 | -23.69 |
| *Moringa oleifera L.* | Pari L *et al.,*2002 **^(67)^** | INH, RIF and PZA | 7.5, 10 and 35 |  | 200 | 3.71 | 150, 200 and 250 | 2.08 | -13.77 | NA | -13.77 |
| *Mucuna pruriens L.* | Obogwu MB *et al*.,2014 **^(68)^** | INH and RIF | 100 | 1.072 | 50 | 1.945 | 100,200 and 400 | 2.203 | -76.71 | 36.45 | -76.71 |
| *Sesbania grandiflora L.* | Kuttiappan A *et al.,*2024 **^(74)^** | INH, RIF and PZA | 0.5 | 4.17 | 100 | 4.12 | 250 and 500 | 3.12 | -89.46 | -10.27 | -89.46 |

***Supplementary table no 7: Effect of plant extracts on the SOD levels compared with toxic group (anti-TB drugs) and Silymarin (standard)***

***Spplementary table no. 8: Quality Assessment of the included study using SYRCLE SCALE***

| **Author** | **Did the investigators describe a random component in the sequence generation process?** | **Was the distribution of relevant baseline characteristics balanced for the intervention and control groups?** | **If relevant, did the investigator adequately adjust for unequal distribution of some relevant baseline characteristics in the analysis?** | **Was the timing of disease induction adequate?** | **Could the investigator allocating the animals to intervention or control group not foresee assignment due to one of the following or equivalent methods?** | **Did the authors randomly place the cages or animals within the animal room/facility?** | **Is it unlikely that the outcome or the outcome measurement was influenced by not randomly housing the animals?** | **Did the investigators randomly pick an animal during outcome assessment, or did they use a random component in the sequence generation for outcome assessment?** | **Was blinding of outcome assessor ensured, and was it unlikely that blinding could have been broken?** | **Was the outcome assessor not blinded, but do review authors judge that the outcome is not likely to be influenced by lack of blinding?** | **Were all animals included in the analysis?** | **Were the reason for missing outcome data unlikely to be related to true outcome?** | **Was the study protocol available and were all of the study's pre-specified primary and secondary outcomes reported in the current manuscript?** |
| --- | --- | --- | --- | --- | --- | --- | --- | --- | --- | --- | --- | --- | --- |
| Swetha *et al.,*2021 | Unclear | Yes (sex and weight) | unclear | unclear | unclear | yes | unclear | yes (randomly) | Yes | unclear | yes | yes | yes |
| Mujahid *et al.*, 2013 | Unclear | Yes (sex and weight) | unclear | unclear | unclear | yes | unclear | unclear | yes | unclear | yes | yes | yes |
| Sagar *et al.,*2022 | Unclear | Yes (sex and weight) | unclear | unclear | unclear | yes | unclear | yes (randomly) | Yes | unclear | yes | yes | yes |
| NASIM *et al*.,2011 | Unclear | yes (sex and weight) | unclear | unclear | unclear | yes | unclear | yes (randomly) | Yes | unclear | yes | yes | yes |
| MOHAMED *et al.*,2011 | Unclear | Yes (sex and weight) | unclear | unclear | unclear | yes | unclear | yes (randomly) | Yes | unclear | yes | yes | yes |
| Jibril M *et al.,*2015 | Unclear | Yes (sex and weight) | unclear | unclear | unclear | yes | unclear | yes (randomly) | Yes | unclear | yes | yes | yes |
| Maha A *et al.*, 2019 | Unclear | Yes (sex and weight) | unclear | unclear | unclear | yes | unclear | yes (randomly) | Yes | unclear | yes | yes | yes |
| Sabina *et al*.,2016 | Unclear | Yes (sex and weight) | unclear | unclear | unclear | yes | unclear | yes (randomly) | Yes | unclear | yes | yes | yes |
| Noor *et al.*,2015 | Unclear | Yes (sex and weight) | unclear | unclear | unclear | yes | unclear | yes (randomly) | Yes | unclear | yes | yes | yes |
| Angeli B *et al.,*2014 | Unclear | Yes (sex and weight) | unclear | unclear | unclear | yes | unclear | yes (randomly) | Yes | unclear | yes | yes | yes |
| Aashish *et al.*,2013 | Unclear | Yes (sex and weight) | unclear | unclear | unclear | yes | unclear | yes (randomly) | Yes | unclear | yes | yes | yes |
| Ashish V *et al.*,2013 | Unclear | Yes (sex and weight) | unclear | unclear | unclear | yes | unclear | yes (randomly) | Yes | unclear | yes | yes | yes |
| Kavisa *et al*.,2016 | Unclear | Yes (sex and weight) | unclear | unclear | unclear | yes | unclear | yes (randomly) | Yes | unclear | yes | yes | yes |
| Sangameswaran *et al.*,2016 | Unclear | Yes (sex and weight) | unclear | unclear | unclear | yes | unclear | yes (randomly) | Yes | unclear | yes | yes | yes |
| SWAMY *et al*.,2012 | Unclear | Yes (sex and weight) | unclear | unclear | unclear | yes | unclear | yes (randomly) | Yes | unclear | yes | yes | yes |
| Mariana Z *et al.,*2019 | Unclear | Yes (sex and weight) | unclear | unclear | unclear | yes | unclear | yes (randomly) | Yes | unclear | yes | yes | yes |
| Kenedy *et al.*,2022 | Unclear | Yes (sex and weight) | unclear | unclear | unclear | yes | unclear | yes (randomly) | Yes | unclear | yes | yes | yes |
| Abebe *et al.,* 2021 | Unclear | Yes (sex and weight) | unclear | unclear | unclear | yes | unclear | yes (randomly) | Yes | unclear | yes | yes | yes |
| N. Y. GOND *et al.*,2008 | Unclear | yes (sex and weight) | unclear | unclear | unclear | yes | unclear | yes (randomly) | Yes | unclear | yes | yes | yes |
| Shimaila*et al*.,2019 | Unclear | Yes (sex and weight) | unclear | unclear | unclear | yes | unclear | yes (randomly) | Yes | unclear | yes | yes | yes |
| Shimaila *et al.,*2014 | Unclear | Yes (sex and weight) | unclear | unclear | unclear | yes | unclear | yes (randomly) | Yes | unclear | yes | yes | yes |
| Nasiru A *et al.,*2012 | Unclear | unclear | unclear | unclear | unclear | yes | unclear | yes (randomly) | Yes | unclear | yes | yes | yes |
| Shah Marzia *et al.*,2018 | Unclear | Yes (sex and weight) | unclear | unclear | unclear | yes | unclear | yes (randomly) | Yes | unclear | yes | yes | yes |
| Anbu *et al.,*2012 | Unclear | Yes (sex and weight) | unclear | unclear | unclear | yes | unclear | yes (randomly) | Yes | unclear | yes | yes | yes |
| Ramesh *et al.,*2013 | Unclear | Yes (sex and weight) | unclear | unclear | unclear | yes | unclear | yes (randomly) | Yes | unclear | yes | yes | yes |
| Jyothi *et al.,*2013 | Unclear | Yes (sex and weight) | unclear | unclear | unclear | yes | unclear | yes (randomly) | Yes | unclear | yes | yes | yes |
| Netranjali *et al.*,2012 | Unclear | unclear | unclear | unclear | unclear | yes | unclear | yes (randomly) | Yes | unclear | yes | yes | yes |
| Sylvester *et al.*, 2018 | Unclear | Yes (sex and weight) | unclear | unclear | unclear | yes | unclear | yes (randomly) | Yes | unclear | yes | yes | yes |
| Shabbir *et al.*, 2020 | Unclear | Yes (sex and weight) | unclear | unclear | unclear | yes | unclear | yes (randomly) | Yes | unclear | yes | yes | yes |
| Hafeez *et al.,* 2019 | Unclear | Yes (sex and weight) | unclear | unclear | unclear | yes | unclear | yes (randomly) | Yes | unclear | yes | yes | yes |
| Sachin *et al.*,2017 | Unclear | Yes (sex and weight) | unclear | unclear | unclear | yes | unclear | yes (randomly) | Yes | unclear | yes | yes | yes |
| Radhika *et al.,* 2017 | Unclear | Yes (sex and weight) | unclear | unclear | unclear | yes | unclear | yes (randomly) | Yes | unclear | yes | yes | yes |
| Gugsa *et al.,*2023 | Unclear | Yes (sex and weight) | unclear | unclear | unclear | yes | unclear | yes (randomly) | Yes | unclear | yes | yes | yes |
| Roma *et al.,*2023 | Unclear | Yes (sex and weight) | unclear | unclear | unclear | yes | unclear | yes (randomly) | Yes | unclear | yes | yes | yes |
| Siordia *et al.,* 2021 | Unclear | Yes (sex and weight) | unclear | unclear | unclear | yes | unclear | yes (randomly) | Yes | unclear | yes | yes | yes |
| Abdel *et al.,*2019 | Unclear | Yes (sex and weight) | unclear | unclear | unclear | yes | unclear | yes (randomly) | Yes | unclear | yes | yes | yes |
| Mohd Amir *et al.,*2015 | Unclear | unclear | unclear | unclear | unclear | yes | unclear | yes (randomly) | Yes | unclear | yes | yes | yes |
| Lucky Legbos *et al*.,2018 | Unclear | Yes (sex and weight) | unclear | unclear | unclear | yes | unclear | yes (randomly) | Yes | unclear | yes | yes | yes |
| Hussain *et al,.*2017 | Unclear | Yes (sex and weight) | unclear | unclear | unclear | yes | unclear | yes (randomly) | Yes | unclear | yes | yes | yes |
| Tilaye *et al.,*2018 | Unclear | Yes (sex and weight) | unclear | unclear | unclear | yes | unclear | yes (randomly) | Yes | unclear | yes | yes | yes |
| Vishal R *et al.*,2008 | Unclear | Yes (sex and weight) | unclear | unclear | unclear | yes | unclear | yes (randomly) | Yes | unclear | yes | yes | yes |
| Ch V Rao *et al.*, 2012 | Unclear | Yes (sex and weight) | unclear | unclear | unclear | yes | unclear | yes (randomly) | Yes | unclear | yes | yes | yes |
| HIMAJA N *et al*.,2015 | Unclear | Yes (sex and weight) | unclear | unclear | unclear | yes | unclear | yes (randomly) | Yes | unclear | yes | yes | yes |
| Ishtiaq S *et al*.,2017 | Unclear | Yes (sex and weight) | unclear | unclear | unclear | yes | unclear | yes (randomly) | Yes | unclear | yes | yes | yes |
| N Palanisamy *et al.*,2012 | Unclear | Yes (sex and weight) | unclear | unclear | unclear | yes | unclear | yes (randomly) | Yes | unclear | yes | yes | yes |
| Lina SM *et al.*,2012 | Unclear | Yes (sex and weight) | unclear | unclear | unclear | yes | unclear | yes (randomly) | Yes | unclear | yes | yes | yes |
| Kosasih E *et al*.,2019 | Unclear | Yes (sex and weight) | unclear | unclear | unclear | yes | unclear | yes (randomly) | Yes | unclear | yes | yes | yes |
| Wali AF *et al.*,2020 | Unclear | Yes (sex and weight) | unclear | unclear | unclear | yes | unclear | yes (randomly) | Yes | unclear | yes | yes | yes |
| Gopalakrishnan SB *et al*.,2015 | Unclear | Yes (sex and weight) | unclear | unclear | unclear | yes | unclear | yes (randomly) | Yes | unclear | yes | yes | yes |
| Sambrekar SN *et al*.,2010 | Unclear | Yes (sex and weight) | unclear | unclear | unclear | yes | unclear | yes (randomly) | Yes | unclear | yes | yes | yes |
| Anusuya *et al*.,2010 | Unclear | Yes (sex and weight) | unclear | unclear | unclear | yes | unclear | yes (randomly) | Yes | unclear | yes | yes | yes |
| Parameswari SA *et al.*,2012 | Unclear | Yes (sex and weight) | unclear | unclear | unclear | yes | unclear | yes (randomly) | Yes | unclear | yes | yes | yes |
| Parameswari SA *et al.*,2013 | Unclear | Yes (sex and weight) | unclear | unclear | unclear | yes | unclear | yes (randomly) | Yes | unclear | yes | yes | yes |
| Rameshwari KS *et al.*,2023 | Unclear | Yes (sex and weight) | unclear | unclear | unclear | yes | unclear | yes (randomly) | Yes | unclear | yes | yes | yes |
| Vyavahare T et al.,2024 | Unclear | unclear | unclear | unclear | unclear | yes | unclear | yes (randomly) | Yes | unclear | yes | yes | yes |
| Bais B *et al.,* 2014 | Unclear | Yes (sex and weight) | unclear | unclear | unclear | yes | unclear | yes (randomly) | Yes | unclear | yes | yes | yes |
| Jadhav VB *et al.,*2010 | Unclear | Yes (sex and weight) | unclear | unclear | unclear | yes | unclear | yes (randomly) | Yes | unclear | yes | yes | yes |
| Jyothi BA *et al*.,2013 | Unclear | Yes (sex and weight) | unclear | unclear | unclear | yes | unclear | yes (randomly) | Yes | unclear | yes | yes | yes |
| Ullah I *et al*.,2016 | Unclear | Yes (sex and weight) | unclear | unclear | unclear | yes | unclear | yes (randomly) | Yes | unclear | yes | yes | yes |
| Pari L *et al.*,2002 | Unclear | Yes (sex and weight) | unclear | unclear | unclear | yes | unclear | yes (randomly) | Yes | unclear | yes | yes | yes |
| Obogwu MB *et al*.,2014 | Unclear | Yes (sex and weight) | unclear | unclear | unclear | yes | unclear | yes (randomly) | Yes | unclear | yes | yes | yes |
| Ocimum P *et al.*,2003 | Unclear | Yes (sex and weight) | unclear | unclear | unclear | yes | unclear | yes (randomly) | Yes | unclear | yes | yes | yes |
| Anand K *et al.*,2014 | Unclear | Yes (sex and weight) | unclear | unclear | unclear | yes | unclear | yes (randomly) | Yes | unclear | yes | yes | yes |
| Derangula SS *et al.*,2022 | Unclear | Yes (sex and weight) | unclear | unclear | unclear | yes | unclear | yes (randomly) | Yes | unclear | yes | yes | yes |
| Yogeeta S *et al.*,2007 | Unclear | Yes (sex and weight) | unclear | unclear | unclear | yes | unclear | yes (randomly) | Yes | unclear | yes | yes | yes |
| Khan SW *et al.,*2015 | Unclear | Yes (sex and weight) | unclear | unclear | unclear | yes | unclear | yes (randomly) | yes | unclear | yes | yes | yes |
| Kuttiappan A *et al*.,2024 | Unclear | Yes (sex and weight) | unclear | unclear | unclear | yes | unclear | yes (randomly) | Yes | unclear | yes | yes | yes |
| Nwidu LL *et al.*,2019 | Unclear | Yes (sex and weight) | unclear | unclear | unclear | yes | unclear | yes (randomly) | Yes | unclear | yes | yes | yes |
